# Meiosis-specific cohesin complexes display essential and distinct roles in mitotic embryonic stem cell chromosomes

**DOI:** 10.1186/s13059-022-02632-y

**Published:** 2022-03-03

**Authors:** Eui-Hwan Choi, Seobin Yoon, Young Eun Koh, Tae Kyung Hong, Jeong Tae Do, Bum-Kyu Lee, Yoonsoo Hahn, Keun P. Kim

**Affiliations:** 1 Department of Life Sciences, Chung-Ang University, Seoul, 06974 South Korea; 2 Department of Stem Cell and Regenerative Biotechnology, Konkuk Institute of Technology, Konkuk University, Seoul, 05029 South Korea; 3 Department of Biomedical Sciences, Cancer Research Center, University of Albany-State University of New York, Rensselaer, NY USA

**Keywords:** Meiosis, Mitosis, Embryonic stem cell, Cohesin, Chromosome

## Abstract

**Background:**

Cohesin is a chromosome-associated SMC–kleisin complex that mediates sister chromatid cohesion, recombination, and most chromosomal processes during mitosis and meiosis. However, it remains unclear whether meiosis-specific cohesin complexes are functionally active in mitotic chromosomes.

**Results:**

Through high-resolution 3D-structured illumination microscopy (3D-SIM) and functional analyses, we report multiple biological processes associated with the meiosis-specific cohesin components, α-kleisin REC8 and STAG3, and the distinct loss of function of meiotic cohesin during the cell cycle of embryonic stem cells (ESCs). First, we show that STAG3 is required for the efficient localization of REC8 to the nucleus by interacting with REC8. REC8-STAG3-containing cohesin regulates topological properties of chromosomes and maintains sister chromatid cohesion. Second, REC8-cohesin has additional sister chromatid cohesion roles in concert with mitotic RAD21-cohesin on ESC chromosomes. SIM imaging of REC8 and RAD21 co-staining revealed that the two types of α-kleisin subunits exhibited distinct loading patterns along ESC chromosomes. Third, knockdown of REC8 or RAD21-cohesin not only leads to higher rates of premature sister chromatid separation and delayed replication fork progression, which can cause proliferation and developmental defects, but also enhances chromosome compaction by hyperloading of retinoblastoma protein–condensin complexes from the prophase onward.

**Conclusions:**

Our findings indicate that the delicate balance between mitotic and meiotic cohesins may regulate ESC-specific chromosomal organization and the mitotic program.

**Supplementary Information:**

The online version contains supplementary material available at 10.1186/s13059-022-02632-y.

## Background

Embryonic stem cells (ESCs) require specific components of the cell cycle machinery to maintain self-renewal ability and pluripotency [1]. ESCs undergo dynamic changes in their chromatin landscape to express diverse genes, as well as global chromosome condensation upon lineage differentiation [1, 2]. ESCs and differentiated cells are notably different during mitosis. For example, ESCs have self-renewal capabilities, express high levels of DNA repair proteins, and proliferate rapidly with a prolonged S phase during the cell cycle [3–6]. As such, ESCs display a high tolerance for endogenous replication and DNA damage stress [4, 7, 8]. However, little is known about how ESCs maintain genome integrity and cope with the chromosomal abnormalities and replication stresses that may occur during cell proliferation and differentiation.

**Fig 1.**
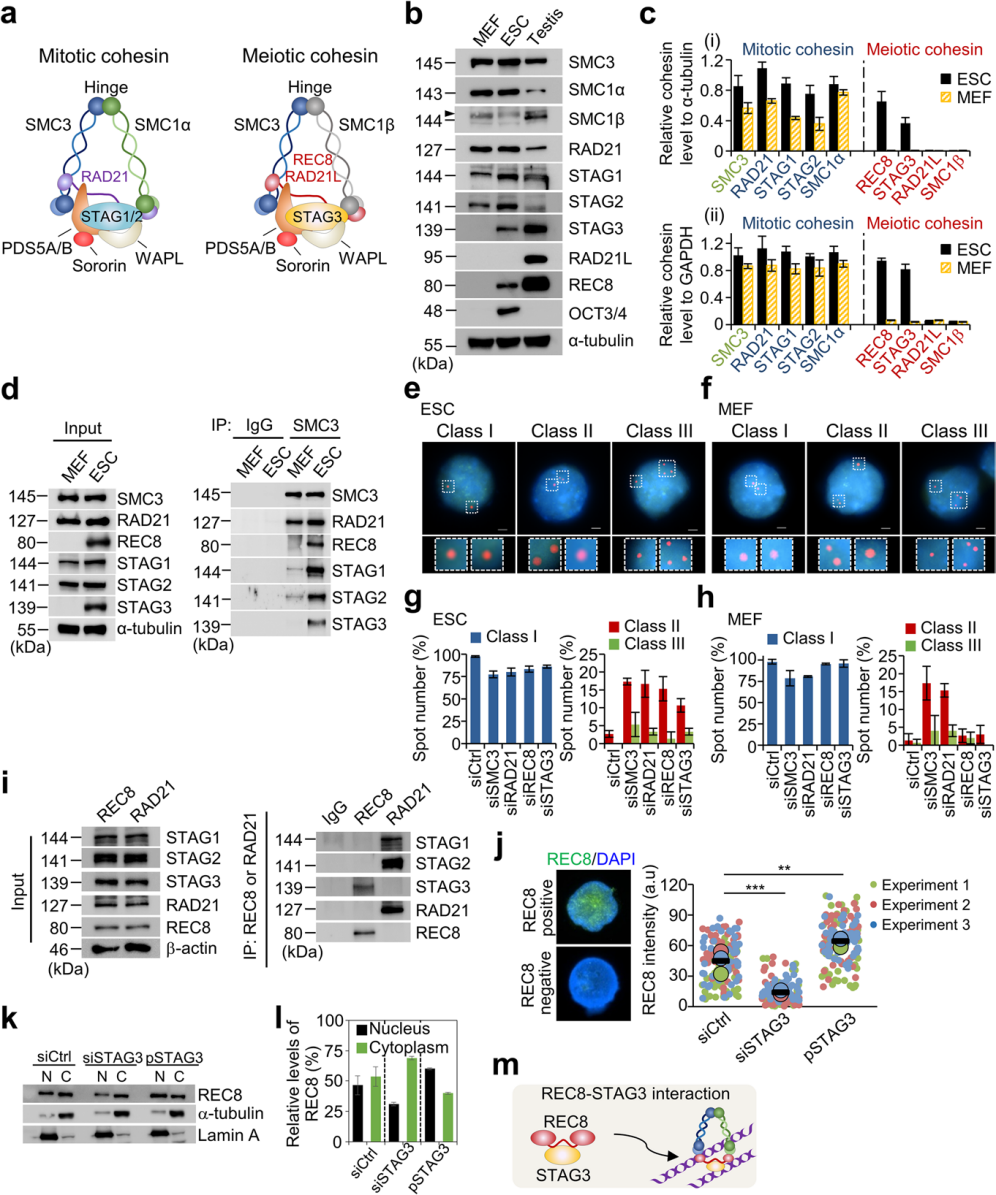
Expression and localization of meiotic cohesin components in ESCs. **a** Cohesin complexes in mitosis and meiosis. The cohesin complex is composed of long coiled-coil SMC subunits, referred to as SMC3 and SMC1α for mitotic cohesins, and SMC3 and SMC1β for meiotic cohesins [20, 21]. SMC complexes combined with the α-kleisin subunits (RAD21 for mitosis and REC8/RAD21L for meiosis) form a V-shaped structure [20, 21]. Cohesin subunits also include STAG1/2 for mitotic cohesin and STAG3 for meiotic cohesin, as well as PDS5A/B, Sororin, and WAPL, all of which are directly involved in the formation of cohesin complexes. **b** Expression analysis of cohesin components. Whole-cell lysate samples were extracted from asynchronous MEFs, ESCs (J1), and testis of C57BL/6J mice. α-tubulin was used as a loading control. The arrowhead indicates a non-specific band. **c** Comparison of expression level of SMC3, RAD21, SMC1α, STAG1, STAG2, REC8, RAD21L, STAG3, and SMC1β between ESCs and MEF, as assessed by (i) western blotting and (ii) qPCR analyses. **d** Immunoprecipitation (IP) analysis in MEFs and ESCs. Cohesin subunits were pulled down the using anti-cohesin (SMC3, RAD21, REC8, STAG1, STAG2, and STAG3) antibodies from whole-cell extracts in MEFs and ESCs. **e,f** Representative images of the interphase FISH analysis for the detection of sister chromatid separation in ESCs and MEFs. The signals of the locus-specific probe, bound to the arm regions (Chromosome 4: 116,094,264–116,123,690), are shown in red and DAPI-counterstained chromosomes are shown in blue. Scale bars = 2.5 μm. **g,h** Quantification of spots in each nucleus. The error bars represent the mean ± SD value from three biological replicates (*n* ≥ 50 for condition). **i** Physical interaction between REC8 and STAG3 in ESCs as demonstrated by IP analysis. **j** Quantification of REC8 intensity in the siCtrl, siSTAG3, and STAG3 expression vector (pSTAG3) (*n* = 130). Each biological replicate is color-coded (red, green, and blue) and the average of each replicated data is indicated with a larger dot, and black bars indicate the averages of three means (*n* ≥ 50 for condition). The error bars are the mean ± SD from three independent biological replicates. a.u., arbitrary unit. *P*-values (paired two-tailed t-test) were calculated using GraphPad Prism 5 software; **P < 0.01, ***P < 0.001. **k** Analysis of REC8 localization in the presence or absence of STAG3. α-tubulin and Lamin B were used as cytoplasmic and nuclear protein loading markers, respectively. pSTAG3, STAG3 expression vector; N, nucleus; C, cytoplasm. **l** Quantification of REC8 levels in cytoplasm and nucleus. The error bars are the mean ± SD from the biological replicates. **m** STAG3 binds directly to REC8 and maintains stabilization of REC8. The REC8–STAG3 complexes translocate into the nucleus by direct physical interaction

Cohesion between sister chromatids allows precise chromosome morphogenesis, DNA replication, recombination, and transcriptional regulation [9–12]. Although cohesin was initially identified for its role in sister chromatid cohesion, cohesin has since been linked to a variety of cellular processes, including DNA damage repair and formation of topologically associating domains via “loop extrusion” during the interphase of the basic cell cycle [11, 13–19]. Cohesin is therefore an important candidate for maintaining genome integrity and managing topological changes of chromosomes in ESCs. Cohesin forms V-shaped structures that link the sister chromatids together from S phase to anaphase [20, 21] (Fig. 1a). Most eukaryotic cells express three α-kleisin proteins, RAD21 (known as Scc1/Mcd1 in budding yeast), RAD21L, and REC8 [11, 12, 22]. RAD21 is expressed in both mitotic and meiotic cells. In contrast, REC8 and RAD21L are known to be expressed only during early meiosis [11, 22]. RAD21 binds to the two orthologous stromal antigen (STAG) subunits, STAG1 and STAG2, which further interact with a heterodimer composed of PDS5 and WAPL [23–25]. The cohesin complex consists of 50-nm coiled-coil structural maintenance of chromosomes (SMC) subunits (SMC1α and SMC3 for mitosis; SMC1β and SMC3 for meiosis) that join together to form a hinge domain and an α-kleisin subunit that interacts with diverse cohesin subunits [10].

Condensin has also been implicated in chromosome organization and morphogenesis [16]. Condensin is a ring-shaped protein complex that mediates chromosome compaction and segregation during mitosis and meiosis [16, 26, 27]. Recent studies have demonstrated that condensins are involved in a wide range of chromosome-related functions and processes such as genome integrity, genetic recombination, epigenetic regulation, and differentiation [28–30]. Most eukaryotes have two types of condensin complexes, condensin I and condensin II. Their core subunits, SMC2 and SMC4, belong to a chromosomal ATPase family referred to as the SMC family, which is conserved in most eukaryotic species [31, 32]. Further, these complexes contain a unique set of non-SMC regulatory subunits, HEAT-repeat and kleisin subunits [16, 29, 31]. Previous studies have reported that retinoblastoma-associated protein (RB) is required to facilitate chromosome condensation by recruiting the condensin complex to the chromosomes [33, 34]. Thus, RB is generally thought of as a condensin modulator in the chromosome condensation process.

During meiosis, meiotic cohesin components, including α-kleisin REC8 (the human homolog of yeast Rec8) and its interacting subunits, play essential roles not only in sister chromatid cohesion but also genetic recombination and chromosome morphogenesis [9, 10, 34–40]. Mammalian REC8 physically interacts with STAG3, a meiosis-specific STAG protein, to maintain its stability and to associate with the axis of the meiotic chromosomes [41, 42]. REC8 forms a cohesin complex with SMC1β and SMC3, but not with SMC1α, and localizes to the axial element (AE) of the synaptonemal complex (SC) throughout meiotic prophase I [37]. In mice lacking REC8, both male and female meiocytes exhibit a failure of meiotic prophase I progression [35]. Further, REC8 prevents local AE separation and illegitimate SC formation between sister chromatids [43]. The partial loss of cohesin causes centromere decompaction and kinetochore fragmentation, particularly in aged oocytes [44, 45]. In the budding yeast *Saccharomyces cerevisiae*, cohesin associates with the chromosome axis during meiotic prophase and promotes SC formation, homolog template choice, and crossover-specific recombination [26, 38, 46, 47]. Furthermore, the meiotically expressed Scc1/Mcd1 (RAD21 in vertebrates) can partially substitute Rec8 to support chromosome axes and interhomolog partner interactions during recombination [46]. However, Rec8 can also carry out meiosis-specific activities through its interactions with meiosis-specific proteins [46].

These features imply that both mitotic and meiotic cohesins regulate chromosomal morphogenesis during prophase and support the accurate segregation of anaphase chromosomes. However, no previous studies have determined whether meiosis-specific cohesin components contribute to mitotic chromosome organization. The present study addresses unexpected phenomena related to the dynamic activity of meiosis-specific cohesin components, α-kleisin REC8 and STAG3, in mitotic ESC chromosomes. We first show that ESCs express high levels of both REC8 and STAG3. We then explored the possible roles of these meiotic components compared to their mitotic counterparts. Concretely, this study sought to determine whether these meiotic cohesion components play distinct and overlapping roles in the presence of mitotic cohesins in chromosome structure organization, sister cohesion, and DNA replication in ESCs. Our findings demonstrated that the REC8/STAG3-containing cohesin complex is functionally active in ESCs and that REC8-cohesin could compete with mitotic RAD21-cohesin for the structural organization of tightly conjoined sister chromosomes. High-resolution 3D imaging revealed that after knockdown of mitotic or meiotic cohesins, hyperloading of RB-condensin complexes on chromosomes induces the formation of shorter and thicker chromosomes. Our findings thus highlight the essential roles of meiosis-specific cohesin complexes in mitotic ESC chromosomes.

## Results

### Meiotic cohesin components REC8 and STAG3 are expressed in ESCs

Unlike differentiated cells, ESCs exhibit a globally open chromatin structure with various epigenetic landscapes [2, 4, 48, 49], which might explain why ESCs can express diverse genes involved in chromosomal morphogenesis. We therefore aimed to determine whether meiotic cohesin components could be expressed or function as mitotic cohesin components in mouse ESCs. We first analyzed the expression pattern of cohesin proteins in whole-cell lysates isolated from ESCs, mouse embryonic fibroblasts (MEFs), and testes from mice by western blotting. The mitotic cohesin components SMC3, SMC1α, STAG1, STAG2, and RAD21 were expressed in both ESCs and MEFs; remarkably, the meiosis-specific cohesin components REC8 and STAG3, except for RAD21L and SMC1β, were abundantly expressed in ESCs but not in MEFs (Fig. 1b). Given that mouse testis tubules include small fractions of testicular somatic cells, mitotic cohesin factors, SMC1α, RAD21, STAG1, and STAG2, were detectable in testis samples; however, the highest levels were present in MEFs and ESCs, where they exclusively participate in mitosis (Fig. 1b; Additional file 1: Fig. S1a). In addition, qPCR analysis confirmed that the expression patterns of meiotic cohesin components REC8 and STAG3 were similar to those determined via protein blot assays (Fig. 1c).

Next, co-immunoprecipitation (co-IP) assays were conducted to further determine whether the meiotic cohesin components were linked to the SMC ring and α-kleisin subunit, which connects the long coiled-coil arms of the ring, or formed an intact cohesin complex with mitotic components. Concretely, we analyzed whether SMC3 forms a complex with RAD21, REC8, STAG1, STAG2, and STAG3 using antibodies against RAD21, REC8, STAG1, STAG2, and STAG3. As shown for RAD21, REC8, and STAG3 also strongly interacted with SMC3 in ESCs but not in MEFs (Fig. 1d). These results indicated that REC8/STAG3-containing cohesin may have various functions in the mitotic program of ESCs.

Stem cells from the epiblast of mouse embryos can exhibit “naïve” and “primed” pluripotent states [50]. Mouse ESCs resemble the naïve pluripotent state that occurs in the early pre-implantation embryo, while epiblast stem cells (EpiSCs) derived from the late post-implantation epiblast constitute primed pluripotent cells that differ from naïve ESCs in the expression pattern of pluripotency-related genes [51–54]. To investigate whether meiotic cohesin could contribute to sister chromatid cohesion and chromosome morphogenesis in the primed states of pluripotency, we examined the expression of cohesin components in mouse EpiSCs. EpiSCs exhibited a high level of expression of mitotic cohesin factors comparable to that of MEFs or ESCs, whereas the expression levels of meiosis-specific cohesin factors REC8 and STAG3 were only marginally detectable in EpiSCs (Additional file 1: Fig. S1b). Additionally, to test whether ESC differentiation changes the expression kinetics of REC8 and STAG3, we differentiated ESCs by withdrawing leukemia inhibitory factor from the culture medium. Compared with the control ESCs, cells undergoing differentiation expressed significantly lower levels of REC8 and STAG3 (Additional file 1: Fig. S1c). Taken together, our findings indicate that REC8 and STAG3 are required in naïve ESCs but not in primed ESCs.

### REC8 and STAG3 are required to ensure faithful chromosome segregation in ESCs

We next aimed to determine whether the meiotic cohesin components REC8 and STAG3 mediate to form proper cohesion, similar to mitotic cohesins, in ESCs and MEFs. We depleted the mitotic cohesin component RAD21 and the meiotic cohesin components REC8 and STAG3 by treating the cells with pooled siRNAs against SMC3, RAD21, REC8, and STAG3 (siSMC3, siRAD21, siREC8, and siSTAG3). In ESCs and MEFs, the knockdown efficiency of the cohesin components using siRNA pool was more than 76% (Additional file 1: Fig. S2), and the decreased expression of individual cohesin component did not affect the expression levels of other cohesin components (Additional file 1: Fig. S2).

Since cohesin is implicated in sister chromatid cohesion, we analyzed siRNA knockdown cells for cohesion via fluorescence in situ hybridization (FISH) analysis of cells that had undergone DNA replication as defined by BrdU incorporation. Under siCtrl treatment, G2-phase cells showed Class I signals (two spots), which corresponded to the two sister chromatids. Upon knockdown of individual cohesin component, the incidence of abnormal sister chromatid separation [Class II (three spots) and III (four spots)] was significantly increased, ranging from 14 to 21% in ESCs lacking SMC3, RAD21, REC8, or STAG3 (Fig. 1e, g). In MEFs, RAD21 and SMC3 knockdown resulted in a higher rate of abnormal sister chromatid separation (~ 21%) than REC8 (~ 4%) and STAG3 (~ 3%) knockdown (Fig. 1f, h). Overall, REC8 and STAG3 knockdown led to significant defects in sister chromatid cohesion.

### STAG3 mediates translocation of REC8 into the nucleus

STAG3 mediates REC8 stabilization, and the REC8–STAG3 complex is required for diverse functions including sister chromatid cohesion, chromosome axis organization, and recombination during meiosis [55, 56]. To investigate whether the function of REC8 in ESCs is STAG3-dependent, REC8-STAG3 interactions were characterized using a protein immunoprecipitation assay. Consistent with the previous results [55, 56], REC8 physically interacted with STAG3 but not with STAG1 or STAG2 (Fig. 1i), suggesting that REC8 and STAG3 can cooperate to promote the formation of cohesin complexes in ESCs. To better understand the localization of REC8 and STAG3, we analyzed REC8 positive and negative signals in nuclei via immunofluorescence microscopy. REC8-staining intensities were significantly reduced by siSTAG3 (11.3%) compared to those in siCtrl nuclei (40.2%) (Fig. 1j). Ectopic expression of STAG3 resulted in greater REC8 levels in the nucleus, reaching a more than 1.46-fold upregulation compared to siCtrl (Fig. 1j). Furthermore, fractionation of cytosol and nuclear proteins from ESCs revealed that REC8 and STAG3 were present in both the nucleus and the cytosol. Upon STAG3 knockdown, the REC8 levels in the nucleus were reduced by ~ 27%. However, overexpression of STAG3 resulted in higher REC8 levels in the nucleus, accounting for a more than 1.3-fold increase compared to siCtrl (Fig. 1k, l). Thus, our findings suggest that STAG3 mediates REC8-cohesin stabilization and that REC8 is translocated into the nucleus in a STAG3-dependent manner (Fig. 1m) but did not affect the translocation of cohesin RAD21.

### REC8–STAG3 cohesin complexes play an important role in replication fork progression in ESCs

**Fig. 2:**
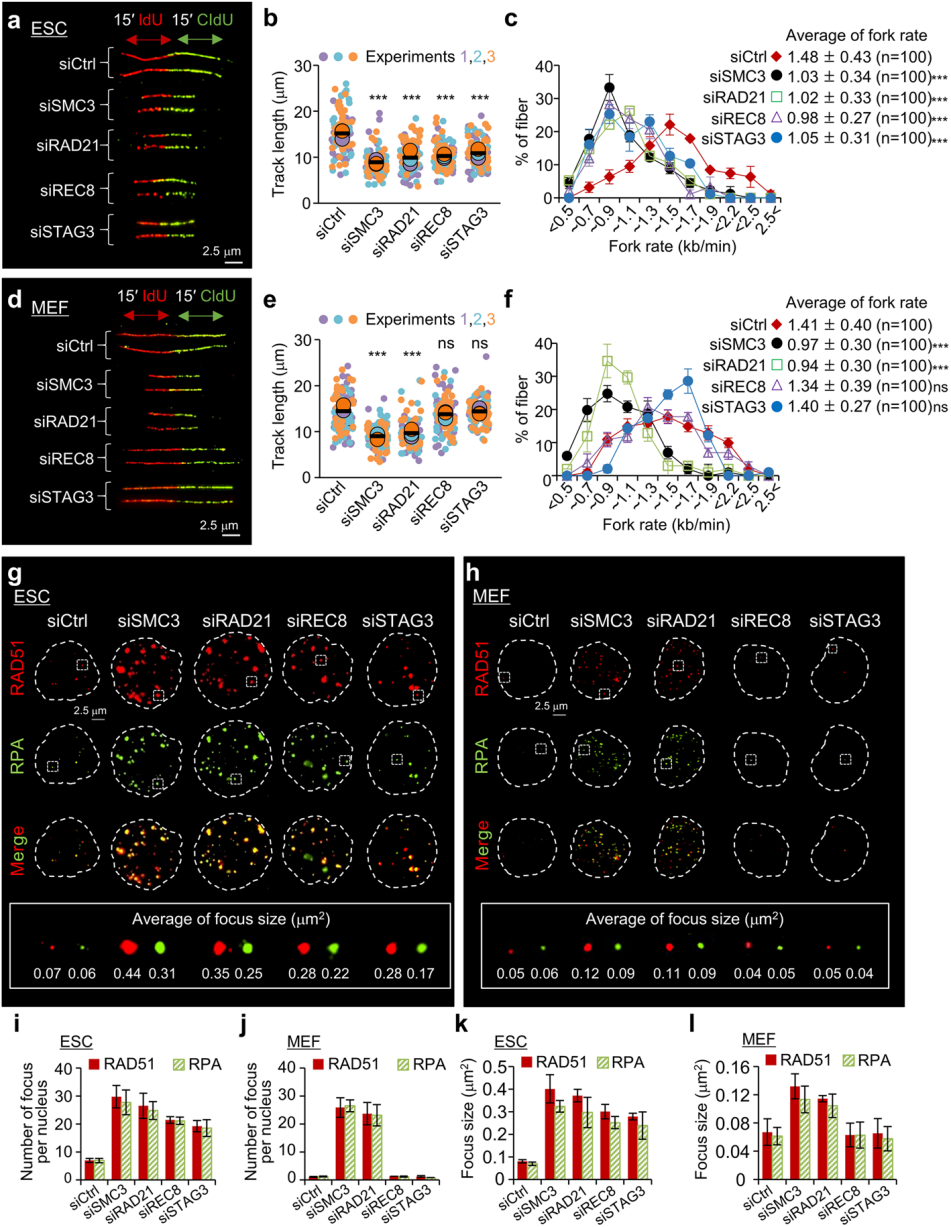
Role of meiotic cohesins in replication fork progression. **a,d** DNA fiber labeling scheme (Top): IdU is incorporated at the first analog (15 min), followed by CldU (15 min), which was incorporated as the second analog. Representative image of DNA fiber in ESCs and MEFs (bottom). Scale bars are 2.5 μm. **b,e** Quantification of length of IdU and CIdU tracks in siCtrl and siCohesin. The cells were transfected with siCtrl or siCohesin (siSMC3, siRAD21, siREC8, and siSTAG3) before DNA labeling, as indicated in the figure. Each biological replicate is color-coded (red, green, and blue) and the average of each replicated data is indicated with a larger dot, and black bars indicate the averages of three means (*n* ≥ 40 for condition). The error bars are the mean ± SD from three independent biological replicates. Statistics: Paired two-tailed *t*-test; ns, not significant; ***P < 0.001. **c**,**f** Quantification of elongation rates during DNA replication. The lengths of the DNA fibers (IdU and CldU tracks) were measured as shown in **a**. To calculate the DNA fork rates, the DNA fiber lengths were converted to kilobases (kb) (1 mm = 2.94 kb) and the converted values were divided by the CldU/IdU pulse labeling time (30 min). Statistics: Paired two-tailed *t*-test; ns, not significant; ***P < 0.001; *n*, the number of measured fibers. **g,h** Images of RAD51 and RPA foci formation in ESCs and MEFs (top). The focus size in each condition was analyzed using Nikon software (bottom). Scale bars = 2.5 μm. **i,j** Quantification of RAD51 and RPA foci. The numbers of focus were counted in siCtrl (control) cells and siCohesin (siSMC3, siRAD21, siREC8, and siSTAG3)-treated cells. The error bars are the mean ± SD from three independent experiments (*n* ≥ 50 for condition). **k,l** Average focus size after knockdown of cohesin components. Focus size was quantified in normal cells and cohesin knockdown cells. The error bars are the mean ± SD from three independent experiments (*n* ≥ 50 for condition)

Sister chromatid cohesion is established during DNA replication in both mitosis and meiosis [57, 58]. As cohesin components are enriched at the DNA replication fork and provide a bridge linking the sister chromatids [11, 58, 59], we explored whether the REC8–STAG3 cohesin complexes play a role in the DNA replication process. To analyze the molecular functions of REC8–STAG3 cohesin complexes in DNA replication, we consecutively pulse-labeled ESCs and MEFs with the nucleotide analogs CldU and ldU and investigated the DNA replication process in a single strand of DNA (Fig. 2a, d). The labeled elongating DNA strands were immunostained with antibodies to visualize the progression of the replication forks. The track length and fork progression rate were significantly reduced by more than 1.4-fold in SMC3-, RAD21-, REC8-, and STAG3-depleted ESCs compared with siCtrl ESCs (Fig. 2b, c), suggesting a delay in the progression of replication forks. In MEFs, the length of replicating DNA strands and the rate of DNA replication elongation after knockdown of cohesin components decreased by 1.45-fold and 1.48-fold with siSMC3 and siRAD21, respectively; however, we did not observe any significant difference after the knockdown of REC8 (1.04-fold) or STAG3 (1.01-fold) (Fig. 2e, f). We further analyzed the cell cycle and found that cohesin downregulation decelerated S-phase progression in ESCs (Additional file 1: Fig. S3).

Based on these data, we further hypothesized that the knockdown of REC8–STAG3 cohesin complexes might cause the accumulation of ssDNA gaps during the deceleration of S-phase progression. We further analyzed the formation of RAD51 and replication protein A (RPA) foci after knocking down the targeted cohesin components in ESCs and MEFs with siSMC3, siRAD21, siREC8, and siSTAG3. Knockdown of REC8 and STAG3 dramatically increased the number of RAD51 and RPA foci in ESCs but not in MEFs (Fig. 2g–j). Moreover, the size of the foci in ESCs was also considerably increased compared to that in MEFs (Fig. 2k, l). Thus, the lack of cohesion established by the REC8–STAG3 cohesin complexes could cause RAD51 and RPA foci accumulation at DNA gaps in ESCs. Further, our findings indicated that REC8 knockdown resulted in a significant increase in the proportion of apoptotic cells among ESCs (Additional file 1: Fig. S4), suggesting that REC8–cohesin and RAD21-cohesin jointly contribute to cohesion-mediated cellular progression.

### Knockdown of REC8 and STAG3 causes precocious sister chromatid separation and hypercompaction of chromosomes in ESCs

**Fig. 3:**
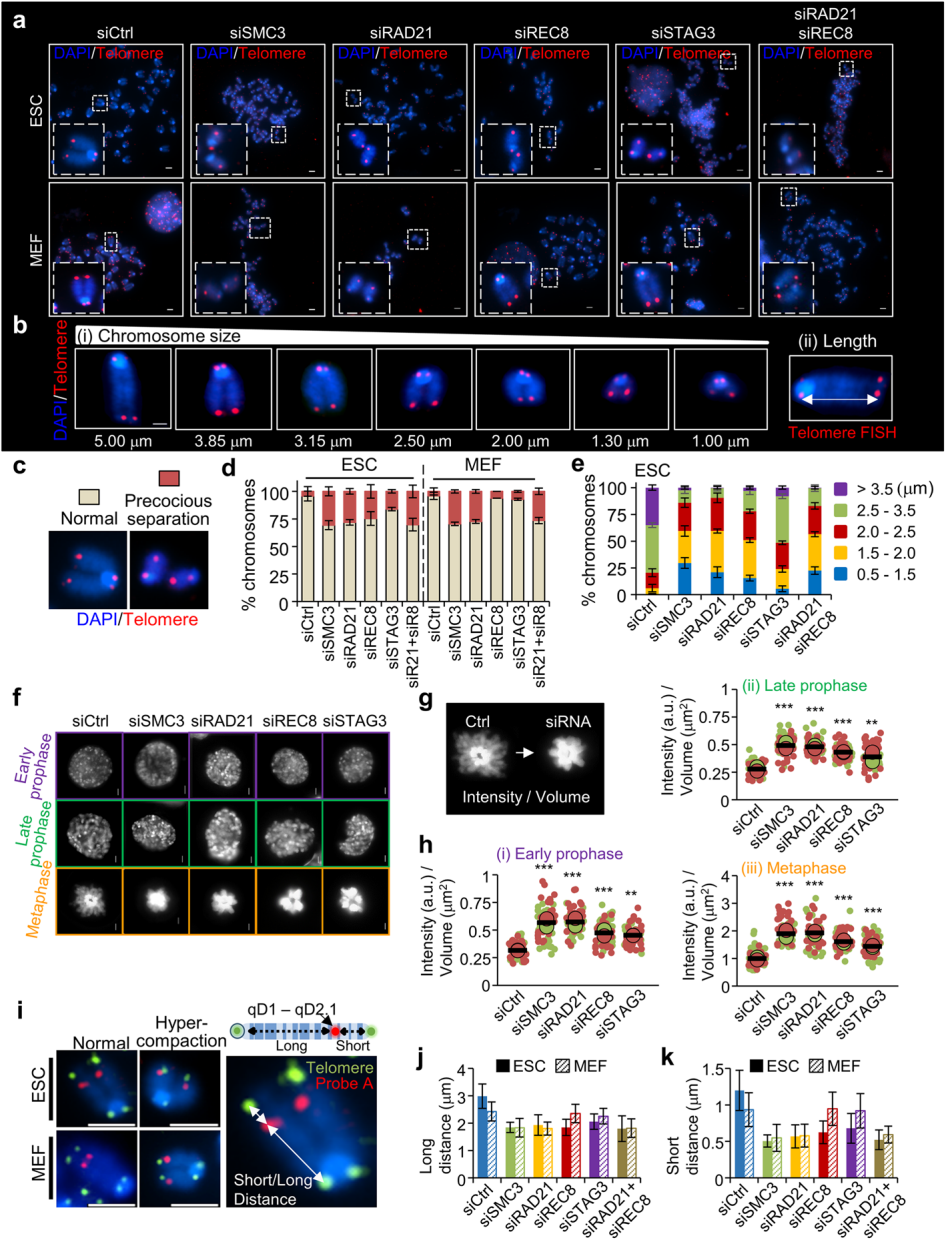
Chromosome compaction and precocious separation after the knockdown of cohesin components. **a** Metaphase chromosome images of telomere FISH from ESCs and MEFs expressing a non-targeting siRNA or different siRNAs against SMC3, RAD21, REC8, STAG3, and RAD21-REC8 (siSMC3, siRAD21, siREC8, siSTAG3, siRAD21-siREC8). Telomeric probe signals are shown in red and DAPI-counterstained chromosomes are shown in blue. Scale bars = 10 μm. **b** Metaphase chromosome images of diverse length. (i) Metaphase chromosomes stained with DAPI and telomere FISH. (ii) Measurement of chromosome length by telomere FISH. Scale bar = 2.5 μm. **c** Images of a metaphase spread from normal (left) and precocious separation (right) showing proper and defective chromosome separation, respectively. **d** Quantification of normal chromosomes and precocious separated chromosomes. The bar graph indicates the percentages of cells showing a ratio of normal chromosomes and precocious separated chromosomes among metaphase chromosomes (*n* = 200 per condition) from three biological replicates. The error bars represent the mean ± SD (n = 3). siR21, siRAD21; siR8, siREC8. **e** Quantification of chromosome length and percentage of average chromosome length per metaphase cells. Length of chromosomes was measured by calculating the distance between both sides of the telomere probes. Data are shown as mean ± SD value from at least 200 chromosomes per independent experiment (*n* = 3). **f** Compaction analysis of chromosomes from the prophase to the metaphase. ESCs expressing histone H2B-GPF were analyzed by fluorescence microscopy. Scale bars = 2.5 μm. **g** Representative images of ESCs marked with H2B-GFP in metaphase. **h** Quantification of chromosome compaction. Cell volume and intensity were analyzed using the Nikon NIS software. The *X*- and *Y*-axes of the graphs indicate intensity and volume (μm^2^), respectively. Each biological replicate is color-coded (red and green) and the average of each replicated data is indicated with a larger dot, and black bars indicate the averages of three means (*n* = 50 for condition). The error bars are the mean ± SD from two biological replicates. **P < 0.01, ***P < 0.001. **i** Metaphase spreads hybridized with the telomeric probe. The locus-specific probe (“Probe A”) binds to the region of Ch 4 (Chromosome 4: 116,094,264–116,123,690). Telomeric probe and locus-specific probe signals are shown in green and red, respectively. The lengths of chromosomes were measured by calculating the distance between the telomere and locus-specific probes. The chromosome lengths were analyzed using Nikon NIS software. Scale bars are 2.5 μm. **j,k** Quantification of chromosome lengths in metaphase chromosomes from cells treated or untreated with siRNA specific to the cohesin components for 24 h (*n* = 30 for condition). The error bars represent the mean ± SD from three independent experiments

Cohesins play a role in sister chromatid cohesion and thus ensure faithful chromosome segregation during mitosis and meiosis [11, 20, 21, 60–63]. To investigate whether the REC8–STAG3 cohesin complexes play a role in the maintenance of sister chromatid cohesion in ESCs, FISH assays were conducted with a telomere DNA probe to analyze chromosome cohesion in metaphase chromosome spreads. Knockdown of cohesin components in ESCs resulted in increased rates of precocious sister chromatid separation (4.20% with siCtrl, 30.6% with siSMC3, 28.03% with siRAD21, 24.64% with siREC8, 15.98% with siSTAG3, and 30.16% with siRAD21 -siREC8) (Fig. 3a–d). In MEFs, the rate of precocious centromere separation was significantly increased by siSMC3 (29.5%) or siRAD21 (27.28%) but not by siREC8 (5.15%) or siSTAG3 (6.12%) compared to that in siCtrl cells (4.02%) (Fig. 3a–d).

To evaluate changes in length distributions, we classified the chromosome length into five numerical ranges and also described the length by tracking the distance between the telomeres along each sister chromatid in different sister chromatid pairs. In ESCs lacking SMC3, RAD21, REC8, STAG3, and RAD21-REC8, the proportion of chromosomes with lengths in the 0.5–2.0 μm range were approximately 61%, 60%, 51%, 25%, and 55% higher than those of siCtrl cells, respectively (Fig. 3b, e). In contrast, the chromosome length distributions in MEFs did not change significantly except after the knockdown of SMC3 and RAD21 (Additional file 1: Fig. S5). Thus, when meiotic/mitotic cohesin components were depleted, chromosome hypercompaction occurred in ESCs (Additional file 1: Fig. S5), which also suggests that the REC8–STAG3 cohesin complexes have additional roles in concert with RAD21-containing mitotic cohesin complexes in ESCs.

Previous studies have suggested that cohesin dissociation from chromosome arms contributes to chromosome condensation during mitosis [11, 64, 65]. However, the mechanisms by which chromosome compaction is regulated in normal or aberrant sister chromatid cohesion remain unknown. Therefore, to understand the role of cohesin in chromosome morphogenesis in ESCs, we next examined its effects on chromosome volume and intensity from early prophase to metaphase by stably expressing GFP-labeled histone H2B (H2B-GFP) in these cells. The chromosomes were more dramatically compacted in cells treated with siRNA targeting cohesin components than in siCtrl cells, becoming both denser and thicker from early prophase to metaphase (Fig. 3f). Additionally, the intensity/volume values were increased after siSMC3, siRAD21, siREC8, and siSTAG3 treatment compared to siCtrl treatment (Fig. 3f–h).

To measure chromosome compaction more precisely, FISH analysis was performed using two probes to mark telomeres and a locus in the arm of chromosome 4 (chromosome 4: 116,094,264–116,123,690), after which the distance between each telomere and the specific locus in the arm was determined. In ESCs lacking SMC3, RAD21, REC8, STAG3, and RAD21-REC8, the long and short distances between each telomere and the locus in the arm were reduced by more than 1.1 μm and 0.5 μm, respectively (Fig. 3i–k). In contrast, the chromosome distance in MEFs was not significantly changed except after RAD21 or SMC3 knockdown (Fig. 3i–k). Taken together, our findings indicate that the loss of cohesin components induced irregular chromosome compaction during the metaphase stage.

Previous studies have shown that cohesin and its regulators contribute to the maintenance of ESCs in an undifferentiated state [9, 66, 67]. Moreover, our findings indicated that SMC3-, RAD21-, REC8-, and STAG3-depleted cells expressed lower levels of Oct4 (a pluripotency marker) compared with control ESCs (Additional file 1: Fig. S2a). Further, alkaline phosphatase (AP) staining showed a reduction in AP staining-positive cells for cohesin depletion, suggesting the loss of pluripotency upon cohesin knockdown (Additional file 1: Fig. S6). Thus, the chromosome structural changes observed in cohesin-depleted ESCs might have resulted from ESC pluripotency loss. However, under normal conditions, both ESCs and MEFs exhibit similar chromosome structural changes throughout the cell cycle process. Furthermore, chromosome structural changes such as chromosome hypercompaction, which occur after cohesin depletion in ESCs, show the same tendency as in MEF. Therefore, a decrease in the expression of pluripotency marker caused by cohesin depletion would not have a direct effect on chromosomal structure changes.

### Elevated expression of RB promotes chromosome hypercompaction

**Fig. 4:**
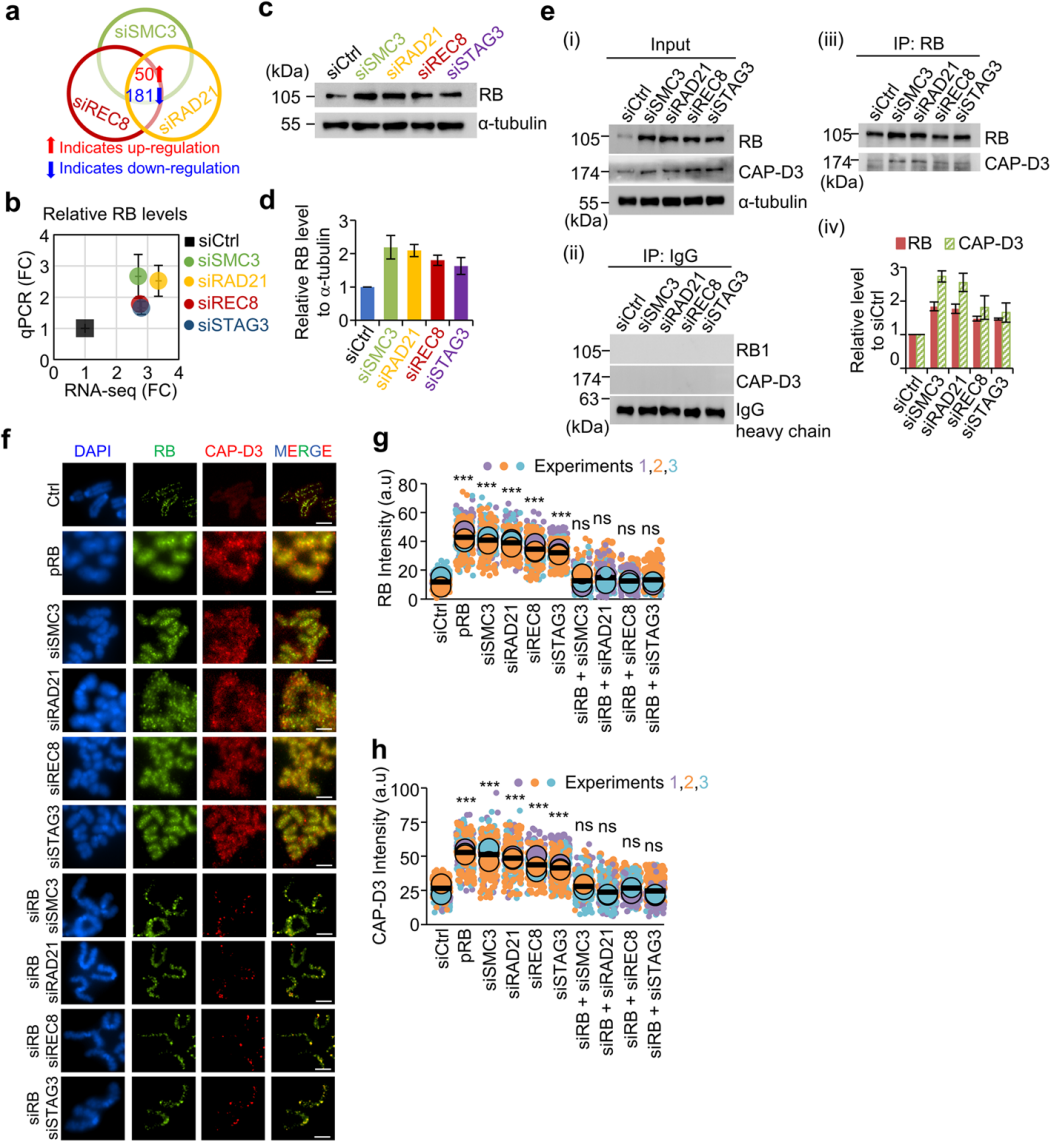
Chromosome shortening during the metaphase upon the knockdown of cohesin components. **a** Venn diagram for identification of up- or downregulated genes from siRNA-mediated knockdown of SMC3, RAD21, and REC8 in ESCs. Data were adjusted with *P* < 0.1 and fold-change > 1.5. (Additional file 2: Table S1). **b** Comparison of gene expression of RB. RB transcript levels from qPCR and RNA sequencing experiments were analyzed to evaluate RB gene expression. The error bars are the mean ± SD (*n* = 3 for qPCR). The RB expression levels measured by RNA-Seq experiments are represented as average values of two biological replicates. **c** Analysis of RB protein levels following transfection with a siRNA pool against SMC3, REC8, RAD21, and STAG3 in ESCs. α-tubulin was used as a loading control. **d** Quantification of RB expression shown in **c** using Image Lab 6.0 software (Bio-Rad). Results are illustrated as mean ± SD value (*n* = 3). **e** Immunoprecipitation (IP) analysis for physical interaction between RB and CAP-D3 in ESCs. (i–iii) Western blotting; (iv) quantification of RB and CAP-D3 levels from IP results. The error bars are the mean ± SD from three independent biological replicates. **f** Immunofluorescence analysis of chromosome compaction in ESCs during the metaphase stage. Chromosomes were stained with anti-RB antibody, anti-CAP-D3, and DAPI. Ctrl, siControl; pRB, pEF1α-RB expression vector; siSMC3, siRAD21, siREC8, STAG3, and siCohesin/siRB1 indicate siRNA treatment against SMC3, RAD21, REC8, STAG3, and cohesin/RB1 respectively. **g,h** Intensity of RB and CAP-D3 protein signals per a chromosome in ESCs. a.u., arbitrary unit. The data are reported as mean ± SD values from three biological replicates (*n* > 100 for condition). Statistics: Paired two-tailed *t*-test; ns, not significant; ***P < 0.001

To investigate the global changes in gene expression in response to aberrant sister chromatid cohesion, which can be used to identify potential targets for chromosome hypercompaction, we performed RNA sequencing analysis following treatment with siSMC3, siRAD21, and siREC8. Venn diagram analysis revealed that 50 genes were more highly expressed in cohesin knockdown cells (Fig. 4a; Additional file 2: Table S1). Among the 50 differentially upregulated genes, the RB, a binding partner of condensin, was significantly upregulated more than 2.7-fold after depletion of cohesin components (Fig. 4b; Additional file 2: Table S1). Further, gene sets derived from qPCR and western blotting were used to investigate whether cohesin-knockdown ESCs exhibited changes in the expression of RB. Interestingly, the expression levels of RB were significantly increased in response to SMC3, RAD21, REC8, and STAG3 knockdown (Fig. 4b–d). Moreover, through immunoprecipitation of RB followed by western blotting of condensin II complex subunit D3 (CAP-D3; a condensin component), we confirmed that high RB expression levels strongly increased RB-condensin interaction after cohesin knockdown (Fig. 4e). To characterize the effects of these high RB levels, localization of RB and chromosome compaction were analyzed after transfection with siRNA (siSMC3, siREC8, siSTAG3, and siRAD21) and an RB expression plasmid containing EF1α promoter (pEF1α -RB, pRB). RBs and CAP-D3 were highly localized to entire chromosomes in the metaphase and the chromosomes were more dramatically compacted in cells transfected with the pEF1α-RB plasmid and siRNA targeting cohesin components than in siCtrl cells (Fig. 4f). Knockdown of cohesin components in ESCs resulted in increased levels of RB- and CAP-D3 signals in metaphase chromosomes (3.4-fold and 1.8-fold in siSMC3, 3.4-fold and 1.7-fold in siRAD21, 2.72-fold and 1.56-fold in siSTAG3, and 3.1-fold and 1.7-fold in siREC8) (Fig. 4g, h). Furthermore, when RBs were ectopically expressed, the staining intensity of RB and CAP-D3 on chromosomes was increased by 3.9-fold and 1.8-fold, respectively, and the chromosomes were dramatically compacted (Fig. 4f–h). These results suggest that depletion of cohesin confers elevated expression of RB and that the abundance of RB-condensin complexes actively promotes chromosome hypercompaction. Additionally, we confirmed that double knockdown of RB and cohesin factors rescued chromosome hypercompaction, whereas sister chromatids remained separated (Fig. 4f–h).

### REC8 and mitotic cohesin RAD21 are located predominantly at nonoverlapping sites on ESC chromosomes

**Fig. 5:**
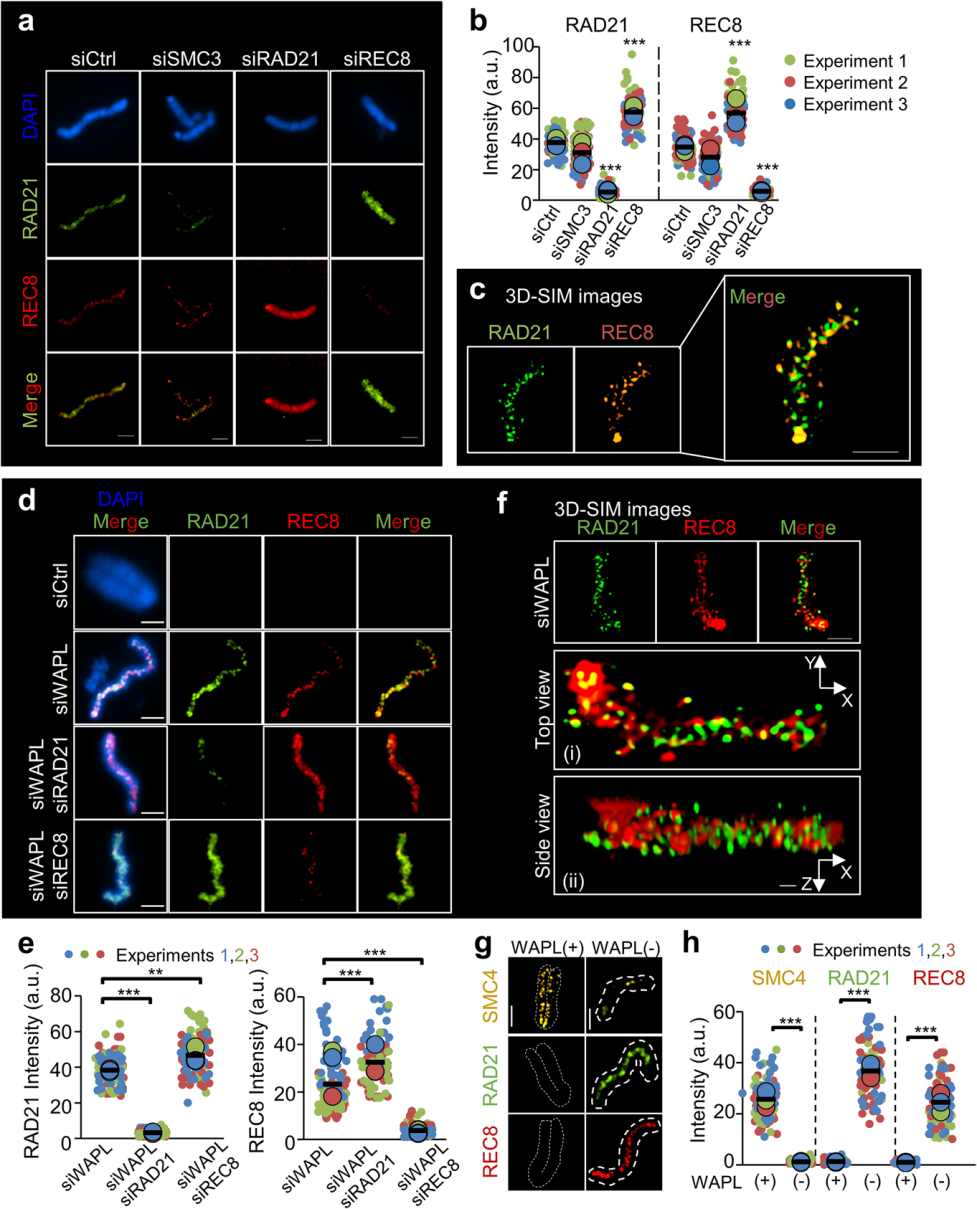
Chromosome association of mitotic and meiotic cohesin components. **a** Representative images of prometaphase-like chromosomes in cohesin knockdown. ESC chromosomes synchronized with thymidine were stained with REC8 or RAD21 antibodies and DAPI. Scale bars = 2.5 μm. **b** Quantification of the intensity level of RAD21 and REC8 in ESC chromosomes. Each biological replicate is color-coded (red, green, and blue) and the average of each replicated data is indicated with a larger dot, and black bars indicate the averages of three means (*n* = 50 for condition). The error bars are the mean ± SD from three independent biological replicates. ns: not significant, ***P < 0.001. **c** 3D structured illumination microscopy (SIM) images of RAD21 and REC8 in ESC chromosomes. **d** Representative images of prometaphase-like chromosomes in WAPL-knockdown ESCs. The synchronized cells were treated with siCtrl (Control, metaphase chromosome; first row) or siRNA specific to WAPL (second row), WAPL/RAD21 (third row), and WAPL/REC8 (fourth row). Chromosome spreads were stained them with REC8 or RAD21 antibodies and DAPI. **e** Quantification of cohesin intensity shown in **d**. Results are illustrated as mean ± SD value (*n* = 50 per condition). *P*-values (paired two-tailed t-test) were calculated using the GraphPad Prism 5 software: **P < 0.01, ***P < 0.001. **f** 3D SIM images of WAPL-knockdown cells. The fixed chromosomes were immunostained with REC8 and RAD21 antibodies. Scale bar = 2.5 μm. 3D SIM images were analyzed using NIS software from Nikon. Scale bar = 10 μm (Side view). **g** Fluorescence microscopy images of prometaphase-like chromosomes in WAPL-knockdown cells. Fixed chromosomes were immunostained with antibodies against RAD21, REC8, and SMC4. Scale bars = 2.5 μm. **h** Quantification of cohesin and condensin intensity. The error bars are the mean ± SD from three biological replicates (*n* ≥ 30 for condition). ***P < 0.001

Cohesins present at the interface of sister chromatids are linked by DNA/structural bridges in the prophase stage [68, 69]; both REC8 and RAD21 might be independently localized between sister chromatids [70]. Microscopic analysis of REC8 and RAD21 was then conducted to investigate the binding pattern specificity of these molecules on prometaphase-like chromosomes of ESC. RAD21 and REC8 were located at adjacent sites but predominantly at nonoverlapping sites on chromosome arms (Fig. 5a). We then measured the cohesin intensity following the depletion of RAD21 or REC8. When RAD21 was depleted, the intensity of REC8 on chromosomes increased more than 2.03-fold compared with that of normal cells (Fig. 5a, b; right panel). Additionally, our findings demonstrated that RAD21 intensity increased more than 1.34-fold in REC8-depleted cells compared with normal cells (Fig. 5b; left panel), suggesting that RAD21 may compete with REC8 to form cohesin complexes. Additionally, the binding regions of RAD21 and REC8 were more precisely analyzed via high-resolution 3D SIM imaging. The two proteins were found to localize at discrete sites along the chromosome axis and the adjacent regions, but they rarely overlapped (Fig. 5c).

WAPL promotes the dissociation of cohesin complexes from chromosomes by providing a DNA exit gate during the prophase and metaphase [23, 71]. We therefore suppressed the expression of WAPL, a cohesin release factor, and/or REC8 and RAD21 using siRNA and then investigated the localization of mitotic RAD21 cohesin and meiotic REC8 cohesin on mitotic prometaphase-like chromosomes via immunofluorescence microscopy. Our findings indicated that RAD21 and REC8 were highly accumulated on closed chromosomes after WAPL knockdown (Fig. 5d–f). The rate of closed chromosome morphology was higher in cells with WAPL knockdown (74.9%) than in siCtrl cells (10.8%) (Additional file 1: Fig. S7), indicating that chromosome arm-bound cohesins were stably maintained [23, 71]. Consistent with the results of normal ESCs (Fig. 5a–c), the intensity of REC8 on the chromosomes of ESCs in which both RAD21 and WAPL were depleted increased more than 1.4-fold compared with that in ESCs with WAPL knockdown (Fig. 5d–f). Furthermore, RAD21 intensity increased more than 1.3-fold in WAPL/REC8-depleted cells compared with WAPL-knockdown cells. Given that WAPL knockdown resulted in localization of RAD21 and REC8 on the chromosomes, we next aimed to identify the localization of condensin, which is normally located on sister chromosomes from the prophase to the anaphase stage [29]. As SMC4 is a core component of condensins, we immunostained cells with anti-SMC4 antibodies to observe the localization of SMC4 on chromosomes. Interestingly, after the loss of WAPL, the intensity of SMC4 was reduced by more than 22-fold, whereas the intensities of RAD21 and REC8 increased by 31-fold and 21-fold, respectively (Fig. 5g, h).

### Changes in chromosome structure and SMC4 localization patterns after knockdown of REC8, STAG3, RAD21, and SMC3

**Fig. 6:**
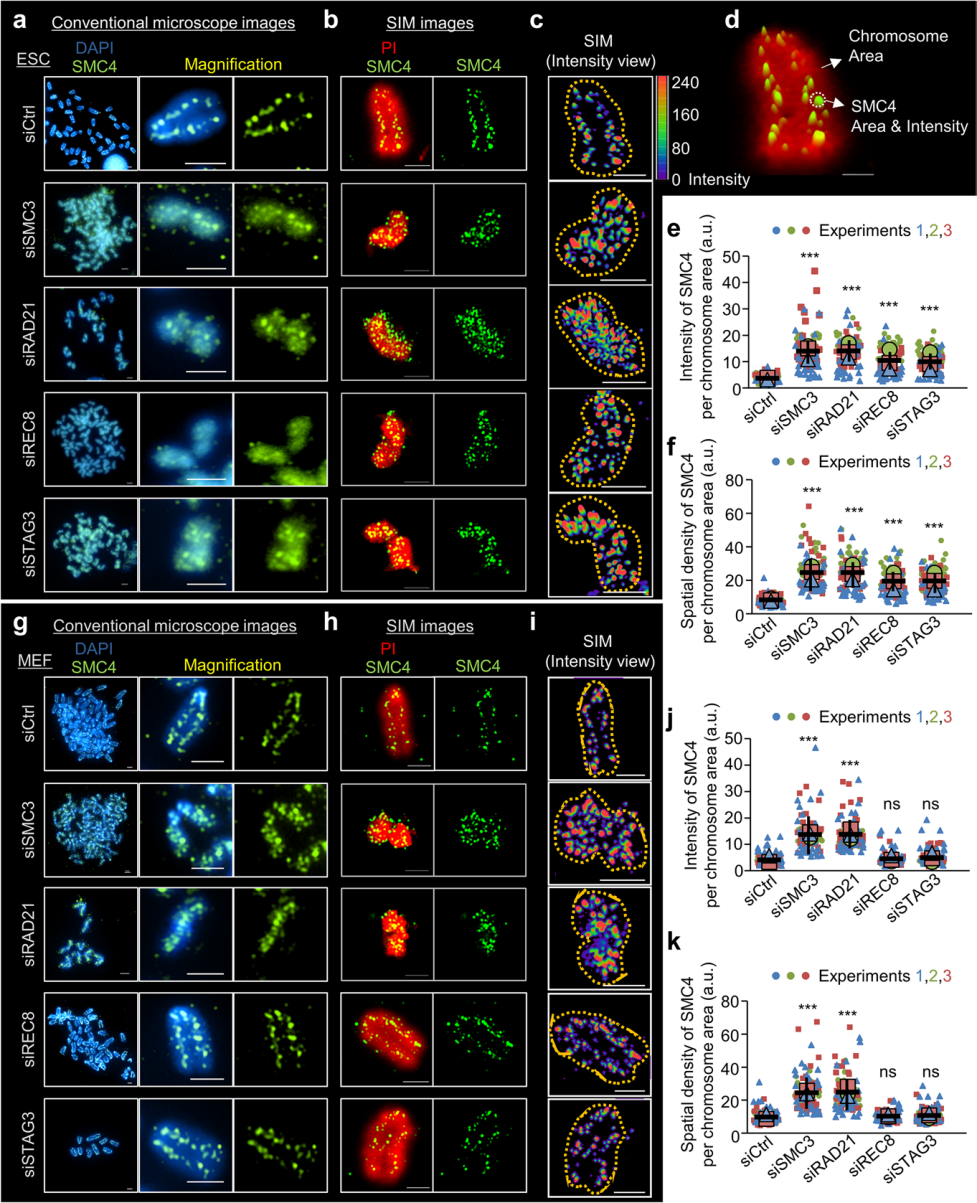
Changes in chromosome structures and localization patterns of condensin after knockdown of cohesin components. **a,g** Fluorescence images of metaphase chromosomes. The chromosomes were immunostained with antibody against SMC4 and DAPI (left) in ESCs and MEFs. Scale bar = 2.5 μm. **b**,**h** 3D SIM images of metaphase chromosomes. The cells were stained with anti-SMC4 antibody and PI. Scale bar = 2.5 μm. **c,i** 3D-rendering images of SMC4-stained metaphase chromosomes. SMC4 is pseudo-colored in each color to measure the localization and intensity of condensin on metaphase chromosomes. 3D-rendered images were created using the Nikon NIS software. Scale bar = 2.5 μm. **d** Intensity and distribution of SMC4 in metaphase chromosomes. **e,f,j,k** The intensity and spatial density of SMC4 per chromosome area in metaphase ESCs (**e,f**) and MEFs (**j,k**). The *y*-axis values represent the intensity of SMC4 and the spatial density of SMC4 per a chromosome area. The *y*-axis values were calculated by dividing the volume of chromosome area by the intensity or volume of SMC4 spots. The area and intensity were measured using Nikon NIS software. Each biological replicate is color-coded (red, green, and blue) and the average of each replicated data is indicated with a larger dot, and black bars indicate the averages of three means (*n* = 30 for condition). The error bars are the mean ± SD from three independent biological replicates. Statistics: Paired two-tailed *t*-test, ns: not significant, *P < 0.5, **P < 0.01, and ***P < 0.001

Cohesin complexes are released from chromosome arms during prophase [11, 72] to allow their localization to the mitotic chromosome axis and linkages between different DNA loops, resulting in overall chromosome axis compaction [13, 16, 73, 74]. We therefore expected that cohesin knockdown would lead to global condensin localization to chromosomes from the prophase to the metaphase and cause dramatic changes in chromosome compaction. To determine the distribution of condensin after knockdown of REC8, STAG3, RAD21, and SMC3, synchronized cells were observed in the metaphase, after which SMC4 was observed via fluorescence microscopy. In ESCs lacking cohesin, chromosomes were more compacted than those in siCtrl cells and exhibited greater condensin distribution along the whole chromosome, whereas condensin in siCtrl cells was normally located on the mitotic chromosome axis (Fig. 6a). Additionally, in cells with knockdown of SMC3, RAD21, REC8, or STAG3, SMC4 proteins were distributed more extensively along mitotic chromosomes and showed stronger signals, as illustrated by the 3D projections of the chromosomes (Fig. 6b, c). However, knockdown of REC8 or STAG3 did not affect the distribution or intensity of SMC4 in MEFs (Fig. 6g–i). In cohesin-knockdown cells, we observed numerous SMC4 puncta in compacted chromosomes, indicating that mitotic/meiotic cohesin knockdown specifically induced the recruitment of condensin complexes.

To better understand the effects of condensin distribution on cohesin knockdown, the intensity of SMC4 and its spatial density per chromosome unit area were quantified using high-resolution 3D SIM imaging (Fig. 6d). The intensity and spatial density of SMC4 after the knockdown of cohesin components were increased by more than 3.7-fold (intensity) and 1.94-fold (density) in both ESCs and MEFs. In contrast, compared with siCtrl, siREC8 and siSTAG3 had no significant effect in MEFs (Fig. 6e, f; Fig. 6j, k).

To determine the cell cycle phase during which chromosomal localization of SMC4 was induced, we next analyzed the intensity of SMC4 during the interphase, prophase, and metaphase in ESCs and MEFs. During the interphase in ESCs and MEFs, the intensity of SMC4 did not substantially differ between cohesin-knockdown cells and siCtrl cells, suggesting that SMC4 can initially localize to chromosomes after the interphase (Additional file 1: Fig. S8a). In contrast, during the prophase and metaphase in ESCs, condensins were distributed in a large area of the chromosomes and accumulated more extensively near the centromeres of cohesin-knockdown cells compared with siCtrl cells (Additional file 1: Figs. S8b and c; top panels). Knockdown of REC8 and STAG3 did not affect the intensity of SMC4 in MEFs during the prophase and metaphase (Additional file 1: Figs. S8b and c; bottom panels). Collectively, our data indicate that knockdown of meiotic cohesin induces an irregular distribution of condensins on chromosomes in ESCs, leading to chromosome hypercompaction and that meiotic cohesins also contribute to the morphogenesis of mitotic chromosomes in ESCs (Additional file 1: Fig. S9).

### Chromosomal loading of SMC4 after knockdown of REC8 and STAG3

**Fig. 7:**
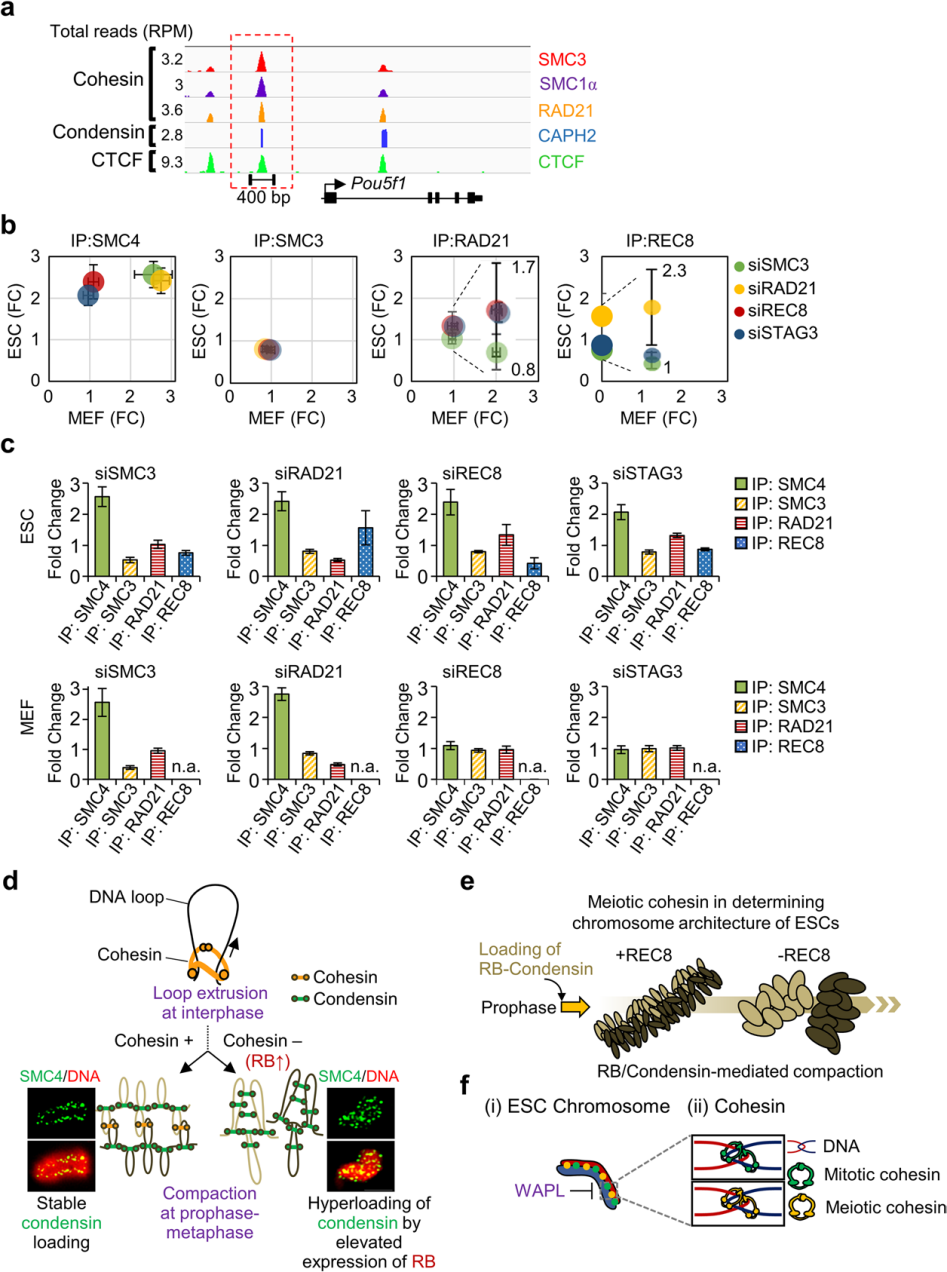
Genome-wide occupancy of cohesin and condensin in ESCs. **a** ChIP-Seq binding profiles for cohesin components (SMC3, SMC1α, and RAD21), condensin component (CAPH2), and CCCTC-binding factor (CTCF) at the chr.4 35,365,101–35,365,484 loci in ESCs [9, 75]. ChIP-Seq data are reported as reads per million with the *y*-axis floor set to 0.2 reads per million. Previously published ChIP-seq datasets from ESCs were reanalyzed in the encyclopedia of DNA elements [9, 75]. Scale bars are depicted above the profiles. **b** Comparison of observed condensin and cohesin binding in mitotic ESC and MEF chromosomes. The data show the relative level of DNA bound in siCtrl (Control) and siCohesin cells after pulling-down each cohesin component (SMC4, SMC3, RAD21, and REC8) at the cohesin/condensin binding regions [75]. A scatter plot was generated to visualize the normalized fold-change values in ESCs and MEFs. The error bars are the mean ± SD from three biological replicates. FC, fold-change value. **c** Condensin and cohesin ChIP analyses using siRNA for mitotic chromosomes in ESCs and MEF. The fold-change values were normalized for each condition to the value of siCtrl samples. Bar graphs indicated average of each replicated fold-change values. The error bars the mean ± SD from three biological replicates. n.a.: not applicable. **d** Regulation of chromosome structure by cohesin and condensin. **e** Proposed model of chromosome morphogenesis in normal or aberrant meiotic cohesin complexes in ESCs. Loss of REC8-containing cohesin induces high RB expression level and enhances the compaction of chromosome structures from the prophase to the metaphase. **f** Cohesin loading onto chromosomes. (i) The WAPL-mediated removal of meiotic and mitotic cohesins recruits RB-condensin complexes and promotes ESC chromosome condensation. In the absence of WAPL, cohesin removal is suppressed and condensin cannot be efficiently loaded on the chromosomes. (ii) Distinct localization of mitotic and meiotic cohesins at cohesion sites in ESCs

A previous study demonstrated that the binding sites of cohesin complexes overlapped with those of condensin complexes and that they cooccupied the core promoter regions of the ESC pluripotency gene *Pou5f1* (Fig. 7a) [75]. Therefore, we speculated that the knockdown of cohesin could cause condensin complex recruitment to cohesin binding sites. To gain insight into the proportion of the genome occupied by cohesin and condensin complexes in ESCs and MEFs, ChIP analysis was performed with REC8, RAD21, STAG3, SMC3, and SMC4 for mitotic chromosomes. Interestingly, the level of SMC4 bound at these sites was increased in cohesin component-deficient ESCs compared to siCtrl ESCs by more than 2.24-fold (siSMC3), 2.21-fold (siRAD21), 1.60-fold (siREC8), and 1.58-fold (siSTAG3) (Fig. 7b, c). In MEFs, the level of bound SMC4 under cohesin-knockdown conditions was increased by more than 2.89-fold (siSMC3) and 2.41-fold (siRAD21), but knockdown of meiotic cohesin (siREC8: 0.94-fold; siSTAG3: 1.02-fold) did not affect the proportion of bound SMC4 (Fig. 7b, c). On the other hand, the levels of SMC3, REC8, and RAD21 bound at these binding sites did not change significantly in ESCs and MEFs (Fig. 7b, c). Thus, these results indicate that SMC4 proteins are abundantly loaded at cohesin binding sites after knockdown of cohesin components. These data suggest that the release of meiotic or mitotic cohesin might enable condensins to bind to cohesin binding sites in ESC chromosomes.

## Discussion

We first studied how meiotic cohesins cooperate with mitotic cohesins to regulate sister chromatid cohesion and investigated their roles in chromosome organization, DNA replication, and repair during mitosis in ESCs. The meiotic cohesin components REC8 and STAG3 were specifically expressed in ESCs and were sufficient to function as essential cohesin components in ESCs. The relative contributions of the meiosis-specific cohesin to chromosome structure and cellular functions in mitotic programs had remained largely uncharacterized. This study has led to several new findings which provide deeper insights into the mechanisms that control the morphogenesis of chromosomes and sister chromatid cohesion, which relies on a delicate balance between the mitotic and meiotic cohesin complexes in mitotic ESC chromosomes.

ESCs simultaneously expressed both REC8 and STAG3, and these proteins were copurified from nuclear fractions. REC8-STAG3 containing cohesin plays a critical role in regulating sister chromatid cohesion and chromosome topology in ESCs. Höög and colleagues reported that the α-kleisin REC8 in meiocytes exhibited dosage-dependent STAG3 requirements and that STAG3-REC8 cohesin complexes have an important role in supporting sister chromatid cohesion and the meiotic chromosome axis [41]. Our findings indicated that ectopic expression of STAG3 resulted in higher levels of REC8 in the nucleus, accounting for a more than 1.5-fold increase compared to siCtrl. Therefore, high levels of STAG3 are required for REC8-containing cohesin stabilization and accumulation onto the ESC chromosomes. This new finding suggests that ESCs systematically express STAG3 to regulate the translocation of REC8 into the nucleus. Therefore, REC8/STAG3-containing cohesin complexes in the nucleus could function with mitotic cohesin complexes and specifically mediate ESC-specific cohesin roles. These findings build upon those of a previous study that revealed that ectopically expressed-REC8 in Hek293 cells requires STAG3 to functionally replace RAD21, the mitotic counterpart of REC8 [76].

It has been previously shown that centromeric cohesion is critical for precise chromosome segregation and is likely the reason for cohesin accumulation at centromeres during mitosis [17]. During the metaphase-to-anaphase transition, when all chromosomes exhibit bioriented mitotic spindles, the cysteine protease separase (ESPL1/ESP1) cleaves the centromeric RAD21 protein to finally separate the sister chromatids [38, 76–80]. In our study, both REC8- and RAD21-cohesin complexes were required to hold sister chromatids at centromeres before the onset of the anaphase stage. REC8-knockdown ESCs were found to have major defects in mitotic chromosome cohesion but were nevertheless capable of forming the metaphase plate with precociously separated sister chromatids. Thus, to suppress the precocious separation of sister chromatids, REC8-cohesin complexes might have additional roles to stabilize the centromeric cohesion of ESCs. Since the separase effectively recognizes phosphorylated REC8 [39, 81, 82], a kinase or its regulatory factor may also participate in REC8 modification to control cohesin levels on ESC chromosomes.

Chromosomes have been shown to undergo global morphological changes between the compacted and expanded stages [83]. Mitotic chromosomes are compacted in preparation for the chromosome segregation that occurs during the preanaphase stage and maintain a parallel alignment of sister chromatids [64, 84]. The compaction-dependent organization of chromosomes progresses from the prophase to the metaphase through cycles of stress accumulation and release [64]. A recent study using high-resolution 3D imaging suggested that cohesin-containing “mini-axis” bridges link the split sister axes of mitotic chromosomes to provide mechanical stability during the mitotic prophase [68] (Fig. 7d). Collectively, these findings suggest that mechanical stress within the chromosome axes might modulate whole-chromosome morphology. Cohesin and condensin could be involved in the regulation of chromosome compaction during the prophase in both mitosis and meiosis [38, 85, 86]. It has been shown in yeast meiosis that the absence of cohesin components induces the longitudinal hypercompaction of meiotic chromosomes during the prophase [38]. Condensins are localized along the central axes of the mitotic chromosomes to allow linkage between adjacent and/or distant DNA loops during the prophase-to-metaphase transition (Fig. 7d). Condensin I and II differentially occupy chromosome regions and cooperate in chromosome assembly from prophase to metaphase [27, 28]. Condensin II associates with chromatin simultaneously with cohesin release and participates in chromosome condensation in the early prophase. On the other hand, condensin I is involved in chromosome condensation after nuclear envelope break down at the late prophase. Thus, RB-induced condensin II loading on ESC chromosomes might be the key process that mediates faithful chromosome morphogenesis during the early stage of chromosome compaction [32]. In the interphase, DNA loops are formed, extruded, and tethered together by cohesin [87]. We speculate that high levels of RB-condensin complexes bind widely to chromosomes, form new loops, and induce long-range looping by tethering adjacent loops in the absence of cohesin complexes [88]. As the cells progress into the metaphase stage, the absence of REC8- or RAD21-cohesin complexes causes precocious separation of sister chromatids and hyperloading of RB-condensin complexes on mitotic chromosomes, resulting in chromosome hypercompaction (Fig. 7d, e). Mitotic and meiotic cohesin complexes are loaded onto ESC chromosomes during the interphase, which is followed by cohesin dissociation and condensin loading in the early prophase. However, the absence of the REC8-cohesin complexes results in a wide distribution of condensin in mitotic chromosomes and creates short and thick chromosomes. This suggests that the meiotic REC8/STAG3-cohesin complex is essential to modulate ESC chromosome morphogenesis, even in the presence of RAD21-cohesin complexes (Fig. 7e, f). Another study suggests that chromosome loop size increases while axis length decreases progressively during the compaction process [89]. General DNA loop extrusion during the interphase is regulated by cohesins, but recent studies have suggested that condensin complexes can contribute to the same mechanism [16, 90]. However, we could not detect condensin proteins in ESCs or MEFs during the interphase, and the loss of cohesins showed that the condensins were randomly distributed in the chromosome bodies during the prophase to metaphase transition. Additionally, the pattern observed during the prophase was similar in fixed whole nuclei and in chromosome spread samples, thus further demonstrating that the condensins were bound all over the chromosome after cohesin knockdown, resulting in irregularly shaped compaction. Thus, a timely association and/or dissociation of condensin and cohesin is important to promote normal chromosome compaction during mitosis in ESCs.

RNA sequencing datasets with knockdown of cohesin components (SMC3, REC8, RAD21, and STAG3) demonstrated that RB transcripts were significantly upregulated in cohesin-knockdown ESCs compared with the expression levels in control cells. Surprisingly, ectopic overexpression of RB-induced hypercompaction of chromosomes during the metaphase, as demonstrated by the knockdown of cohesin components. Furthermore, RB physically interacts with condensin II, which mediates mitotic chromosome condensation ([31]; this study). These observations imply that high RB expression levels enhance condensin hyperloading on chromosomes, resulting in chromosome hypercompaction. We thus suggest that cohesin-mediated remodeling signals regulate chromosome compaction as a consequence of molecular redistribution. These findings offer a new framework for the characterization of mitotic/meiotic cohesin- and condensin-mediated chromosome compaction.

During the cell cycle, cohesins bound to interphase chromatin are released during the prophase, which coincides with their association with condensin [87, 91]. Generally, condensins participate in the mitotic phase of chromosome assembly in the early prophase and promote chromosome compaction in preparation for mitotic chromosome segregation [28, 29]. However, the mechanism by which condensins achieve the enormous task of timely compaction of the entire genome remains largely uncharacterized. Chromosome compaction also requires cohesins, which support the close alignment of sister chromatids at an earlier point than condensin [11, 32, 86]. Thus, our results suggested that both cohesin and condensin are involved in the modulation of chromosome structure and play similar roles in ensuring timely compaction. It has been previously shown that cohesin and condensin overlap at the same binding sites in the genome [9, 75]. These observations allowed us to propose a model for the co-occupation of cohesin/condensin binding sites. Concretely, condensin can gain greater access to chromosomes and attach to cohesin binding sites by co-occupying these binding sites when cohesin components are absent (Fig. 7d–f). Our co-IP analysis indicated that the level of condensin bound to cohesin binding sites increased when several mitotic or meiotic cohesin components were depleted in ESCs. Further, hyperloading of RB-condensin can be replaced with cohesins and are widely/irregularly distributed along prophase and metaphase chromosomes after cohesin knockdown. This indicates that the RB-condensin complex might regulate the chromosome structure by directly binding to cohesin binding sites. However, given that cohesin binding is restricted to open chromatin sites, the possibility that condensin regulates chromosome compaction by binding at cohesin binding sites as well as other binding sites cannot be ruled out.

Chromosomes undergo precise topological changes during diverse phases of the cell cycle. These processes must be executed with precision to avoid genome instability and whole-chromosome aneuploidy, which can cause tumorigenesis, cell death, and congenital disorders [45, 92, 93]. In addition to its role in sister chromatid cohesion, cohesin plays an important role in diverse endogenous and exogenous stresses [94, 95]. Cohesin accumulates at DNA double-strand break (DSB) sites and is required for proper DNA repair, which relies on proper sister chromatid cohesion [17, 96, 97]. Previous studies have demonstrated that the absence of Rec8 in budding yeast leads to a considerable decrease in the rate of recombinant product formations, suggesting its role in programmed DSB repair processes through recombination during meiosis [38, 46, 95, 98]. Although the roles of REC8 in the regulation of chromosome segregation and genetic recombination have been reported during meiosis [46], there has been no definitive study on its role during the mitotic cell cycle. Here, we found that knockdown of the REC8 or RAD21 resulted in a significant increase in the proportion of apoptotic cells and a delay in DNA replication in ESCs. Thus, both REC8-cohesin and RAD21-cohesin contribute to faithful DNA replication and participate in DNA repair pathways during mitosis. However, given that RB is a negative regulator of S-phase progression, accumulation of RB upon knockdown of cohesin components might inhibit the cell cycle progression of ESCs [99, 100]. We thus could not determine whether REC8-cohesin and RAD21-cohesin directly regulate replication fork progression or indirectly by upregulating RB. Nevertheless, considering the observations, it is reasonable to claim that the cohesin complexes composed of REC8 and RAD21 are functionally linked to cell cycle regulation by playing an essential role in the maintenance of chromosome structural stability and cohesion-mediated cell cycle progression of ESCs.

## Conclusion

Based on the findings outlined above, we propose that REC8 and STAG3 are key components of the mitotic cohesin complex in ESCs. In addition to its general role in the organization of chromosome topological properties as seen in the RAD21-cohesin complexes of ESCs, REC8-cohesin complexes are essential for sister chromatid cohesion. Furthermore, high-resolution 3D imaging revealed that hyperloading of RB-condensin complexes on chromosomes induces chromosome hypercompaction after REC8- or RAD21-cohesin knockdown. These new insights demonstrate how mitotic and meiotic cohesins promote distinct chromosome interactions and that chromosome morphogenesis is regulated in the presence or absence of mitotic and meiotic cohesin factors. Our findings thus provide novel insights into the process of chromatid cohesion and chromosome topology in the mitotic ESC chromosomes.

## Methods

### Cell lines

Murine ESCs (J1) and MEFs were prepared as described previously [5]. ESCs were cultured on 0.1% gelatin-coated 60-mm dishes in Dulbecco’s modified Eagle’s medium (DMEM; 10569, Gibco) supplemented with 10% horse serum (#16050122, Gibco), 10 mM HEPES (#15630080, Gibco), 2 mM L-glutamine (#25030081, Gibco), 0.1 mM minimum essential medium non-essential amino acids (#11140050, Gibco), 0.1 mM β-mercaptoethanol (#21985023, Gibco), 100 U/ml penicillin-streptomycin (#15140122, Gibco), and 10^3^ U/mL ESGRO recombinant mouse leukemia inhibitory factor (LIF) (ESG1107, Millipore) in a humidified cell incubator with 5% CO_2_ at 37 °C. Mouse epiblast stem cells (E3 EpiSCs) were established in the presence of IWR1 [51, 101]. EpiSCs were maintained on a dish layered with mitomycin C-treated MEF cells in region-selective EpiSC (rsEpiSC) medium [N2B27 supplemented with 20 ng/ml of basic fibroblast growth factor (bFGF), 2.5 μM of IWR1, and 0.1% bovine serum albumin (BSA)], and the medium was exchanged daily. EpiSCs were passaged every 2 to 3 days by dissociation into clumps with Accutase (Stemcell Technologies, Canada).

### Antibodies

The following primary antibodies were used in this study: anti-rabbit SMC3 (Abcam; ab9263; 1:5,000), anti-rabbit SMC1α (ab9262, Abcam; 1:5,000), anti-rabbit SMC1β (ab96206, Abcam; 1:2,000), anti-rabbit RAD21 (ab154769, Abcam; 1:5,000), anti-rabbit RAD21L (kindly provided from Dr. Jibak Lee and Dr. Alberto M Pendás), anti-goat STAG1 (ab4457, Abcam; 1:5,000), anti-mouse STAG2 (sc-81852, Santa Cruz; 1:3,000), anti-rabbit STAG3 (ab185109, Abcam; 1:1,000), anti-rabbit REC8 (ab192241, Abcam; 1:3,000), anti-rabbit CAP-D3 (ab70349, Abcam; 1:2,000), anti-rabbit SMC4 (ab17958, Abcam; 1:5,000), anti-rabbit α-tubulin (ab4074, Abcam; 1:10,000), anti-mouse β-Actin (ab8224, Abcam; 1:10,000), anti-mouse IdU (B44, BD; 1:25), anti-rat BrdU (ab6326, Abcam; 1:500), anti-mouse OCT3/4 (sc-5297, Santa Cruz; 1:3,000), anti-rat RPA (#2208, Cell signaling; 1:3,000), anti-mouse RAD51 (PC130, Merck Millipore; 1:3,000), anti-rabbit RB (ab181616, Abcam; 1:2000), and anti-mouse RB (ab24, Abcam; 1:2000). The following secondary antibodies were then used: Peroxidase AffiniPure goat anti-mouse IgG (115-035-003, Jackson Immunoresearch; 1:10,000), Peroxidase AffiniPure goat anti-rabbit IgG (111-035-003, Jackson Immunoresearch; 1:10,000), Peroxidase AffiniPure donkey anti-goat IgG (705-035-003, Jackson Immunoresearch; 10,000), anti-mouse Alexa 488 (111-545-003, Jackson Immunoresearch; 1:500), anti-rat fluorescein isothiocyanate (FITC) (112- 095-003, Jackson Immunoresearch; 1:500), anti-rabbit TRITC (115-025-003, Jackson Immunoresearch; 1:500), and anti-rabbit Cy3 (111-165-003, Jackson Immunoresearch; 1:500). We have produced a rabbit polyclonal antibody against the recombinant C-terminal 342 amino acid sequences of REC8 (GenBank accession numbers: mouse REC8, BC052155.1).

### Immunoprecipitation

Cell lysates were prepared as described previously [5]. For immunoprecipitation analysis, the cells were cultured to a final concentration of 5 × 10^6^ cells per 60-mm culture dish. The cells were washed with PBS to remove the extra medium and treated 0.5% Trypsin-EDTA for 3 min at 37 °C. Afterward, the cells were then resuspended with PBS and centrifuged them at 1000 rpm for 3 min. Cell pellets were resuspended with protein lysis buffer (50 mM Tris-HCl pH 7.6, 250 mM NaCl, 1 mM DTT, 5 mM MgCl_2_, 0.05% NP40, and 0.1 mM EDTA) for 10 min on ice. After centrifugation (13,500 rpm for 10 min), the protein lysates were transferred to microtubes and protein concentrations were determined using the Bradford assay. Cell lysates (700 μg) were incubated with 1 μg of primary antibodies overnight and mixed with 20 μl 50% protein A/G agarose beads for 3 h at 4 °C. The samples were then centrifuged at 3000 rpm for 3 min at 4 °C and the supernatants were discarded. Protein A/G agarose beads were washed three times with washing buffer (50 mM HEPES, 2.5 mM MgCl_2,_ 100 mM KCl, 10% glycerol, 1 mM DTT, and 0.1% NP40 with PIC) and PBS. After centrifugation (3000 rpm for 3 min at 4 °C), agarose beads were mixed with 20 μl 2× Laemmli SDS sample buffer and boiled for 5 min at 100 °C. The protein samples were then transferred to fresh microtubes and analyzed via gel electrophoresis.

### Cell cycle synchronization and inhibitor treatments

For imaging of ESC and MEF chromosomes, cells were synchronized at G1-S phase by incubating cells with 2 mM thymidine (Cat. 89270, Sigma) for 16 h, after which the inhibitor was washed away with DMEM. Afterward, the cells were treated with 2 mM thymidine and incubated for 16 h. The samples were then washed with PBS and cultured in fresh growth medium to release the cells from the thymidine block. To obtain synchronized cells, duplicated cultures were collected after thymidine block release at 1-h intervals. Cells enter mitosis at ~ 6 h after release.

### Spread chromosome preparation

Whole cells were hypotonically swelled with a hypotonic buffer (75 mM KCl, 0.8% Na Citrate, and H_2_O_2_ 1:1:1 v/v/v) for 10 min at 37 °C. Three volumes of cold methanol-acetic acid (3:1) were then added to the hypotonic buffer and the mixture was allowed to sit for 3 min. The supernatant was then removed with an aspirator and 5 ml of cold methanol-acetic acid (3:1) was added to the cell pellets. The cell pellets were then incubated for 5 min, after which they were gently resuspended and a drop of cell suspension was placed on a slide. As soon as the surface of the slide was dry, the slides were rehydrated by dipping them in PBS-azide buffer (10 mM NaPO_4_ pH 7.4, 0.15 M NaCl, 1 mM EGTA, and 0.01% NaN_3_) for 10 min. The slides were then swollen by washing them three times (3 min each) with TEEN buffer (1 mM Tris-HCl pH 8.0, 0.2 mM EDTA, 25 mM NaCl, 0.5% Triton X-100, and 0.1% BSA). Primary antibodies were then added on the slide for 1 h at room temperature. To remove the primary antibodies (diluted in TEEN), the samples were washed three times with KB washing buffer (10 mM Tris-HCl pH 8.0, 0.15 M NaCl, and 0.1% BSA) for 3 min. Fluorescein-conjugated secondary antibodies (diluted in TEEN) were then incubated on the slide for 1 h at room temperature. After a final wash with KB buffer, the slides were mounted with a DAPI-containing antifade solution.

### Immunostaining

Cells were cultured on poly-L-lysine-coated (sc-286689, Santa Cruz) 12-mm coverslips (#0111520, Deckglaser) and then fixed with 4% paraformaldehyde for 10 min. The cells were then permeabilized with 0.2% Triton X-100 for 5 min and washed with PBS-T (0.1% Tween 20 in PBS) for 3 min three times. Cells were blocked with 3% BSA in PBS-T for 30 min and then incubated for 1 h with the following primary antibodies (diluted in 1% BSA in PBS-T): anti-RPA (#2208, Cell Signaling; 1:200) and anti-Rad51 (PC130, Merck Millipore; 1:200). The cells were then washed three times with T-PBS, and cells were incubated for 1 h with the appropriate anti-Fluorescein isothiocyanate (FITC) (112-095-003, Jackson Immunoresearch; 1:500) or anti-Cy3 (111-165-003, Jackson Immunoresearch; 1:500) - conjugated secondary antibodies and then mounted with an antifade solution containing DAPI. Images were captured using the N-SIM S super resolution microscope (Nikon, Tokyo, Japan) and the Eclipse Ti-E fluorescence microscope (Nikon, Tokyo, Japan) equipped with fluorescence filters for DAPI and the indicated fluorophores conjugated to the secondary antibodies.

### Probes for fluorescence in situ hybridization

Telomere probes were prepared with the mouse repeat sequence (TTAGGG)_n_ and labeled with 5′-FAM and 3′-FAM primers (5′–6FAM-TTAGGGTTAGGGTTAGGGTTAGGGTTAGGG–3′ and 5′–6FAM-CCCTAACCCTAACCCTAACCCTAACCCTAA–3′) via a PCR reaction. PCR was performed in 50 μl reaction volumes containing 10 mM Tris-HCl (pH 8.3), 50 mM KCl, 1.5 mM MgCl_2_, 200 mM of each dNTP, 0.2 mM of each primer, and 2 units of Taq polymerase. The amplification program consisted of 10 cycles of 1 min at 95 °C, 30 s at 54 °C, and 1 min at 72 °C, followed by 30 cycles of 1 min at 95 °C, 30 s at 62 °C, 90 s at 72 °C, and one final step at 72 °C for 7 min. The locus-specific probe was bound to the RAD54 locus region (Chromosome 4: 116,094,264 – 116,123,690). Probes were labeled with Cy3-dCTP in the same PCR reaction. The PCR was carried out with mouse cDNA using the following primers: 5′-AGC ACA GTG GCG GCC GCA TGA GGA GGA GCT TA-3′ and 5′-TAG ACT CGA GCG GCC GCT CAG TGA AGC CGC GC-3′. Bacterial artificial chromosome (BAC) DNA was extracted with the NucleoBond Xtra Midi kit according to the manufacturer’s instructions. The purified BAC DNA was then labeled by standard nick translation reaction, including dATPs, dGTPs, dTTPs, Cy3-labeled dCTPs, translation buffer, diluted DNase I, DNA polymerase I, and BAC DNA. The mixture was incubated at 15 °C for 2 h. To inactivate the reaction, a stop solution (0.5 M EDTA and 10% SDS) was added and the mixture was incubated at 68 °C for 15 min. The products were then visualized on a 1.2% agarose gel to confirm the presence of a smear between 500 and 200 bp. The probes were stored at − 20 °C.

### Fluorescence in situ hybridization on metaphase chromosomes

For the FISH assays, the cells were incubated with 0.1 μg colcemid (#15212012, Gibco) for 3 h in a humidified incubator with 5% CO_2_ at 37 °C. The cells were resuspended in 75 mM KCl for 10 min at 37 °C, after which the fixed cell pellets were resuspended in methanol/acetic acid (3:1 v/v) and a drop of this suspension was placed on glass slides. These slides were allowed to sit undisturbed overnight at room temperature. The chromosome preparation was rehydrated in 2× SCC buffer, digested with pepsin (1 mg/ml) in 10 mM HCl (pH 2.0) for 10 min at 37 °C, fixed in 3.7% formaldehyde in PBS for 5 min, and then washed twice with PBS (5 min per wash). The samples were then rehydrated and serially incubated in 70%, 90%, and 100% cold ethanol and allowed to air dry. The slides were incubated with a pre-warmed (75 °C) denaturation buffer for 2 min on the area of the slide marked with good metaphase chromosomes. For the hybridization step, both slides and the Cy3-labeled probe were pre-warmed at 37 °C for 5 min, after which 8 μl of hybridization mixture was placed on the spotted area of the slice for 1 h at 37 °C. The slides were washed once in PBS, once in 2× SCC buffer at room temperature for 10 min, and twice in PBS (5 min per wash). The slides were then mounted with an antifade solution containing 4′,6-diamidino-2-phenylindole-dihydrochloride (1 μg/ml DAPI). To distinguish the cell cycle phases, bromodeoxyuridine (BrdU) (B5002, Sigma Corp.) was added to the growth medium and cells were cultured in a humidified incubator with 5% CO_2_ at 37 °C for 15 min. Then, the cells were harvested for the FISH analysis. The lengths of chromosomes corresponding to telomere- and locus-specific labeling were measured using the Nikon NIS software.

### Chromatin immunoprecipitation

For the chromatin immunoprecipitation (ChIP) assay, 4 × 10^5^–5 × 10^5^ ESCs and MEFs were cultured. The cells were chemically fixed with 1% formaldehyde for 15 min at room temperature, followed by glycine (125 mM) for an additional 5 min at room temperature. Afterward, the cells were lysed with SDS lysis buffer (50 mM HEPES, 140 mM NaCl, 1 mM EDTA, 1% Triton X-100, 0.1% sodium deoxycholate, 0.1% SDS, and 1× protease inhibitor) and solubilized by sonication. The cell lysates were incubated overnight at 4 °C with 1 μg of antibodies, after which 20 μl of protein A/G agarose beads were added (P9203-050, GenDEPOT). For the ChIP analysis of SMC3, RAD21, REC8, and SMC4 in MEFs and ESCs, protein A/G agarose beads were washed four times with a low-salt buffer (0.1% SDS, 1% Triton X-100, 2 mM EDTA, 20 mM Tris-HCl pH 8.0, and 150 mM NaCl), a high salt buffer (0.1% SDS, 1% Triton X-100, 2 mM EDTA, 20 mM Tris-HCl pH 8.0, and 500 mM NaCl), a LiCl buffer (0.25 M LiCl, 1% NP-40, 1% Na_2_ Deoxycholate, 1 mM EDTA, and 10 mM Tris-HCl pH 8.0), and a TE buffer (10 mM Tris pH 8.0, and 1 mM EDTA). The bound complexes were eluted from the beads with an elution buffer and reverse-crosslinked via incubation at 65 °C for 12 h. Purified DNA was quantified via qPCR using SYBR Green (K-6251, Bioneer) and the CFX Connect Real-Time PCR system (#1855201, Bio-Rad). We confirmed a subset of strongly bound cohesin sites overlapping with the binding sites of condensin [93]. qPCR was then conducted using a specific primer pair (forward: 5′–GTGCTGGGATTAAAGGCG–3′; reverse: 5′–AGATGAGGCTTTCAGGAAATAC–3′).

### Reverse transcription quantitative PCR analysis

To quantify the expression levels of cohesin and RB in ESCs and MEFs, total RNA samples were extracted, and cDNA synthesis was performed using SuperiorScript II reverse transcriptase (Enzynomics, Korea). qPCR analysis was performed with a SYBR Green (K-6251, Bioneer) and the CFX Connect Real-Time PCR system (#1855201, Bio-Rad). The PCR primers used for RT-qPCR analysis were as follows: SMC3-F (5′–TTACAAGATGAGCTGGCGGG–3′), SMC3-R (5′–TGTAGCTTGAGCCAACCTCG–3′), SMC1α-F (5′–ATTGGACCCAATGGCTCTGG–3′), SMC1α-R (5′–CTCCATGTATCAGGTCCCGC–3′), SMC1β-F (5′–AGCTACAACTGTGTGGCTCC–3′), SMC1β-R (5′–AAGAATGGAGCAGGCCGAAA–3′), RAD21-F (5′ –GAGGCTACACCGCACAAGG–3′), RAD21-R (5′–ATAAGAAGAAACCTGGATCTCGGC–3′), REC8-F (5′ –TTCAACAGTGCCAGTACCTTG–3′), REC8-R (5′–CTCTAAAAGGTGTCGAATCTGAGG–3′), RAD21L-F (5′–GGGAGAGCCAAATTCCCACA–3′), RAD21L-R (5′–TCCAAGAATATCCTGCTCAGAGT–3′), STAG1-F (5′ –GTTCAGGATGTCGAGGTACGG–3′), STAG1-R (5′–GGGGTAGTCACCACTGTCCT–3′), STAG2-F (5′ –GGGAACAACATTCATGTGACGG–3′), STAG2-R (5′–TCCTCTAAGAAAGACAGTTCCGAAG–3′), STAG3-F (5′ –TGTTCAGGATGTCGAGGTACG–3′), STAG3-R (5′–GGACCAGGCATTGTAAGGGG–3′), RB-F (5′–ATCCCTTGCATGGCTTTCAGATT–3′), RB-R (5′–GCTGAGAGGACAAGCAGGTT–3′), GAPDH-F (5′ –AAGGTCATCCCAGAGCTGAA–3′), and GAPDH-R (5′–CTGCTTCACCACCTTCTTGA–3′). Gene expression levels of each gene were quantified using the ΔCt method.

### DNA fiber assay

Cells (2 × 10^5^) were pulsed with 50 μM ldU (GP1769, Glentham) for 15 min and then sequentially pulsed with 100 μM CldU (C6891, Sigma) for 15 min. The cells were then resuspended in cold PBS at a concentration of 400 cells/μl. Next, 9 μl of DNA lysis buffer (200 mM Tris-HCl pH 7.4, 0.5% SDS, and 50 mM EDTA) was added to the samples and a drop of resuspended cells was placed on a slide. The slides were tilted approximately 10° to allow the lysed DNA to spread and let it dry for 30 min. The cells were fixed with methanol-acetic acid (3:1 v/v) for 10 min and dried overnight at room temperature. After washing with PBS, the slides were immersed in 2.5 N HCl for 60 min at room temperature to denature the DNA molecules. IdU incorporation was detected using a mouse anti-IdU antibody (B44, BD; 1:25) and BrdU incorporation was detected using a rat anti-BrdU antibody (ab6326, Abcam; 1:500). Slides were then washed with PBS and incubated with fluorescence secondary antibodies, TRITC-conjugated goat anti-mouse IgG antibody (115-025-003, Jackson Immunoresearch; 1:300), and FITC-labelled goat anti-rat IgG antibody (112-095-003, Jackson Immunoresearch; 1:400) to detect IdU and CldU, respectively. After a final wash with PBS, the slides were mounted with antifade solution. DNA fiber images were captured using a fluorescence microscope (Nikon Eclipse, Ti-E, Japan), and their lengths were measured based on IdU and CldU labeling using the Nikon NIS software.

### RNA interference

A commercially available predesigned siRNA (AccuTarget^TM^; Bioneer) specific to the target genes was used to knock down their endogenous expression in ESCs and MEFs. The siRNA pool included the following single targeting sequences:

siSMC3: 5′–CAGAUGAAGUCAGCACGAA(dTdT)–3′, siRAD21: 5′–GAGCUAGUGAUAACUCACU(dTdT)–3′, siREC8: 5′–GAGAUCAGUCGAGGAGACU(dTdT)–3′, siSTAG3: 5′–CUGGAUUAACAUGCCUACU(dTdT)–3′, siRB: 5′-CAGUUGAUCUAGAUGAGAU(dTdT)–3′, and siWAPL: 5′–GUCCUUGAAGAUAUACCAA(dTdT)–3′

Oligonucleotides were transfected using Lipofectamine RNAiMAX (#13778150, Thermo Fisher) according to the manufacturer’s instructions. A negative control siRNA purchased from Bioneer (SN-1003) was also used. The siRNAs were incubated with the cells in an optimum (serum-free) medium for 24 h.

### RNA-Seq library preparation and sequencing

Total RNA samples were isolated using the RNeasy Mini Kit (Qiagen) according to the manufacturer’s instructions. RNA quality was assessed using an Agilent 2100 bioanalyzer (Agilent Technologies, Amstelveen, Netherlands), and RNA concentration was determined using an ND-2000 spectrophotometer (Thermo Inc., USA). mRNA-Seq libraries were prepared from total RNA using the NEBNext Ultra II Directional RNA-Seq Kit (New England BioLabs, UK). mRNA was isolated using the Poly(A) RNA Selection Kit (Lexogen, Austria). The isolated mRNAs were used for cDNA synthesis and shearing following the manufacturer’s instructions. Indexing was performed using the Illumina indexes 1–12. The enrichment step was conducted via PCR. Afterward, the mean fragment size of the libraries was determined using the Agilent 2100 bioanalyzer (DNA High Sensitivity Kit). Quantification was performed using the library quantification kit using a StepOne Real-Time PCR System (Life Technologies, Inc., USA). High-throughput paired-end 100 sequencing was performed using a HiSeq X10 sequencer (Illumina, Inc., USA).

### Data analysis

The raw sequencing data were quality checked using FastQC. Adapter and low-quality reads (< Q20) were removed using the FASTX_Trimmer and BBMap tools. The trimmed reads were then mapped to the reference genome using TopHat. Gene expression levels were estimated as FPKM (fragments per kb of transcript per million reads) values using Cufflinks. The FPKM values were normalized based on the quantile normalization method using edgeR in R. Data mining and graphic visualization were performed using the ExDEGA software (E-biogen, Inc., Korea).

### Statistical analysis

All data were analyzed with the GraphPad Prism 5 and OriginPro 2020b software and reported as means ± SD. Statistically significant differences (*p < 0.05, **p < 0.01, and ***p < 0.001) between various groups were determined via paired two-tailed *t*-test. For Fig. 1g, h, and j, three biological replicates were performed (*n* ≥ 50 nuclei per condition). *P*-values (paired two-tailed *t*-test) were calculated using GraphPad Prism 5 software. For Fig. 2c and f, the DNA fiber lengths were converted to kilobases (kb) (1 mm = 2.94 kb) to calculate the DNA fork rates and the converted values were divided by the CldU/IdU pulse labeling time (40 min). *P*-values were obtained via the paired two-tailed *t*-test (ns: not significant, ***P < 0.001; *n*, number of measured fibers). For Fig. 4g and h, the intensities and RB and CAPD-3 were quantified using Nikon NIS software. The error bars represent the mean ± SD values of three independent experiments (at least 30 chromosomes were counted). *P*-values (paired two-tailed t-test) were calculated using the GraphPad Prism 5 software. For Fig. 7b, the fold-change values were normalized for each condition to the value of the siCtrl cell samples. A scatter plot was then generated to visualize the normalized fold-change values in ESCs and MEFs. The error bars are the mean ± SD from three biological replicates.

## Supplementary Information

**Additional file 1: Figure S1**. Expression analysis of cohesin components. **Figure S2**. Analysis of the knockdown efficiency of the cohesin components using siRNA pool. **Figure S3**. Cell cycle analysis after knockdown of cohesin components. **Figure S4**. Analysis of apoptosis after knockdown of RAD21 and REC8. **Figure S5**. Chromosome compaction in spread samples and fixed whole nuclei during metaphase. **Figure S6**. Depletion of cohesin components leads to ESC differentiation. **Figure S7**. Change in chromosome structure after WAPL knockdown. **Figure S8**. Localization pattern of condensin from interphase to metaphase in ESCs and MEF. **Figure S9**. Changes in condensin intensity during the cell cycle of ESC and MEF. **Figure S10**. Uncropped western blot gel images.

**Additional file 2: Table S1**. RNA Sequencing datasets with knockdown of cohesin components.

**Additional file 3.** Review History.

## Data Availability

RNA sequencing data have been deposited in the NCBI Sequence Read Archive under accession No. SRP316512 [102]. Associated data of this manuscript, including immunofluorescence microscopy and SIM image analyses are publicly accessible under the Figshare, DOI: 10.6084/m9.figshare.19160126 [103]. All the other data generated in this study are included in the article and the Additional files.
