# Supplementary material for: Meiosis-specific cohesin complexes display essential and distinct roles in mitotic embryonic stem cell chromosomes
Source: Genome Biol. 2022 Mar 3;23:70. doi: 10.1186/s13059-022-02632-y (PMC8892811; doi:10.1186/s13059-022-02632-y)
Supplement: Supplementary file 1 — Additional file 1: Figure S1. Expression analysis of cohesin components. Figure S2. Analysis of the knockdown efficiency of the cohesin components using siRNA pool. Figure S3. Cell cycle analysis after knockdown of cohesin components. Figure S4. Analysis of apoptosis after knockdown of RAD21 and REC8. Figure S5. Chromosome compaction in spread samples and fixed whole nuclei during metaphase. Figure S6. Depletion of cohesin components leads to ESC differentiation. Figure S7. Change in chromosome structure after WAPL knockdown. Figure S8. Localization pattern of condensin from interphase to metaphase in ESCs and MEF. Figure S9. Changes in condensin intensity during the cell cycle of ESC and MEF. Figure S10. Uncropped western blot gel images. [file 13059_2022_2632_MOESM1_ESM.pdf]

Supplementary Figure

Meiosis-specific cohesin complexes display essential and distinct roles in mitotic embryonic stem cell chromosomes

Eui-Hwan Choi[1], Seobin Yoon[1,#], Young Eun Koh[1,#], Tae Kyung Hong[2], Jeong Tae Do[2], Bum-Kyu Lee[3], Yoonsoo Hahn[1], and Keun P. Kim[1,*]

[1]Department of Life Sciences, Chung-Ang University, Seoul 06974, South Korea

[2]Department of Stem Cell and Regenerative Biotechnology, Konkuk Institute of Technology, Konkuk University, Seoul 05029, South Korea

[3]Department of Biomedical Sciences, Cancer Research Center, University of Albany-State University of New York, Rensselaer, NY, USA

[#] These authors contributed equally to this work.

* To whom correspondence should be addressed.

Tel: 82-2-820-5792

Fax: 82-820-5206

E-mail: kpkim@cau.ac.kr

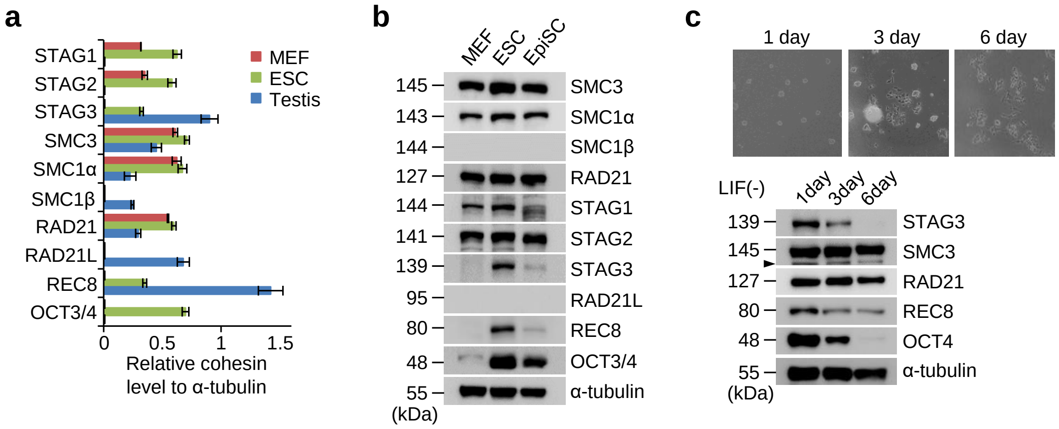

Fig. S1. Expression analysis of cohesin components.

a Quantification of expression level of cohesin factors in MEFs, ESCs, and mouse testis. The error bars are the mean ± SD from three independent experiments. **b** Expression analysis of cohesin components in MEFs, ESCs, and Epiblast stem cells (EpiSC). α-tubulin served as a protein loading control. **c** Expression of cohesin components during ESC differentiation. ESCs were differentiated by withdrawing leukemia inhibitory factor (LIF) from the culture medium. The arrowhead indicates non-specific bands.

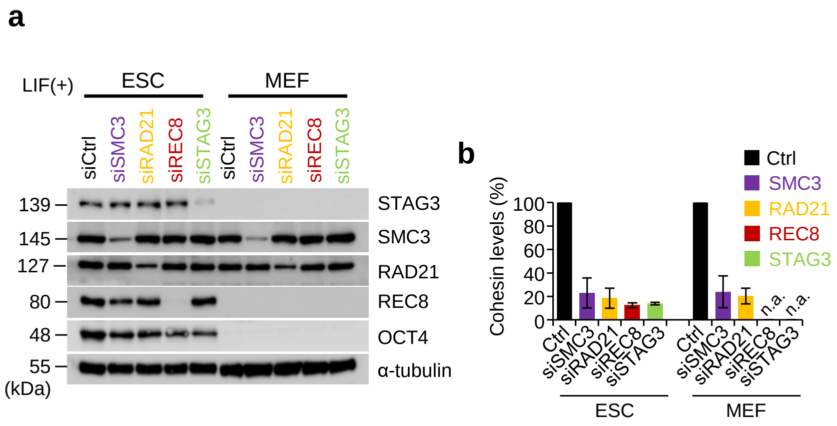

Fig. S2. Analysis of the knockdown efficiency of the cohesin components using siRNA pool.

a Knockdown of cohesin factors in ESCs and MEFs. The expression levels of SMC3, RAD21, REC8, and STAG3 were analyzed following transfection with a siRNA pool against SMC3, RAD21, REC8, and STAG3 (siSMC3, siRAD21, siREC8, and siSTAG3) by western blotting. siCtrl, the control cells with a nontargeting siRNA. α-tubulin served as a protein loading control. **b** Quantification of cohesin-knockdown efficiency. The error bars are the mean ± SD from three independent experiments.

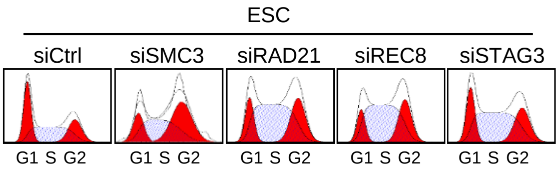

Fig. S3. Cell cycle analysis after knockdown of cohesin components.

Cell cycle profiles of ESCs treated with siSMC3, siRAD21, siREC8, and siSTAG3. Cell samples were stained with DAPI and characterized by FACS analysis.

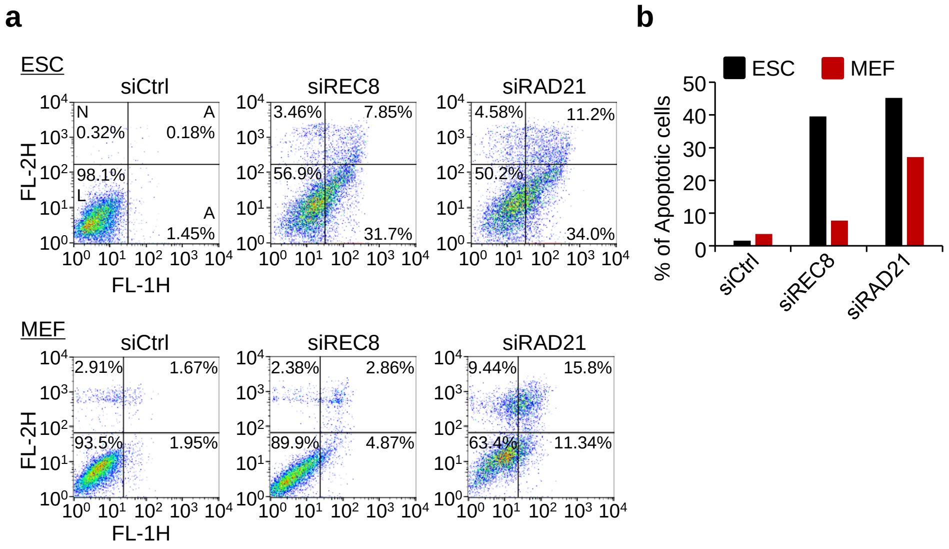

Fig. S4. Analysis of apoptosis after knockdown of RAD21 and REC8.

a Analysis of apoptosis in ESCs and MEF. The cells were incubated with siRNA in serum-free medium. The proportion of apoptotic cells was quantified with FITC-conjugated annexin V (2 µg/ml) and PI (15 µg/ml). Scatter plots indicate the distribution of FITC-conjugated annexin V and PI staining. The cells are classified as "live-cell" (bottom left), "early apoptotic-cell" (bottom right), "late apoptotic-cell" (top right) and "necrotic-cell" (top left). **b** Quantification of apoptotic cells in ESCs and MEFs.

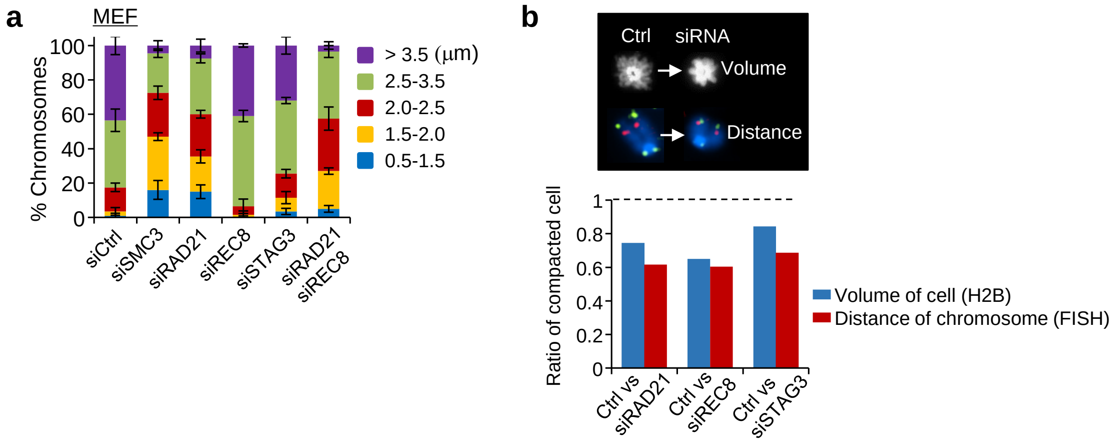

Fig. S5. Chromosome compaction in spread samples and fixed whole nuclei during metaphase.

a Quantification of chromosome lengths. The length of the chromosomes in MEF was measured by calculating the distance between both sides of the telomere probes. The error bars are the mean ± SD from three independent experiments. **b** Quantification of chromosome volume in the fixed whole nuclei (H2B) and distance in spread samples hybridized with telomeric probes and locus-specific probes. The volume and distance from metaphase chromosomes in cells depleted of mitotic/meiotic cohesin components showed similar reductions in proportion.

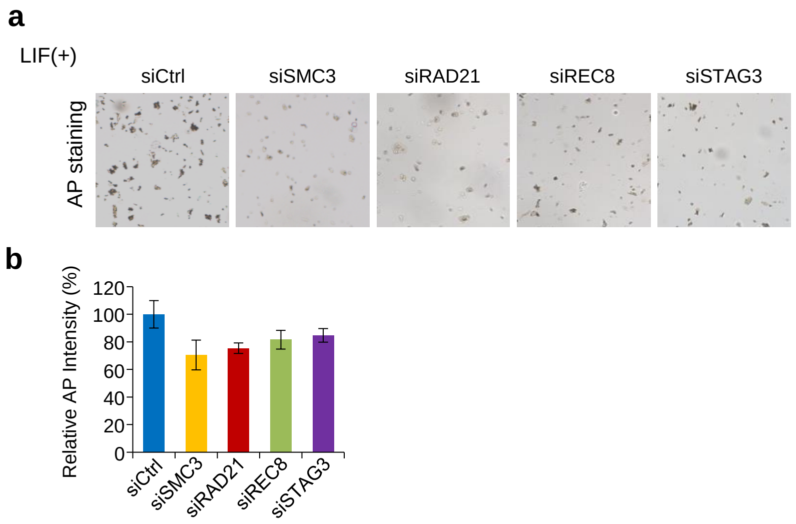

Fig. S6. Depletion of cohesin components leads to ESC differentiation.

a Analysis of alkaline phosphatase staining following transfection with a siRNA pool against SMC3, REC8, RAD21, and STAG3 in ESCs. **b** Quantification of alkaline phosphatase staining. The percentage of AP-positive colonies is shown on bar graphs. The error bars are the mean ± SD from three independent experiments.

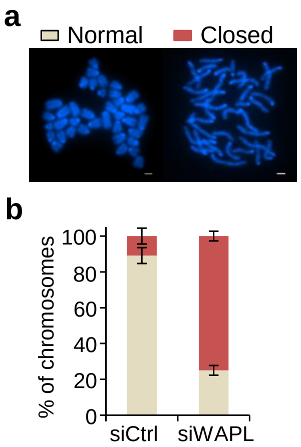

Fig. S7. Change in chromosome structure after WAPL knockdown.

a Representative images showing normal and closed chromosomes in ESCs. Scale bars are 2.5 μm. **b** Chromosome spreads of ESCs, including normal and closed chromosomes. The error bars are the mean ± SD from three independent experiments.

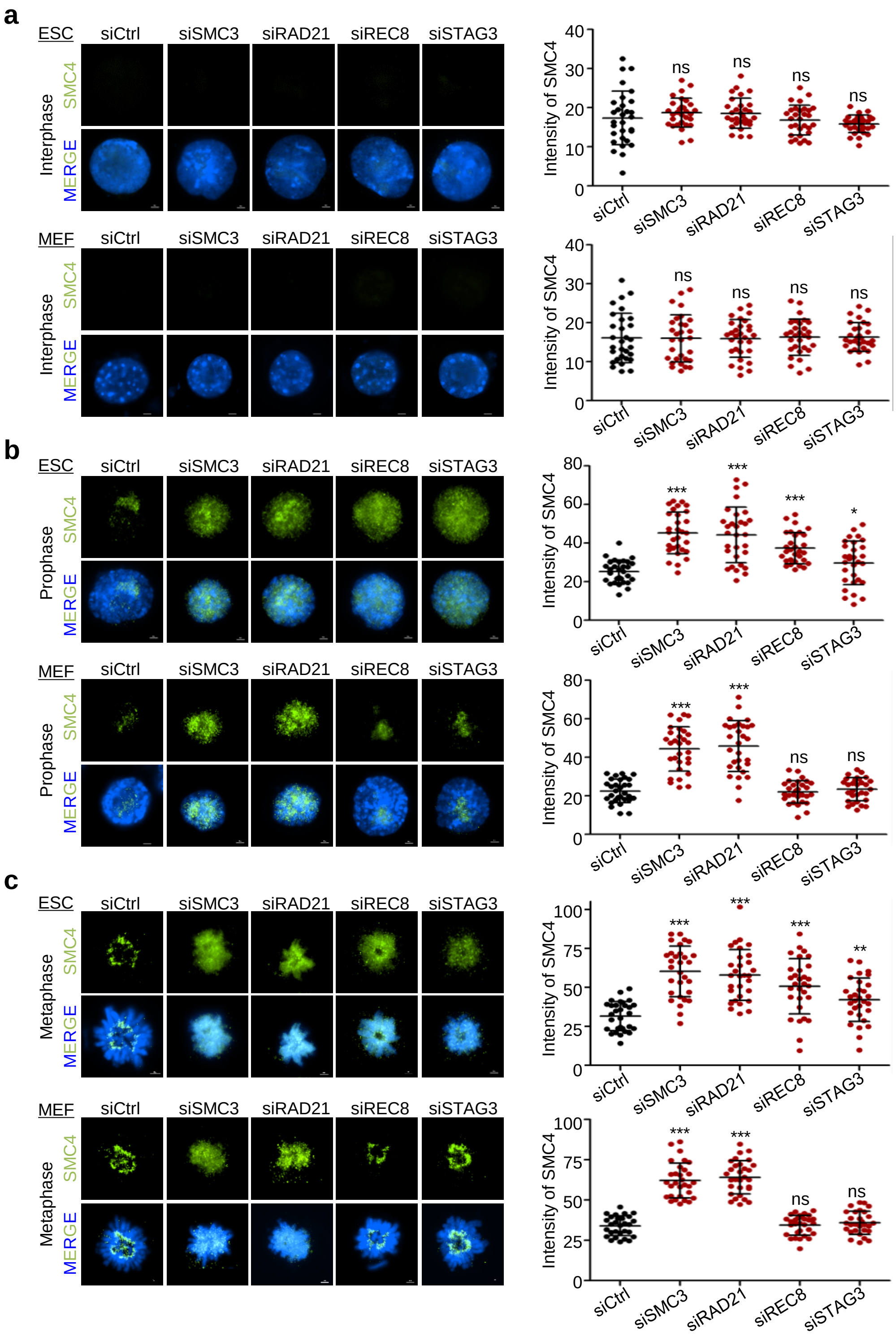

Fig. S8. Localization pattern of condensin from interphase to metaphase in ESC and MEF.

a–c Analysis of condensin intensity from interphase to metaphase in ESCs and MEF. Cells were immunostained with an antibody against SMC4 and counterstained them with DAPI. Scale bars are 2.5 µm. Staining intensity was analyzed using the Nikon NIS software. P-values (paired two-tailed t-test) were calculated using GraphPad Prism 5 software. ns: not-significant, *P < 0.5, **P < 0.01, and ***P < 0.001.

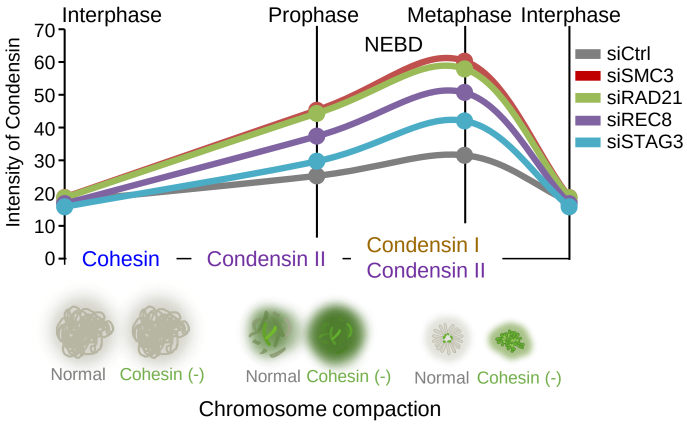

Fig. S9. Changes in condensin intensity during the cell cycle of ESC and MEF.

Analysis of condensin intensity at interphase, prophase, and metaphase in ESC. Intensity values of condensin were quantified from supplementary figure 8. NEBD, Nuclear envelope breakdown.

a

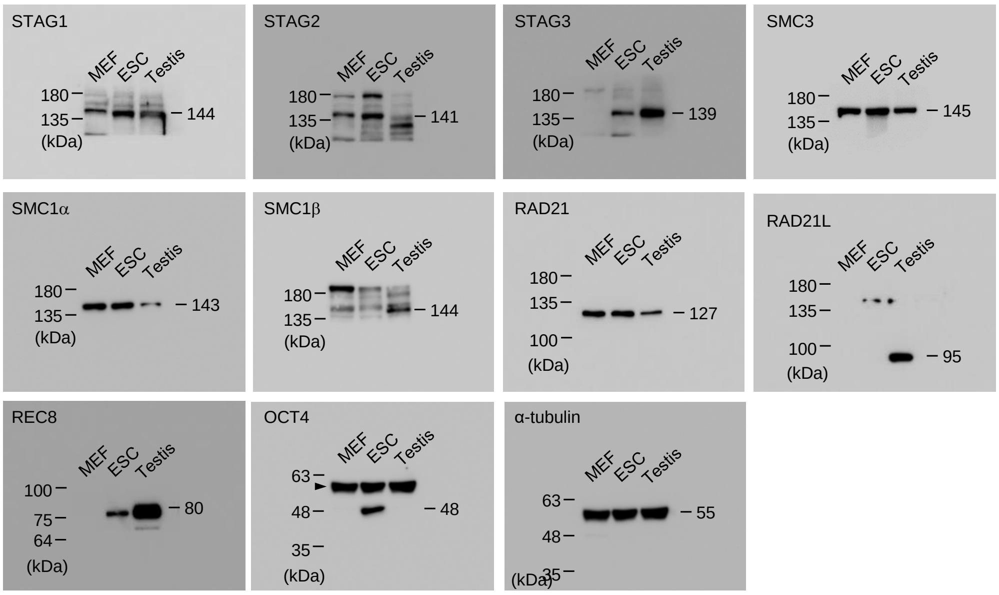
STAG1
MEF ESC Testis
180
135
(kDa)
144
STAG2
141
STAG3
139
SMC3
145
SMC1α
143
SMC1β
144
RAD21
127
RAD21L
95
REC8
80
OCT4
48
α-tubulin
55

b

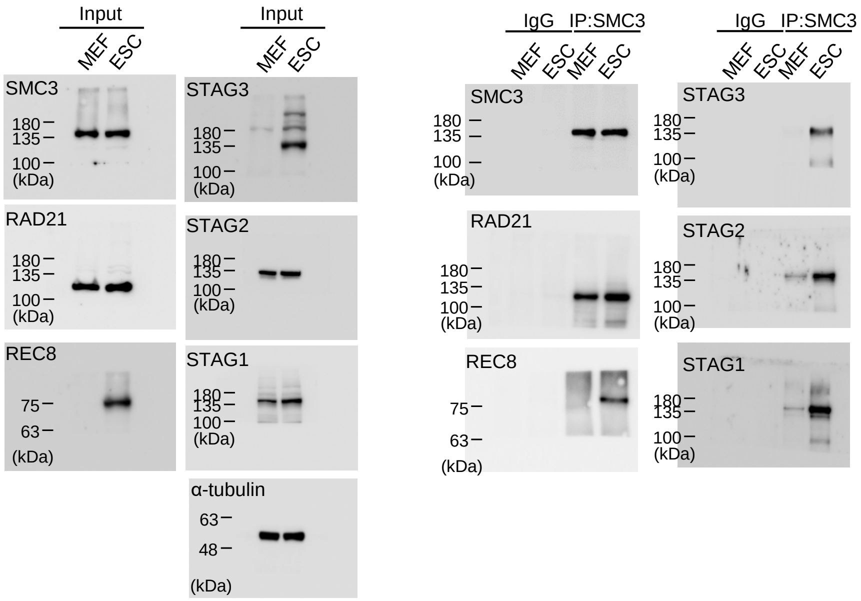
Input
MEF ESC
SMC3
RAD21
REC8
STAG3
STAG2
STAG1
α-tubulin
IgG IP:SMC3
MEF ESC MEF ESC
(kDa)

c

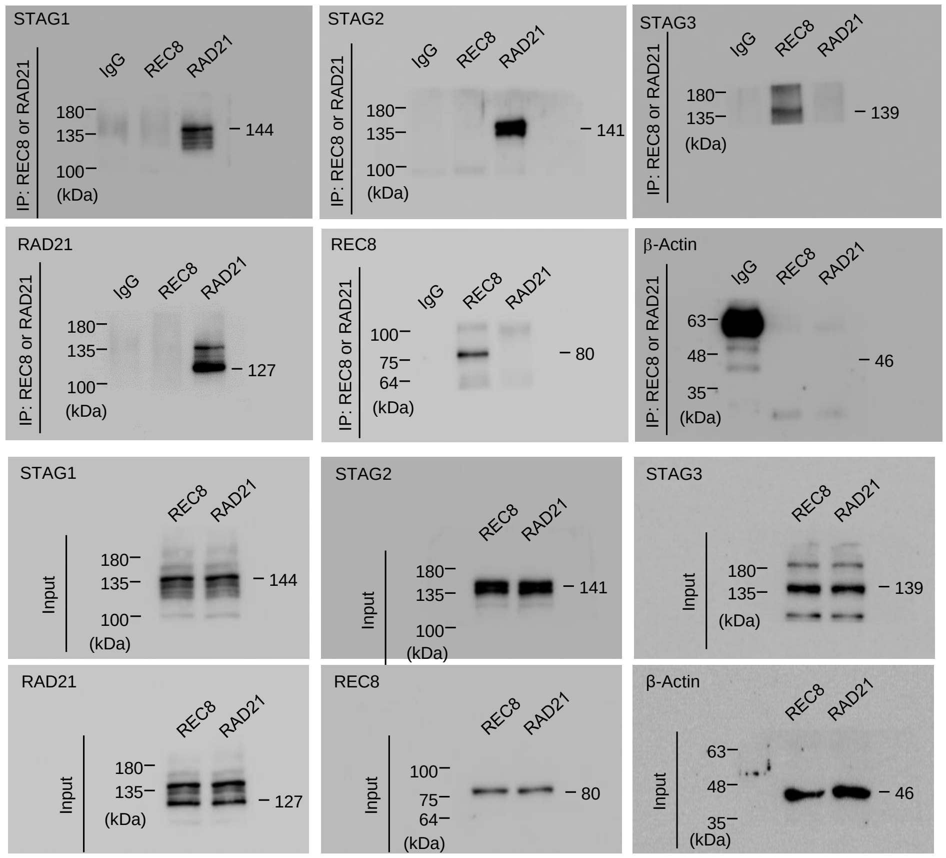
STAG1
STAG2
STAG3
RAD21
REC8
β-Actin
IP: REC8 or RAD21
Input
IgG
REC8
RAD21
(kDa)
144
141
139
127
80
46

d

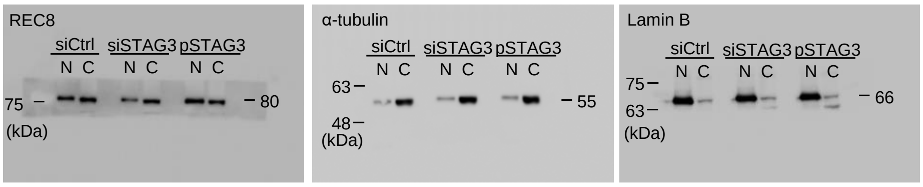
REC8
α-tubulin
Lamin B
siCtrl
siSTAG3
pSTAG3
N C
(kDa)
80
55
66

e

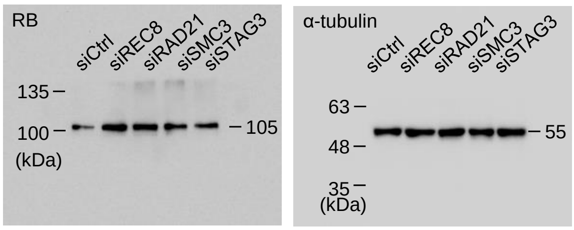
RB
siCtrl
siREC8
siRAD21
siSMC3
siSTAG3
135
100
(kDa)
105
α-tubulin
63
48
35
(kDa)
55

f

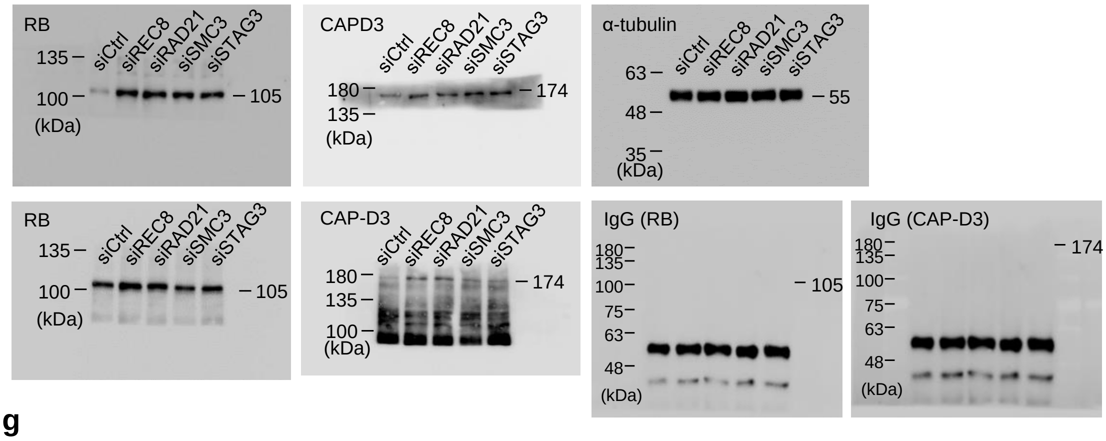
RB
siCtrl
siREC8
siRAD21
siSMC3
siSTAG3
135
100
(kDa)
105
CAPD3
180
135
(kDa)
174
α-tubulin
63
48
35
(kDa)
55
CAP-D3
100
IgG (RB)
75
IgG (CAP-D3)

g

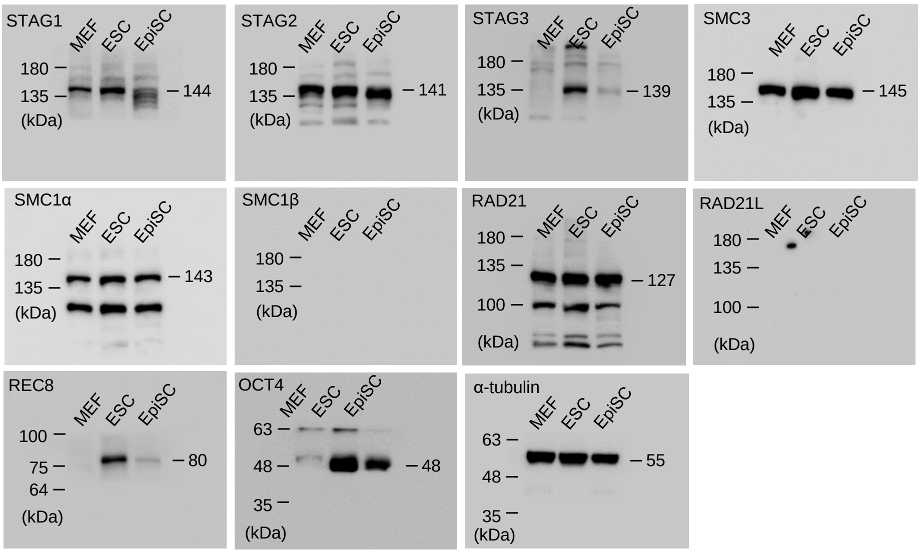
STAG1
MEF
ESC
EpiSC
180
135
(kDa)
144
STAG2
141
STAG3
139
SMC3
145
SMC1α
143
SMC1β
RAD21
100
127
RAD21L
REC8
75
64
80
OCT4
63
48
35
α-tubulin
55

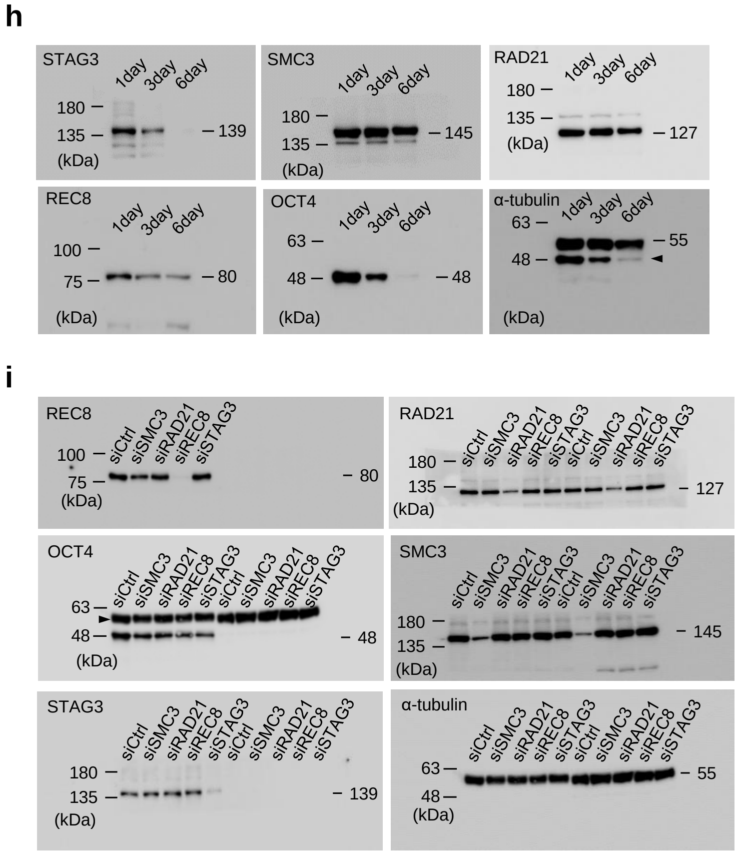

Fig. S10. Uncropped western blot gel images. a Corresponds to Fig. 1b; **b** Corresponds to Fig. 1d; **c** Corresponds to Fig. 1i; **d** Corresponds to Fig. 1k; **e** Corresponds to Fig. 4c; **f** Corresponds to Fig. 4e; **g** Corresponds to Fig. S1b; **h** Corresponds to Fig. S1c; **i** Corresponds to Fig. S2a. The arrowhead indicates non-specific bands.
