# Supplementary material for: Meiosis-specific cohesin complexes display essential and distinct roles in mitotic embryonic stem cell chromosomes
Source: Genome Biol. 2022 Mar 3;23:70. doi: 10.1186/s13059-022-02632-y (PMC8892811; doi:10.1186/s13059-022-02632-y)
Supplement: Supplementary file 2 — Additional file 2: Table S1. RNA Sequencing datasets with knockdown of cohesin components. [file 13059_2022_2632_MOESM2_ESM.pdf]

Supplementary Table 1. Up- or down-retulated gene list after knockdown of cohesin components.

A. Upregulated gene list after knockdown of SMC3, RAD21, and REC8.

Gene symbol	Fold change			Average of normalized data (log2)				Average of raw data			
	siRec8 /siControl	siRad21 /siControl	siSMC3 /siControl	siControl	siRec8	siRad21	siSMC3	siControl	siRec8	siRad21	siSMC3
Rb1	2.702	3.349	2.759	1.504	2.938	3.248	2.968	1.832	6.644	8.523	6.802
Cyr61	6.957	6.534	7.080	1.343	4.142	4.051	4.167	1.533	16.609	15.629	16.905
Sdc4	5.813	4.674	4.016	2.964	5.504	5.189	4.970	6.798	44.259	35.604	30.235
Serpine2	5.304	6.725	5.395	1.919	4.326	4.669	4.351	2.778	19.013	24.511	19.341
Plk2	5.129	4.518	3.732	2.774	5.133	4.950	4.675	5.831	34.004	30.006	24.452
Mt2	3.233	2.839	3.785	7.986	9.679	9.492	9.907	252.392	816.647	721.423	955.671
Perp	3.138	3.170	2.889	3.321	4.970	4.985	4.851	8.980	30.271	30.774	27.770
Trp53inp1	3.131	3.374	2.720	4.691	6.337	6.445	6.135	24.801	79.657	86.424	69.017
Btg2	3.086	3.264	2.897	3.173	4.799	4.879	4.707	8.011	26.763	28.528	25.038
Ptpn14	2.951	3.309	2.829	2.619	4.180	4.345	4.119	5.135	17.077	19.380	16.318
Ccng1	2.633	1.817	1.678	5.605	7.001	6.466	6.351	47.605	126.776	87.722	80.349
Gpx3	2.442	2.209	1.896	3.763	5.051	4.906	4.685	12.557	32.065	29.077	24.644
Cald1	2.410	1.784	1.779	3.664	4.933	4.499	4.496	11.669	29.483	21.689	21.485
Gsto1	2.329	1.626	1.728	4.308	5.527	5.009	5.097	18.793	45.010	31.306	33.111
Mdm2	2.265	2.152	1.827	5.078	6.258	6.184	5.948	32.753	75.316	71.945	60.520
Plod2	2.236	2.625	2.632	2.854	4.015	4.247	4.250	6.224	15.129	18.041	17.973
Pdrg1	2.235	2.174	2.035	3.335	4.496	4.455	4.360	9.084	21.508	21.008	19.470
Tex19.1	2.215	2.415	2.174	3.308	4.455	4.580	4.428	8.894	20.875	22.986	20.456
Pisd-ps1	2.214	2.935	2.366	3.207	4.354	4.761	4.450	8.228	19.397	26.193	20.783
Snhg12	2.186	2.706	2.521	3.463	4.591	4.899	4.797	10.011	23.039	28.925	26.701
Mrgpra6	2.163	2.781	2.222	6.593	7.706	8.069	7.745	95.469	207.292	268.417	212.821
B2m	2.109	2.058	1.922	3.421	4.497	4.462	4.363	9.695	21.526	21.105	19.511
Ptk7	2.056	2.566	2.298	4.500	5.540	5.860	5.701	21.610	45.418	57.252	50.840
Gm11974	1.974	2.433	2.795	3.489	4.470	4.772	4.972	10.216	21.111	26.412	30.285
Olfr856-ps1	1.948	2.805	1.846	3.809	4.771	5.297	4.694	13.005	26.231	38.442	24.790
Rev1	1.947	1.952	1.757	3.205	4.166	4.170	4.018	8.209	16.903	17.056	15.150
Ptp4a3	1.939	1.991	1.831	3.362	4.317	4.355	4.235	9.272	18.888	19.532	17.768
Crip2	1.907	1.769	1.608	4.148	5.080	4.971	4.834	16.722	32.733	30.464	27.423
Slc26a2	1.869	1.812	2.017	3.185	4.087	4.042	4.197	8.085	15.958	15.530	17.277
Susd6	1.867	2.003	1.867	3.249	4.150	4.252	4.150	8.500	16.713	18.107	16.701
S100a11	1.863	1.816	2.024	5.827	6.725	6.688	6.845	55.730	104.529	102.454	113.519
Ece1	1.858	2.138	1.819	3.727	4.621	4.823	4.590	12.228	23.541	27.397	23.012
Lama5	1.841	3.223	2.630	3.219	4.099	4.907	4.614	8.300	16.095	29.098	23.403
Ccp110	1.813	1.821	1.948	3.507	4.365	4.372	4.469	10.358	19.558	19.765	21.075
Rbms1	1.783	1.817	1.983	3.205	4.040	4.067	4.193	8.217	15.406	15.808	17.227
Arl6ip5	1.727	1.720	2.002	4.034	4.822	4.816	5.035	15.367	27.205	27.266	31.674
Tnpo2	1.721	2.065	1.940	4.037	4.820	5.084	4.993	15.405	27.180	33.016	30.747
Eda2r	1.709	2.223	2.165	3.281	4.054	4.434	4.396	8.713	15.569	20.685	19.988
Mbnl2	1.695	2.117	1.878	3.627	4.389	4.709	4.536	11.346	19.893	25.241	22.132
Paip2b	1.673	1.887	1.848	4.356	5.098	5.272	5.242	19.455	33.167	37.766	36.726
Serpinh1	1.653	2.570	2.221	4.057	4.781	5.418	5.208	15.624	26.426	41.895	35.842
Ptgr1	1.648	1.646	1.618	3.766	4.487	4.486	4.461	12.594	21.376	21.475	20.950
Ifrd1	1.628	2.138	2.076	4.392	5.095	5.488	5.445	19.973	33.085	44.020	42.423
Plin2	1.589	1.712	1.521	3.448	4.117	4.224	4.053	9.909	16.308	17.747	15.547
Tmtc3	1.578	1.645	1.575	3.608	4.266	4.327	4.263	11.181	18.192	19.129	18.136
Cldn6	1.575	1.595	1.548	3.774	4.430	4.448	4.404	12.665	20.496	20.892	20.105
Anxa2	1.567	2.773	2.635	3.852	4.500	5.323	5.250	13.418	21.560	39.152	36.914
Stk25	1.553	2.230	1.989	3.392	4.027	4.549	4.384	9.486	15.261	22.480	19.818
Hspg2	1.522	3.283	2.255	3.905	4.511	5.620	5.078	13.964	21.734	48.328	32.662
Laptm4a	1.517	1.514	1.552	5.207	5.808	5.805	5.841	35.898	54.864	55.094	56.126

B. Downregulated gene list after knockdown of SMC3, RAD21, and REC8.

Gene symbol	Fold change			Average of normalized data (log2)				Average of raw data			
	siRec8 /siControl	siRad21 /siControl	siSMC3 /siControl	siControl	siRec8	siRad21	siSMC3	siControl	siRec8	siRad21	siSMC3
Oaz1-ps	0.474	0.004	0.047	7.818	6.741	0.006	3.420	224.432	105.700	0.009	9.648
Med8	0.666	0.546	0.630	5.306	4.720	4.432	4.639	38.519	25.289	20.663	23.822
Slc25a5	0.666	0.405	0.515	9.367	8.781	8.063	8.410	658.866	437.633	267.430	337.859
Rps14	0.665	0.445	0.510	12.563	11.976	11.395	11.591	6046.390	4015.820	2700.600	3073.470
Nr2f6	0.665	0.644	0.659	4.722	4.134	4.087	4.121	25.375	16.513	16.045	16.333
Eif2d	0.665	0.502	0.588	7.808	7.220	6.813	7.042	222.933	147.680	111.859	130.254
Ndufaf3	0.664	0.646	0.647	4.369	3.779	3.740	3.741	19.652	12.695	12.403	12.325
Ppa1	0.664	0.468	0.533	7.999	7.409	6.905	7.093	254.664	168.462	119.261	134.962
AI837181	0.664	0.478	0.606	4.974	4.384	3.909	4.251	30.408	19.819	14.072	17.974
Msto1	0.664	0.383	0.500	6.223	5.631	4.837	5.222	73.623	48.443	27.691	36.168
Cct6a	0.663	0.455	0.526	9.349	8.757	8.215	8.424	650.784	430.614	297.136	341.132
Mbd3	0.663	0.563	0.567	6.818	6.226	5.990	6.001	111.785	73.645	62.772	62.787
Eif2s2	0.663	0.424	0.531	8.270	7.677	7.034	7.356	307.461	203.126	130.477	162.178
Cuedc2	0.662	0.588	0.647	6.121	5.527	5.356	5.494	68.550	44.987	40.095	43.898
Ndufs7	0.662	0.599	0.611	6.972	6.377	6.233	6.261	124.404	81.880	74.468	75.417
Aven	0.662	0.539	0.638	5.747	5.152	4.854	5.098	52.659	34.464	28.022	33.113
Tkt	0.662	0.595	0.641	8.097	7.501	7.348	7.454	272.466	179.641	162.467	173.677
Rassf7	0.662	0.462	0.575	4.688	4.092	3.574	3.889	24.756	16.010	10.951	13.762
Bysl	0.660	0.437	0.539	5.884	5.285	4.691	4.991	58.004	37.890	24.925	30.686
Smpd2	0.660	0.460	0.547	4.270	3.671	3.149	3.401	18.281	11.705	7.903	9.524
Tead2	0.660	0.573	0.619	6.358	5.758	5.555	5.666	80.961	52.969	46.166	49.583
Maz	0.659	0.559	0.646	7.329	6.728	6.489	6.699	159.641	104.707	89.124	102.540
Rps3	0.658	0.529	0.584	10.668	10.065	9.749	9.893	1624.680	1067.465	862.603	946.001
Ndufs8	0.658	0.606	0.638	6.952	6.349	6.229	6.304	122.704	80.285	74.276	77.741
Pagr1a	0.658	0.507	0.614	5.553	4.949	4.572	4.849	45.910	29.798	22.863	27.721
Basp1	0.657	0.566	0.627	7.237	6.632	6.417	6.565	149.742	97.893	84.724	93.309
Atic	0.657	0.512	0.606	7.834	7.228	6.867	7.110	226.945	148.488	116.119	136.653
Ssbp4	0.657	0.568	0.641	6.707	6.101	5.892	6.065	103.392	67.448	58.571	65.729
Tfpt	0.657	0.419	0.506	5.207	4.600	3.953	4.224	35.891	23.188	14.536	17.618
Taf10	0.656	0.647	0.656	7.341	6.734	6.714	6.734	161.021	105.133	104.348	105.030
Pold2	0.656	0.516	0.583	7.003	6.395	6.049	6.225	127.160	82.955	65.428	73.504
Grtp1	0.656	0.640	0.651	6.028	5.419	5.383	5.409	64.188	41.669	40.865	41.354
Psph	0.655	0.587	0.604	5.672	5.062	4.905	4.944	49.945	32.310	29.056	29.679
Trmt112	0.655	0.497	0.535	7.271	6.660	6.262	6.369	153.324	99.842	76.013	81.345
Pih1d1	0.655	0.452	0.497	6.187	5.576	5.041	5.178	71.798	46.573	32.038	35.053
Mrpl11	0.654	0.428	0.508	5.403	4.790	4.179	4.426	41.266	26.590	17.182	20.421
Smarcb1	0.654	0.482	0.575	6.982	6.369	5.929	6.183	125.273	81.413	60.140	71.378
Stub1	0.653	0.542	0.620	6.512	5.898	5.629	5.822	90.149	58.463	48.643	55.384
Arl6ip4	0.653	0.440	0.550	6.098	5.484	4.913	5.237	67.453	43.628	29.232	36.561
Timm23	0.653	0.442	0.558	8.228	7.612	7.048	7.387	298.490	194.158	131.821	165.695
Psmd13	0.653	0.566	0.657	7.544	6.928	6.722	6.939	185.468	120.471	104.945	121.216
Eif3d	0.652	0.475	0.569	8.208	7.591	7.134	7.394	294.338	191.321	139.924	166.544
Rab8a	0.652	0.523	0.651	5.790	5.173	4.855	5.171	54.283	34.986	28.038	34.897
Sra1	0.651	0.563	0.647	6.408	5.787	5.579	5.779	83.830	54.082	46.960	53.699
Hnrnpab	0.650	0.425	0.554	9.477	8.857	8.244	8.626	711.120	461.300	303.145	392.603
Rnaseh2a	0.650	0.517	0.579	6.014	5.391	5.062	5.225	63.544	40.855	32.517	36.259
Rpl30	0.648	0.368	0.441	8.298	7.672	6.856	7.116	313.455	202.459	115.238	137.159
Tsen34	0.648	0.508	0.599	7.073	6.446	6.097	6.333	133.479	85.955	67.669	79.331
Dohh	0.647	0.464	0.529	6.807	6.180	5.698	5.888	110.895	71.305	51.086	57.990
Ubxn6	0.646	0.558	0.571	5.326	4.696	4.484	4.517	39.081	24.854	21.458	21.817
Gltscr2	0.646	0.397	0.490	8.156	7.526	6.823	7.127	283.887	182.778	112.647	138.219
Anp32b	0.646	0.535	0.578	9.770	9.140	8.867	8.980	871.179	561.650	467.548	502.084
Sphk2	0.646	0.485	0.605	4.090	3.459	3.046	3.365	16.018	9.973	7.287	9.264
Isyna1	0.646	0.523	0.617	6.895	6.264	5.960	6.199	117.927	75.662	61.447	72.200

Chchd1	0.645	0.579	0.627	7.248	6.615	6.460	6.575	150.824	96.728	87.307	93.967
Ruvbl1	0.645	0.451	0.549	7.722	7.088	6.572	6.858	209.943	134.712	94.479	114.529
Rabif	0.644	0.559	0.636	5.890	5.254	5.051	5.236	58.238	37.063	32.274	36.542
Nme6	0.644	0.491	0.539	5.109	4.473	4.084	4.219	33.488	21.155	16.017	17.552
Rnf187	0.643	0.423	0.506	7.455	6.819	6.213	6.472	174.339	111.583	73.438	87.419
Sgk1	0.642	0.427	0.648	8.199	7.560	6.969	7.573	292.553	187.200	124.752	188.702
Yif1b	0.641	0.583	0.666	6.320	5.678	5.541	5.734	78.808	50.069	45.713	52.020
Rdh11	0.640	0.657	0.658	4.634	3.991	4.029	4.031	23.817	14.859	15.374	15.289
Gm4737	0.640	0.468	0.586	6.017	5.373	4.921	5.245	63.691	40.323	29.396	36.788
Eif2b4	0.639	0.376	0.522	6.152	5.505	4.741	5.213	70.055	44.290	25.837	35.948
Ube2r2	0.638	0.627	0.590	5.556	4.907	4.882	4.795	46.013	28.930	28.584	26.669
Tbcb	0.638	0.329	0.481	6.806	6.156	5.204	5.751	110.770	70.144	35.989	52.652
Serbp1	0.636	0.520	0.604	8.334	7.682	7.391	7.606	321.302	203.744	167.399	193.034
Csrp2	0.635	0.401	0.499	8.353	7.699	7.034	7.350	325.698	206.179	130.503	161.554
Nr0b1	0.635	0.406	0.561	7.002	6.347	5.702	6.168	127.093	80.199	51.224	70.625
Apex1	0.634	0.352	0.500	6.629	5.972	5.122	5.631	97.913	61.610	33.949	48.359
Pmvk	0.633	0.563	0.570	6.384	5.724	5.555	5.573	82.424	51.732	46.183	46.424
Gjb5	0.633	0.349	0.384	4.777	4.116	3.258	3.397	26.385	16.303	8.602	9.497
Tgif1	0.633	0.480	0.629	7.377	6.717	6.319	6.709	165.106	103.921	79.099	103.251
Carkd	0.633	0.522	0.655	4.350	3.690	3.413	3.739	19.383	11.872	9.686	12.309
Uqcc2	0.631	0.597	0.633	8.393	7.729	7.649	7.734	334.899	210.581	200.454	211.123
Srm	0.631	0.363	0.446	8.185	7.520	6.724	7.020	289.673	182.018	105.097	128.306
Bckdk	0.631	0.523	0.612	5.154	4.489	4.218	4.444	34.572	21.390	17.674	20.692
Rangrf	0.630	0.382	0.461	7.154	6.487	5.764	6.038	141.308	88.477	53.542	64.460
Ict1	0.630	0.403	0.464	6.493	5.826	5.183	5.385	89.022	55.570	35.464	40.621
Nubp1	0.629	0.471	0.599	5.560	4.891	4.475	4.820	46.135	28.594	21.316	27.140
Psmb5	0.629	0.515	0.579	8.701	8.032	7.744	7.912	414.815	260.010	214.188	238.876
Ppif	0.627	0.473	0.548	6.287	5.613	5.208	5.420	77.018	47.811	36.085	41.645
Lap3	0.627	0.406	0.485	8.222	7.548	6.920	7.179	297.272	185.583	120.503	143.329
Ahcy	0.626	0.439	0.557	6.648	5.973	5.461	5.804	99.222	61.655	43.212	54.674
Smarcd3	0.626	0.559	0.510	5.324	4.648	4.485	4.353	39.036	24.007	21.462	19.356
H2afz	0.625	0.411	0.533	10.534	9.855	9.251	9.626	1480.770	922.698	610.214	786.346
Mars	0.624	0.620	0.662	6.856	6.176	6.168	6.262	114.770	71.089	71.143	75.447
Tmem160	0.623	0.441	0.461	6.419	5.738	5.239	5.302	84.532	52.223	36.904	38.309
Ube2m	0.623	0.372	0.494	7.757	7.075	6.329	6.740	215.187	133.472	79.693	105.474
Mrto4	0.623	0.496	0.560	7.220	6.538	6.207	6.383	147.982	91.659	73.150	82.126
Gadd45gip1	0.622	0.465	0.509	4.963	4.279	3.857	3.987	30.154	18.361	13.542	14.802
Fiz1	0.621	0.412	0.532	5.983	5.296	4.703	5.073	62.194	38.192	25.131	32.538
Samm50	0.621	0.481	0.568	8.275	7.588	7.219	7.460	308.476	190.947	148.481	174.397
Mpnd	0.621	0.594	0.651	5.899	5.211	5.147	5.278	58.619	35.938	34.549	37.672
Tfeb	0.621	0.407	0.558	5.441	4.753	4.145	4.598	42.392	25.888	16.751	23.126
Cops6	0.620	0.433	0.464	8.454	7.765	7.245	7.345	349.283	215.892	151.182	160.969
Rpl7a	0.620	0.475	0.573	11.805	11.116	10.731	11.001	3574.160	2211.820	1704.995	2040.590
Pgls	0.620	0.630	0.557	6.911	6.221	6.244	6.066	119.257	73.375	75.052	65.773
Acat2	0.619	0.490	0.499	6.495	5.803	5.464	5.492	89.092	54.678	43.304	43.827
Gpa33	0.619	0.521	0.521	5.533	4.841	4.592	4.594	45.275	27.586	23.208	23.063
Dkc1	0.617	0.487	0.551	7.685	6.989	6.647	6.826	204.649	125.719	99.551	112.001
Rpl18a	0.617	0.500	0.551	10.421	9.724	9.422	9.561	1368.930	842.674	687.363	751.442
Prmt6	0.616	0.486	0.618	4.018	3.319	2.979	3.325	15.193	8.957	6.908	8.985
Uxt	0.615	0.605	0.641	5.023	4.322	4.297	4.380	31.478	18.953	18.721	19.752
Hist2h2bb	0.615	0.448	0.596	6.497	5.796	5.338	5.752	89.271	54.433	39.588	52.696
Tubb3	0.615	0.551	0.545	4.096	3.395	3.236	3.221	16.092	9.492	8.454	8.290
Dph3	0.615	0.508	0.590	4.381	3.679	3.404	3.620	19.810	11.776	9.618	11.252
Tmem11	0.615	0.486	0.592	6.207	5.506	5.166	5.452	72.828	44.316	35.028	42.614
Agpat2	0.615	0.598	0.614	5.044	4.342	4.302	4.340	31.968	19.230	18.792	19.177
Nudt8	0.613	0.500	0.510	4.708	4.003	3.707	3.737	25.122	14.990	12.107	12.288

Cyc1	0.609	0.505	0.583	8.375	7.660	7.390	7.596	330.646	200.667	167.307	191.766
Tars	0.605	0.550	0.600	6.918	6.194	6.056	6.181	119.827	71.997	65.752	71.302
2810428I15Rik	0.605	0.517	0.612	6.856	6.132	5.903	6.148	114.765	68.934	59.058	69.634
Snx17	0.605	0.532	0.562	6.014	5.289	5.103	5.183	63.560	37.983	33.490	35.187
Phb2	0.605	0.431	0.493	9.153	8.428	7.937	8.133	567.823	342.390	244.942	278.677
Tmem234	0.605	0.512	0.568	6.959	6.233	5.993	6.143	123.278	74.012	62.902	69.396
Plp2	0.604	0.521	0.533	6.923	6.197	5.982	6.015	120.247	72.162	62.434	63.445
Mvk	0.604	0.489	0.509	5.320	4.592	4.287	4.347	38.918	23.059	18.585	19.270
Tuba1b	0.604	0.400	0.473	9.581	8.853	8.258	8.502	764.021	460.191	306.256	360.066
Nsun5	0.604	0.413	0.510	4.418	3.689	3.140	3.445	20.356	11.867	7.847	9.849
Slc25a1	0.602	0.442	0.517	6.398	5.666	5.218	5.447	83.234	49.623	36.361	42.452
Mepce	0.601	0.476	0.571	4.798	4.064	3.728	3.989	26.798	15.681	12.299	14.821
Fdft1	0.600	0.541	0.524	5.838	5.101	4.951	4.904	56.142	33.236	30.043	28.834
Abhd17a	0.598	0.493	0.566	6.501	5.759	5.479	5.679	89.484	53.008	43.759	50.051
Tomm40	0.597	0.403	0.508	7.951	7.207	6.638	6.973	246.229	146.365	98.968	124.119
Tpm3	0.597	0.439	0.577	8.633	7.888	7.446	7.838	395.607	235.285	173.955	226.975
Hax1	0.597	0.515	0.556	7.349	6.604	6.392	6.503	161.927	96.033	83.267	89.383
Arhgdia	0.596	0.477	0.561	7.777	7.031	6.709	6.944	218.143	129.434	103.982	121.616
Thap11	0.594	0.469	0.584	5.350	4.600	4.257	4.575	39.762	23.188	18.193	22.746
Timm13	0.594	0.427	0.481	6.782	6.030	5.556	5.727	108.945	64.182	46.200	51.774
Naa10	0.592	0.340	0.451	7.361	6.604	5.804	6.211	163.239	96.029	55.079	72.798
Pycr2	0.592	0.408	0.575	6.884	6.126	5.590	6.085	116.997	68.672	47.334	66.638
Rsph1	0.591	0.387	0.466	4.401	3.643	3.031	3.299	20.106	11.460	7.201	8.804
Mybl2	0.589	0.420	0.495	9.296	8.534	8.043	8.283	627.187	368.617	263.740	309.243
Ccdc22	0.587	0.526	0.611	4.432	3.664	3.505	3.721	20.567	11.641	10.388	12.140
Nelfe	0.587	0.402	0.530	6.550	5.781	5.235	5.633	92.606	53.855	36.804	48.440
Tsta3	0.586	0.417	0.513	6.041	5.271	4.778	5.079	64.805	37.503	26.536	32.667
Ak2	0.586	0.553	0.625	7.831	7.060	6.977	7.152	226.431	132.068	125.387	140.717
Cenpb	0.586	0.421	0.459	6.127	5.355	4.879	5.004	68.815	39.811	28.521	30.979
Tuba1a	0.585	0.429	0.449	6.876	6.103	5.654	5.720	116.301	67.535	49.540	51.495
Fam213b	0.584	0.546	0.510	5.488	4.713	4.613	4.518	43.838	25.150	23.560	21.821
Inhbb	0.584	0.498	0.500	6.290	5.514	5.283	5.290	77.168	44.568	38.075	37.976
Map2k2	0.583	0.416	0.526	6.484	5.707	5.217	5.557	88.452	51.094	36.335	45.902
Fkbp4	0.583	0.375	0.437	9.390	8.611	7.976	8.196	669.276	389.026	251.562	291.218
Cisd3	0.582	0.417	0.450	7.779	6.999	6.519	6.629	218.458	126.607	91.025	97.591
Mrpl12	0.582	0.344	0.469	8.081	7.300	6.542	6.988	269.539	156.166	92.484	125.425
Hist1h4i	0.582	0.530	0.511	5.130	4.349	4.215	4.162	34.011	19.327	17.634	16.832
Galk1	0.581	0.495	0.548	7.490	6.707	6.475	6.621	178.600	103.226	88.275	97.082
Epb4.1l4a	0.580	0.418	0.505	5.716	4.930	4.459	4.729	51.527	29.396	21.074	25.429
Eif6	0.579	0.357	0.453	8.329	7.541	6.841	7.185	320.224	184.761	114.038	143.972
Gdf3	0.577	0.435	0.607	5.529	4.736	4.328	4.809	45.132	25.586	19.159	26.937
Mvb12a	0.577	0.372	0.475	6.157	5.363	4.731	5.083	70.277	40.034	25.659	32.764
Alkbh7	0.576	0.552	0.478	5.439	4.643	4.583	4.374	42.367	23.918	23.049	19.660
Hist1h4k	0.575	0.397	0.543	5.840	5.042	4.507	4.958	56.222	31.860	21.822	29.974
Ufc1	0.575	0.507	0.542	7.206	6.406	6.227	6.321	146.517	83.591	74.143	78.674
Qdpr	0.570	0.529	0.539	7.046	6.236	6.126	6.155	130.997	74.174	69.100	69.981
Mical1	0.565	0.529	0.644	4.262	3.439	3.342	3.628	18.175	9.814	9.177	11.320
Ctu2	0.565	0.455	0.558	4.445	3.621	3.308	3.604	20.768	11.268	8.937	11.113
Exosc5	0.562	0.464	0.555	7.768	6.936	6.659	6.919	216.727	121.097	100.373	119.565
Lgals3	0.558	0.336	0.476	6.016	5.175	4.442	4.945	63.647	35.029	20.815	29.692
Eif4e2	0.558	0.376	0.470	8.059	7.218	6.649	6.971	265.480	147.491	99.685	123.949
Psat1	0.558	0.562	0.547	7.511	6.669	6.679	6.641	181.252	100.509	101.852	98.438
Hist1h4h	0.557	0.390	0.508	4.587	3.743	3.228	3.609	23.006	12.354	8.405	11.155
Sars	0.550	0.485	0.526	6.941	6.078	5.899	6.015	121.788	66.355	58.860	63.422
Use1	0.549	0.372	0.468	6.902	6.036	5.476	5.806	118.538	64.460	43.680	54.724
Rpp25	0.547	0.406	0.433	7.000	6.129	5.699	5.791	126.907	68.787	51.115	54.189
Tmem238	0.544	0.442	0.562	4.048	3.169	2.869	3.217	15.526	7.971	6.328	8.267
Mvd	0.544	0.456	0.469	5.674	4.795	4.542	4.581	50.005	26.680	22.379	22.840
Cnpy2	0.540	0.483	0.570	7.106	6.217	6.055	6.293	136.604	73.191	65.695	77.162
Ptpmt1	0.530	0.585	0.565	4.814	3.898	4.040	3.991	27.106	13.865	15.501	14.843
Yars	0.524	0.593	0.629	6.896	5.963	6.142	6.227	118.022	61.224	69.865	73.665
Rdm1	0.521	0.381	0.464	6.767	5.826	5.375	5.660	107.839	55.570	40.635	49.371
Ldlr	0.516	0.527	0.556	5.542	4.587	4.619	4.696	45.549	22.962	23.649	24.814
Gcat	0.508	0.410	0.474	5.811	4.835	4.525	4.733	55.091	27.459	22.103	25.491
March9	0.501	0.463	0.562	4.201	3.203	3.092	3.370	17.388	8.186	7.555	9.304
Pgp	0.498	0.313	0.416	6.355	5.349	4.680	5.090	80.811	39.647	24.728	32.938
Mrps34	0.493	0.308	0.465	6.659	5.639	4.961	5.555	99.932	48.716	30.266	45.821
Nsdhl	0.491	0.531	0.522	5.027	4.000	4.115	4.089	31.581	14.962	16.381	15.955
Fdps	0.453	0.421	0.416	8.185	7.044	6.937	6.921	289.726	130.611	121.929	119.673
Usf2	0.396	0.533	0.621	4.931	3.594	4.024	4.244	29.478	11.045	15.323	17.887
Vax2	0.343	0.272	0.323	5.501	3.959	3.626	3.871	44.264	14.512	11.388	13.581
