# Supplementary material for: Meiosis-specific cohesin complexes display essential and distinct roles in mitotic embryonic stem cell chromosomes
Source: Genome Biol. 2022 Mar 3;23:70. doi: 10.1186/s13059-022-02632-y (PMC8892811; doi:10.1186/s13059-022-02632-y)
Supplement: Supplementary file 3 — Additional file 3. Review History. [file 13059_2022_2632_MOESM3_ESM.docx]

Review History

**First round of review**

**Reviewer 1**

**Are you able to assess all statistics in the manuscript, including the appropriateness of statistical tests used?**

Yes

**Comments to author:**

The authors show that REC8-STAG3-containing cohesin regulates chromosome topological properties and maintains sister chromatid cohesion in ESC. By siRNA knockdown, they showed that REC8-STAG3-cohesin is required for sister chromatid cohesion even in the presence of Rad21- cohesion. Depletion of REC8cohesin leads to higher incidence of premature sister chromatid separation, delayed replication fork progression, and hyper condensation of chromosome as a result of excess RB-condensin II loading. Furthermore, REC8-cohesinlocalizes at centromere regions more abundantly than RAD21-cohesin. Thus, they propose that REC8-STAG3-cohesin has additional roles in concert with mitotic RAD21- cohesin on ESC chromosomes.

Overall, the manuscript provides new insights on meiotic cohesin in ESC. However, the data are still premature as they do not investigate these events with sufficient precision or depth. Another concern in this study is that physiological relevance of REC8-STAG3-containing cohesin in the ESC are not well presented. It is uncertain whether those observation can be observed only in naïve state ESC but not in primed state ESC such as EpiSC or further differentiated state. Thus, it would be nice if they could investigate further whether the specific function of meiotic cohesin involves in pluripotency of ESC. Otherwise, those observation could be artefact of in vitro culture of ESC. Therefore, the authors describe much more than what was actually shown in this study, which could be a problem over the story in this manuscript. Although this study is potentially interesting in the field, several concerns listed below should be properly addressed before it can be considered for publication.

Page4 Introduction and others through the main texts

The author should narrate what was previously shown or not, about the meiotic cohesin in mice. References are not update and not properly cited. Thus, several key studies on mouse meiotic cohesin seem to be missing in the introduction and through the main text. For example, the statement "RAD21/SCC1 can partially substitute for REC8 to support chromosome axes and interhomolog partner interactions during recombination" is not support by Ref 35. Ref36 is related to budding yeast Rec8 but not to mouse REC8. Thus, this reference is not appropriate in this context. So, the author should state the function of budding yeast Rec8 and mouse REC8 separately. They should properly and comprehensively describe the role of mouse meiotic cohesin in the introduction without bias to particular studies, and/or cite an appropriate review.

Line 103-104, Fig1b

The authors state that the meiosis-specific cohesin components REC8 and STAG3 were abundantly expressed in ESCs but not in MEFs. It is not clear why their analysis was limited only to REC8 and STAG3 without examining other meiotic cohesin subunits, raising a question whether SMC1b and RAD21L are expressed in ESC. Further, how extent STAG1 and STAG2 contribute to cohesin complex in ESC is unclear. The authors should comprehensively analyze other meiotic subunits, SMC1b, RAD21L and STAG1/STAG2. Molecular weight should be shown along the immunoblots.

Fig1c

The IP experiment is missing an important negative control. Negative control IP should be done along with SMC3 IP. Also, STAG3 and STAG1/STAG2 blots should be shown.

Line 121 Fig1d

Fold change of RNA-seq (ESC vs MEF) does not tell the exact expression levels of those genes compared. The authors should show the expression levels of Rec8, Rad21, Stag3, and other genes encoding cohesion subunits in ESC and MEF by RT-qPCR.

Line 136

The authors state that REC8 and STAG3 can corporate to promote the formation of cohesion complex. It remains unclear whether REC8 and mitotic STAG1/STAG2 form a complex.

STAG1/STAG2 blots should be shown in Fig1i.

Line 144

The authors state that REC8 and STAG3 plays a role in replication fork progression. Later they also showed that Rb is upregulated in siRec8, siSMC3 and siRad21 in Fig4d. Since Rb negatively regulates S phase progression, it is not clear whether REC8 and STAG3 are directly involved in replication fork progression. Therefore, physiological relevance of this observation in ESC is yet to be determined. The authors should discuss more about the interpretation of this data.

Line 175, Line 183-185

Either knock down of REC8 or RAD21 show around 25% of precocious separation of sisters in ESC (Fig3d). There are two possibilities that (1) both REC8 and RAD21 involve in cohesion in a given cell or (2) RAD21 involves in cohesion in a cells, and REC8 does in another cell. According to Fig3d image, all the sisters are separated in siRec8, which would suggest RAD21 does not involve in cohesion in this particular cell. While the authors assume that REC8 and RAD21 independently contribute to sister chromatid cohesion, it is still not clear whether cohesion will be additively affected when cells are subjected to siRec8 plus siRad21. The authors should show double knock down of REC8 or RAD21. Also, they should discuss the interpretation of the data in Fig3d.

Line 209-211 Fig4g

The IP experiment is missing an important negative control. Negative control IP should be done along with Rb IP. Also, negative control of immunoblot (tubulin) should be shown.

Line 213-221 Fig4h, i

The authors claim that hyper compaction of chromosome in siRec8 is attributed to condensing II and Rb. This idea should be confirmed by double knockdown by siRB and si Rec8 to see whether hyper compaction of chromosome is rescued but sister chromatid are still separated. In Fig4h, it is not clear whether sister chromatids are separated in siRec8. Do the authors show representative image of siRec8? Please clarify by showing clear image.

Line 246-247 Fig5g

The authors claim that ChIP analysis of the centromere satellite sequences revealed that REC8 was more abundant than RAD21 in centromere regions (Fig. 5g). Since this experiment lacks a control, they should perform ChIP analysis of other genome regions.

Line 288-289 Fig7a

It is not clear whether the data shown in Fig7a derived from reanalysis of the previous ChIP-seq data, since there is no description in the method. If so, please describe it.

Line 292, Fig7b

Please indicate exactly where the binding region of condensing and cohesin. Also please show raw data of the SMC4, SMC3, RAD21, REC8 ChIP in ESC along with control ChIP.

Line 311

"REC8- or STAG3-null ESC" is not correct. They used REC8- or STAG3-knockdown ESC. Please rephrase it.

Uncropped gel images for all the WB should be shown.

**Reviewer 2**

**Are you able to assess all statistics in the manuscript, including the appropriateness of statistical tests used?**

Yes

**Comments to author:**

Cohesin is a ring-shaped complex that has the ability to encircle strands of DNA. The ability of cohesin to entrap DNA is utilized in maintaining cohesion between sister chromatids following DNA replication, but also in establishing loop domains in interphase cells. Different variants of the cohesin complex containing different accessory STAG proteins perform distinct functions and have different phenotypes when mutated in cells and animals. Cohesin has been implicated in a wide range of functions: sister chromatid cohesion, interphase loop establishment, DNA repair, and meiotic recombination. Additionally, during meiosis a dramatically different form of cohesin is responsible for regulating cohesion between sister chromatids and promoting homologous recombination. The meiotic form of cohesin replaces Rad21 with Rec8 and STAG1/2 with STAG3. It has been assumed that Rec8 and STAG3 are only necessary in meiotic cells, although it is known that they can function in mitotic cells. Choi et al. study the functions of the meiotic accessory proteins Rec8 and STAG3 in mitotic mouse ES cells. Surprisingly, they find that both Rec8 and STAG3 have important roles in mitotic ES cells and that loss of the cohesin complex results in hyperactive loading of the condensin complex onto mitotic chromosomes. Overall, this study presents some interesting and surprising observations about the function of the meiotic cohesion complex and could be of general interest to the chromosome biology community. However, this manuscript lacks a cohesive focus and overstates the conclusions of many different experiments. Furthermore, there are many experiments that are incomplete and not convincing. Additional experimentation and a significant rewriting of the manuscript are necessary to make this a valuable contribution to the chromosome biology field.

Major Points

1. This manuscript is a combination of two different lines of investigation about the cohesin complex, but neither story is finished. This manuscript would be much more effective if the authors provided more supporting evidence for one or both of these stories.

a. The authors show that Rec8 and STAG3 are expressed in ES cells and are required for normal cohesin function in ES cells. The authors report that Rec8/STAG3 cohesin constitute a small proportion of the total cohesin in ES cells, yet their depletion causes chromosome cohesion defects. This is a surprising result as it has been shown that chromosomes must lose >80% of cohesin in order to exhibit sister chromatid cohesion defects (PMID 30002073). More importantly, why are Rec8/STAG3 expressed in mitotic cells? There are many well-documented meiosis specific interactions performed by Rec8/STAG3. Are these important in ES cells?

b. In the second part of the manuscript the authors argue that loss of cohesin leads to upregulation of pRB, extra loading of condensin on mitotic chromosomes, and hypercondensation of chromosomes. This is also a surprising result as many studies have not documented changes in chromosome condensation upon loss of cohesin. The proposed mechanism is not rigorously tested and should be validated in greater detail (see below).

2. One of the main themes of this manuscript is that loss of all members of the cohesin complex leads to a constellation of phenotypes in ES cells, but it is not clear if all of the phenotypes are direct or indirect effects. Work over the last decade (e.g. 33318687, 21589869, 20720539) has shown that expression of cohesin and its regulators is required to maintain ES cells in an undifferentiated state. It seems possible that many of the chromosome structure changes documented here could be a result of differentiation of ES cells. Yet, the authors never mention this as a possibility and do not experimentally test if this is occurring in their siRNA-treated cells. The authors should experimentally test if siRNA-treated cells differentiate and should discuss this possibility in their manuscript.

3. Another main theme of this manuscript is that knockdown of cohesin components leads to an increase in pRB which leads to extra loading of the condensin complex onto mitotic chromosomes, yet this model is never tested. If the authors model is correct then RNAi depletion of pRB and any cohesin component at the same time should not lead to extra condensin loading and chromosome hypercondensation.

4. There are many experiments in the manuscript that are not properly quantified and presented:

a. Many immunofluorescence experiments appear to have been performed once. For these experiments (1j, 2a-f, 3g-I, 4hi, 5bi, 6efjk) the authors have calculated statistical significance between conditions using a students t-test. These experiments should be performed a minimum of 2 times in different biological replicates. Statistical significance between conditions should be calculated using the mean value from each replicate, not treating individual cells or chromosomes as a separate N. I recommend the authors use the plotting and statistical conventions discussed in this recent article (32346721).

b. In many places the authors make quantitative arguments based on Western blot data but it is not clear how they arrive at the values they present. For example, in Figure S1b they argue that there is a certain amount of knockdown for each siRNA. The authors should explicitly state how these values were obtained and include numbers from replicate experiments. The authors should quantitate pRB protein levels in Figure 4f and provide replicate data. The IP data in Figure 4g arguing that more pRB interacts with SMC4 after Rec8 knockdown needs a pRB loading control. The observed difference could result from different amounts of pRB in each IP. This data should also be provided from replicate experiments.

Minor Points.

1. There are several points where the authors should include a relevant citation.

a. P6 line 142 should cite 29724914.

b. P13 line 241 should cite 32396063.

2. Figure S1a SMC3 IP data does not include a positive control sample from the testes as shown for Rec8. This blot also needs a loading control for the amount of SMC3 IPed in each sample.

3. Figure 4e. Changes in pRB transcript levels should be validated by Q-RT-PCR in independent samples.

4. Figure 4hi. STAG3 siRNAi is omitted in this experiment. Almost all other experiments in the manuscript include STAG3 siRNA. It seems strange that it is not present here.

5. P8. Line 219. Figure 4jk do not exist.

6. The data presented in Figure 6d-k is essentially the same data shown in Figure 4hi. These data should be combined.

7. The formatting for Figure 6 is unusual. Why is the background of this figure black?

8. The formatting of Figure 7a is very difficult to understand. At a minimum this plot needs to include a track of the gene annotations as it is essentially impossible to understand as presented.

9. Figure 7b. Rec8 does not show enrichment at cohesin binding regions by ChIP and the enrichment does not increase when Rad21 is knocked down. This data is inconsistent with data presented in Figure 5.

10. There are several places where presentation of information could be improved by rephrasing:

a. Abstract line 27. REC8/Rad21 cohesin. Should be Rec8 or Rad21 cohesin to indicate that there are two different complexes and not one complex containing both Rec8 and Rad21.

b. P3 line 73 "condinsin"

c. P8 line 219. "increased by 3.9% and 1.8%" Are these values correct?

d. P10 line 270 "condensin distribution on cohesion knockdown"

**Reviewer 3**

**Are you able to assess all statistics in the manuscript, including the appropriateness of statistical tests used?**

Yes

**Comments to author:**

Cohesin is a central factor in interphase and mitotic processes. The presence of meiotic specific subunits has been known for many years and their presence in mitotic cells have been associated with tumorigenesis. In the manuscript entitled "Meiosis-Specific Cohesin Complexes Display Essential and Distinct Roles in Mitotic ESC Chromosomes", Choi et al. study the role of meiosis specific subunits, namely REC8 and STAG3 by comparing their depletion effect in ESC and MEF cells. ESC cells express a significant fraction of meiotic cohesin that is absent in MEF cells. The authors show that depletion of meiotic cohesin in ESC cells interferes with mitotic cohesion and chromosome structure. Cohesion loss results in chromatin hypercondension as a result of condensin overloading. The results are interesting and provide new understanding of the function of meiotic cohesin in mitotic cells. However, several issues need to be resolved in order to support the conclusions of the study.

Major issues:

1) The authors claim that STAG3 is required for nuclear localization (figure 1k). The data don't support this model and the conclusion is speculative. Furthermore, the stability of REC8 should be tested in the absence of STAG3 in order to disapprove of this possibility.

2) ESC cells contain a mixture of miotic and mitotic cohesins. In order to understand the results, the relative amount of each type needs to be determined. The lack of this information makes the interpretation of some results challenging. In this context, some results are not well understood. For example, in figure 2, siRAD21 and siREC8 have the same inhibition effect on DNA replication. That would suggest that the two complexes exist in a similar amount. However, siSMC3 depletes both populations but has the same effect as the individual depletions. The same issue is also puzzling in respect to the condensation results in figure 4i.

3) Another issue is the RAD51 foci experiment in figure 2. It has been suggested that meiotic specific subunits are insensitive to DNA damage, which allows the programmed DSBs during meiosis. Therefore, oמe can expect less RAD51 foci in siRAD21 cells in which REC8-cohesin is dominant.

4) REC8 is enriched in centromeres, while RAD21 is on chromosome arms (figure 5). Based on this, one can expect a higher segregation defect after REC8 depletion compared to the RAD21 depletion in ESC. This is not the case (figure 3d). In that sense, exploring the integrity of SCC in the centromere by specific probes may be informative. In addition, a control depletion of SMC3 is needed to determine the relative effect of each type of cohesin compared to complete depletion.

5) Can the authors elaborate on the spatial information that can clarify the transcription misregulation presented in figure 4d. for example, the proximity of the genes to the REC8 enriched centromeres, telomers, TAD boundaries, etc.

6) In figure 4f the authors show no change in RB levels. However, in figure 4g they show an increase in the RB-SMC4 complex. Can the molecular events that enhance the interaction should be referred to? This experiment should be repeated after si SMC3, siRAD21 and siREC8.

7) To conclude that hypercondensation depends on RB, the experiment in figure 4h-i should be repeated in ESC after siRB. The expected result is decondensation of the chromatin to control levels.

8) Can the authors comment on the potential roles of condensin I and II in the hypercondensation?

Minor issues

1) The complex description in figure 1A is not accurate. The kleisin is bound to the coiled-coil of SMC3 and not the head, as shown.

2) Figure 4a-c is related to figure 3 while the following panels in figure 4 are distinct. This data should be rearranged.

3) An interesting finding is that STAG3 has a weaker effect on hypercondensation and expression than REC8 (figure 3 d and g). In this respect, STAG3 may be more similar to STAG1 than STAG3. This point can be discussed.

4) I don't understand the model presented in figure 8e. Is there is a difference between mitotic and meiotic cohesins besides the colors?

5) Sup figure 4- the authors should differentiate between condensin II and I phases. Before and after the NMB

**Authors Response**

**Point-by-point responses to the reviewers’ comments:**

Reviewer #1: The authors show that REC8-STAG3-containing cohesin regulates chromosome topological properties and maintains sister chromatid cohesion in ESC. By siRNA knockdown, they showed that REC8- STAG3-cohesin is required for sister chromatid cohesion even in the presence of Rad21- cohesion. Depletion of REC8 cohesin leads to higher incidence of premature sister chromatid separation, delayed replication fork progression, and hyper condensation of chromosome as a result of excess RB-condensin II loading. Furthermore, REC8-cohesin localizes at centromere regions more abundantly than RAD21-cohesin. Thus, they propose that REC8-STAG3-cohesin has additional roles in concert with mitotic RAD21- cohesin on ESC chromosomes. Overall, the manuscript provides new insights on meiotic cohesin in ESC. However, the data are still premature as they do not investigate these events with sufficient precision or depth. Another concern in this study is that physiological relevance of REC8-STAG3-containing cohesin in the ESC are not well presented. It is uncertain whether those observation can be observed only in naïve state ESC but not in primed state ESC such as EpiSC or further differentiated state. Thus, it would be nice if they could investigate further whether the specific function of meiotic cohesin involves in pluripotency of ESC. Otherwise, those observation could be artefact of in vitro culture of ESC. Therefore, the authors describe much more than what was actually shown in this study, which could be a problem over the story in this manuscript. Although this study is potentially interesting in the field, several concerns listed below should be properly addressed before it can be considered for publication.

*We thank the reviewer for his/her insightful comments and appreciation of its strengths, as well as for highlighting key points that required clarification. We believe that we have appropriately answered all concerns raised by the reviewer. Please see our answers below.*

*As the reviewer suggested, we investigated the physiological relevance of REC8-STAG3-containing cohesin in EpiSC. Although EpiSCs also expressed a high level of mitotic cohesin factors that was comparable to that of MEFs or ESCs (Supplementary Figure 1b), they expressed negligible level of meiosis-specific cohesin factors, REC8 and STAG3. Additionally, we examined the expression kinetics of mitotic and meiotic cohesin during ESC differentiation. The expression levels of meiotic cohesin REC8 and STAG3 dramatically decreased upon ESC differentiation (Supplementary Figure 1c). Taken together, our results suggest that meiotic cohesin is specifically required for naïve ESCs but not for EpiSCs to maintain their primed pluripotency. We have now provided complete sets of data in Supplementary Figure 1. Further, we have updated the results section as follows: “Stem cells from epiblast of mouse embryos can exhibit ‘naïve’ and ‘primed’ pluripotent states [51]. Mouse ESCs resemble the naïve pluripotent state that occurs in the early pre-implantation embryo, while epiblast stem cells (EpiSCs) derived from the late post-implantation epiblast constitute primed pluripotent cells that differ from naïve ESCs in the expression of pluripotency-related genes [52, 53, 54, 55]*. *To investigate whether meiotic cohesin could contribute to sister chromatid cohesion and chromosome morphogenesis in the primed states of pluripotency, we examined the expression of REC8 and STAG3 in mouse EpiSCs. EpiSCs exhibited a high level of expression of mitotic cohesin factors comparable to that of MEFs or ESCs, whereas the expression levels of meiosis-specific cohesin factors REC8 and STAG3 were only marginally detectable in EpiSCs (Additional file 1: Fig. S1). Additionally, to test whether ESC differentiation changes the expression kinetics of REC8 and STAG3, we differentiated ESCs by withdrawing leukemia inhibitory factor from the culture medium. Compared with the control ESCs, cells undergoing differentiation expressed significantly lower levels of REC8 and STAG3 (Additional file 1: Fig. S1). Taken together, our findings indicate that REC8 and STAG3 are required in naïve ESCs but not in primed ESCs.”*

Page4. Introduction and others through the main texts The author should narrate what was previously shown or not, about the meiotic cohesin in mice. References are not update and not properly cited. Thus, several key studies on mouse meiotic cohesin seem to be missing in the introduction and through the main text. For example, the statement "RAD21/SCC1 can partially substitute for REC8 to support chromosome axes and interhomolog partner interactions during recombination" is not support by Ref 35. Ref36 is related to budding yeast Rec8 but not to mouse REC8. Thus, this reference is not appropriate in this context. So, the author should state the function of budding yeast Rec8 and mouse REC8 separately. They should properly and comprehensively describe the role of mouse meiotic cohesin in the introduction without bias to particular studies, and/or cite an appropriate review.

*We appreciate the comments. We have re-written the relevant parts of the introduction in the revised version of our manuscript. Please see below. “During meiosis, meiotic cohesin components, including -kleisin REC8 (the human homolog of yeast Rec8) and its interacting subunits play essential roles not only in sister chromatid cohesion but also genetic recombination and chromosome morphogenesis [9, 10, 35, 36 37, 38-41]. Mammalian REC8 physically interacts with STAG3, a meiosis-specific STAG protein, to maintain its stability and to associate with the axis of the meiotic chromosomes [42, 43]. REC8 forms a cohesin complex with SMC1 and SMC3, but not with SMC1α, and localizes to the axial element (AE) of the synaptonemal complex (SC) throughout meiotic prophase I [37]. In mice lacking REC8, both male and female meiocytes exhibit failure of meiotic prophase I progression [35]. Further, REC8 prevents local AE separation and illegitimate SC formation between sister chromatids [44]. The partial loss of cohesin causes centromere decompaction and kinetochore fragmentation, particularly in aged oocytes [45, 46]. In the budding yeast Saccharomyces cerevisiae, cohesin associates with the chromosome axis during meiotic prophase and promotes SC formation, homolog template choice, and crossover-specific recombination [26, 39, 47, 48]. Furthermore, the meiotically expressed Scc1/Mcd1 (RAD21 in vertebrates) can partially substitute Rec8 to support chromosome axes and interhomolog partner interactions during recombination [47]. However, Rec8 can also carry out meiosis-specific activities through its interactions with meiosis-specific proteins [47].”*

Line 103-104, Fig1b The authors state that the meiosis-specific cohesin components REC8 and STAG3 were abundantly expressed in ESCs but not in MEFs. It is not clear why their analysis was limited only to REC8 and STAG3 without examining other meiotic cohesin subunits, raising a question whether SMC1b and RAD21L are expressed in ESC. Further, how extent STAG1 and STAG2 contribute to cohesin complex in ESC is unclear. The authors should comprehensively analyze other meiotic subunits, SMC1b, RAD21L and STAG1/STAG2. Molecular weight should be shown along the immunoblots.

*We appreciate the suggestion. We analyzed the expression levels of all cohesin components including STAG1, STAG2, SMC1, and RAD21L. We included the expression data in our revised manuscript and edited the text follow: “The mitotic cohesin components SMC3, SMC1α, STAG1, STAG2, and RAD21 were expressed in both ESCs and MEFs; remarkably, the meiosis-specific cohesin components REC8 and STAG3, except for RAD21L and SMC1, were abundantly expressed in ESCs but not in MEFs (Fig. 1b).”*

Fig1c The IP experiment is missing an important negative control. Negative control IP should be done along with SMC3 IP. Also, STAG3 and STAG1/STAG2 blots should be shown.

*We agree with the reviewer. We have provided the results including the negative control and STAG1/2/3 blots in the updated version of our manuscript (Fig. 1d).*

Line 121 Fig1d Fold change of RNA-seq (ESC vs MEF) does not tell the exact expression levels of those genes compared. The authors should show the expression levels of Rec8, Rad21, Stag3, and other genes encoding cohesion subunits in ESC and MEF by RT-qPCR.

*We analyzed the expression levels of cohesin components in ESCs and MEF through western blot and qPCR. This data has now been included in Figure 1c.*

Line 136 The authors state that REC8 and STAG3 can corporate to promote the formation of cohesion complex. It remains unclear whether REC8 and mitotic STAG1/STAG2 form a complex. STAG1/STAG2 blots should be shown in Fig 1i.

*The requested data has now been provided in Figure 1i. Further, the following changes were made to our manuscript: “REC8 physically interacted with STAG3 but not with STAG1 or STAG2 (Fig. 1i), suggesting that REC8 and STAG3 can cooperate to promote the formation of cohesin complexes in ESCs.”*

Line 144 The authors state that REC8 and STAG3 plays a role in replication fork progression. Later they also showed that Rb is upregulated in siRec8, siSMC3 and siRad21 in Fig4d. Since Rb negatively regulates S phase progression, it is not clear whether REC8 and STAG3 are directly involved in replication fork progression. Therefore, physiological relevance of this observation in ESC is yet to be determined. The authors should discuss more about the interpretation of this data.

*We agree that the issues raised by the reviewer must be clarified. This interpretation has been discussed in the relevant section of our revised manuscript. Moreover, we have included the following references: Bosco et al., 2001 and Angus et al., 2004. “Although the roles of REC8 in the regulation of chromosome segregation and genetic recombination have been reported during meiosis[47], there has been no definitive study on its role during the mitotic cell cycle. Here, we found that knockdown of the REC8 or RAD21 resulted in a significant increase in the proportion of apoptotic cells and a delay in DNA replication in ESCs. Thus, both REC8-cohesin and RAD21-cohesin contribute to faithful DNA replication and participate in DNA repair pathways during mitosis. However, given that RB is a negative regulator of S-phase progression, accumulation of RB upon knockdown of cohesin components might inhibit the cell cycle progression of ESCs [100, 101]. We thus could not determine whether REC8-cohesin and RAD21-cohesin directly regulate replication fork progression or indirectly by upregulating RB. Nevertheless, considering the observations, it is reasonable to claim that the cohesin complexes composed of REC8 and RAD21 are functionally linked to cell cycle regulation by playing an essential role in the maintenance of chromosome structural stability and cohesion-mediated cell cycle progression of ESCs.”*

Line 175, Line 183-185 Either knock down of REC8 or RAD21 show around 25% of precocious separation of sisters in ESC (Fig3d). There are two possibilities that (1) both REC8 and RAD21 involve in cohesion in a given cell or (2) RAD21 involves in cohesion in a cells, and REC8 does in another cell. According to Fig3d image, all the sisters are separated in siRec8, which would suggest RAD21 does not involve in cohesion in this particular cell. While the authors assume that REC8 and RAD21 independently contribute to sister chromatid cohesion, it is still not clear whether cohesion will be additively affected when cells are subjected to siRec8 plus siRad21. The authors should show double knock down of REC8 or RAD21. Also, they should discuss the interpretation of the data in Fig3d.

*We analyzed the specificity of the binding patterns of RAD21 and REC8 on mitotic prometaphase-like chromosomes via immunofluorescence microscopy. According to structured illumination microscopy (SIM) imaging, RAD21 and REC8 were located at adjacent sites but predominantly at non-overlapping sites on the chromosome arms (Figs. 5a and c). Therefore, we concluded that the two proteins were involved in cohesion in a given cell. Further, double knockdowns of REC8/RAD21 were also conducted. The knockdown of these two αkleisin cohesin components in ESCs increased the rates of precocious sister chromatid separation compared to each single knockdown. This new data have been included in Fig. 3d.*

Line 209-211 Fig4g The IP experiment is missing an important negative control. Negative control IP should be done along with Rb IP. Also, negative control of immunoblot (tubulin) should be shown.

*We have provided the missing loading control in Fig. 4e. Line 213-221 Fig4h, i*

The authors claim that hyper compaction of chromosome in siRec8 is attributed to condensing II and Rb. This idea should be confirmed by double knockdown by siRB and si Rec8 to see whether hyper compaction of chromosome is rescued but sister chromatid are still separated.

*We confirmed that chromosome hypercompaction was rescued through double knockdown of RB and cohesin factors, whereas sister chromatids were still separated. These data have now been provided in Figure 4f-h. We have now incorporated the reviewer's suggestions in Figs. 4f-h and the relevant section has been modified as follows: “Additionally, we confirmed that double knockdown of RB and cohesin factors rescued chromosome hypercompaction, whereas sister chromatids remained separated (Figs. 4f-h).”*

In Fig4h, it is not clear whether sister chromatids are separated in siRec8. Do the authors show representative image of siRec8? Please clarify by showing clear image.

*We agree. This correction has been implemented in the revised manuscript*.

Line 246-247 Fig5g The authors claim that ChIP analysis of the centromere satellite sequences revealed that REC8 was more abundant than RAD21 in centromere regions (Fig. 5g). Since this experiment lacks a control, they should perform ChIP analysis of other genome regions.

*RAD21 and REC8 were located at adjacent sites but predominantly at nonoverlapping sites on chromosome arms. Particularly, we confirmed that REC8 but not RAD21 strongly accumulated in the centromere region after WAPL knockdown, as determined through various analyses including immunofluorescence microscopy and ChIP analysis. However, we found that this phenomena specifically occurred under the WAPL depletion condition and that REC8-cohesin may be specifically sensitive to cohesin release factor WAPL in ESCs. We predicted that these results are caused by an unknown modification of REC8 or a change in a specific signal molecule when WAPL is depleted. Thus, it would be more appropriate and even more interesting to assess the localization and role of REC8 in normal condition. We have now provided the localization analysis for REC8 and RAD21 on the WAPL+ condition in Figs. 5a-c. We are pleased to have identified this effect, as it provides novel insights into the potential direct functions of REC8 in ESC chromosomes. Our figures have now been improved based on these considerations.*

Line 288-289 Fig7a It is not clear whether the data shown in Fig7a derived from reanalysis of the previous ChIP-seq data, since there is no description in the method. If so, please describe it.

*The description is now provided in the legend of Fig. 7a. Moreover, we have included the reference (Kagey et al., 2010).*

Line 292, Fig7b Please indicate exactly where the binding region of condensing and cohesin. Also please show raw data of the SMC4, SMC3, RAD21, REC8 ChIP in ESC along with control ChIP.

*We indicated the binding regions of cohesin and condensin in Fig. 7a. Furthermore, we have now provided the dataset of ChIP data in Fig. 7c.*

Line 311 "REC8- or STAG3-null ESC" is not correct. They used REC8- or STAG3-knockdown ESC. Please rephrase it.

*We apologize for this mistake. It has been corrected.*

Uncropped gel images for all the WB should be shown.

*We have now provided complete blot sets for the western blot analysis in Supplementary Fig 10. R*

Reviewer #2:

Summary Cohesin is a ring-shaped complex that has the ability to encircle strands of DNA. The ability of cohesin to entrap DNA is utilized in maintaining cohesion between sister chromatids following DNA replication, but also in establishing loop domains in interphase cells. Different variants of the cohesin complex containing different accessory STAG proteins perform distinct functions and have different phenotypes when mutated in cells and animals. Cohesin has been implicated in a wide range of functions: sister chromatid cohesion, interphase loop establishment, DNA repair, and meiotic recombination. Additionally, during meiosis a dramatically different form of cohesin is responsible for regulating cohesion between sister chromatids and promoting homologous recombination. The meiotic form of cohesin replaces Rad21 with Rec8 and STAG1/2 with STAG3. It has been assumed that Rec8 and STAG3 are only necessary in meiotic cells, although it is known that they can function in mitotic cells. Choi et al. study the functions of the meiotic accessory proteins Rec8 and STAG3 in mitotic mouse ES cells. Surprisingly, they find that both Rec8 and STAG3 have important roles in mitotic ES cells and that loss of the cohesin complex results in hyperactive loading of the condensin complex onto mitotic chromosomes. Overall, this study presents some interesting and surprising observations about the function of the meiotic cohesion complex and could be of general interest to the chromosome biology community. However, this manuscript lacks a cohesive focus and overstates the conclusions of many different experiments. Furthermore, there are many experiments that are incomplete and not convincing. Additional experimentation and a significant rewriting of the manuscript are necessary to make this a valuable contribution to the chromosome biology field.

*We are grateful to the reviewer for his/her insightful comments on our manuscript. The reviewer’s comments were extremely helpful, particularly as he/she pointed out certain points of logic that we had failed to make explicit in the previous version of our manuscript. We believe that we have answered all concerns appropriately. Please see our answers below*.

Major Points 1. This manuscript is a combination of two different lines of investigation about the cohesin complex, but neither story is finished. This manuscript would be much more effective if the authors provided more supporting evidence for one or both of these stories. a. The authors show that Rec8 and STAG3 are expressed in ES cells and are required for normal cohesin function in ES cells. The authors report that Rec8/STAG3 cohesin constitute a small proportion of the total cohesin in ES cells, yet their depletion causes chromosome cohesion defects. This is a surprising result as it has been shown that chromosomes must lose >80% of cohesin in order to exhibit sister chromatid cohesion defects (PMID 30002073).

*We appreciate the reviewer’s comment. Although the cohesin levels in a single ES cell were not determined, cohesin-depleted ESCs exhibited defects in sister chromatid separation, as well as chromosome hypercompaction. A previous study had also reported that the depletion of Rad21 by siRAD21 treatment decreased chromosome cohesion, while significantly increasing the number of sister chromatids with resolved arms and precocious sister chromatid separation events (Panigrahi et al., 2011). These results are consistent with our findings. Particularly, similar levels of defects in sister chromatid separation were observed upon RAD21 depletion. However, due to the essential role of cohesin during cell division, complete cohesin removal results in cell death. Thus, knockdown of cohesin complex components by RNAi was used to investigate the non-essential functions of the complex in ESCs. To confirm the levels of cohesin components upon siRNA treatment on ESCs, we analyzed the mRNA and protein levels of RAD21, REC8, SMC3, and STAG3 and updated the data in the revised manuscript. As shown in Supple Fig. 2, ~ 76% efficiency was achieved in RAD21, REC8, SMC3, and STAG3, which was comparable to an 80% cohesion loss. Transfection of ESCs with siRNAs targeting RAD21, REC8, STAG3, or SMC3 resulted in significant depletion of both the mRNA and protein without causing overt cell death. However, as the reviewer pointed out, RAD21 or REC8 cohesin still remained after REC8 or RAD21 depletion. Considering these results, it is thought that ESCs require a greater amount of cohesin than normal somatic cells, or that each of the klesin cohesin subunits has an ESC-specific function. Protein analysis after cohesin knockdown has been included in the revised version of our manuscript (Supplementary Figure 2).*

More importantly, why are Rec8/STAG3 expressed in mitotic cells? There are many well-documented meiosis specific interactions performed by Rec8/STAG3. Are these important in ES cells?

*STAG3 is required for REC8 stabilization/accumulation onto the meiotic chromosome axes in oocytes. REC8/STAG3-cohesin complexes have a critical role in supporting meiotic chromosome structure and functions. Cohesin is implicated in a wide range of functions, including DNA replication and repair, chromosome segregation, genome compartmentalization, and transcription regulation. In this study, we found that ESCs, unlike somatic cells, abundantly express REC8 and STAG3, which are components of meiotic cohesin, suggesting that they may play important roles in ESCs. Similar to mitotic cohesin, our findings indicated that meiotic cohesin also plays essential roles in chromosome segregation (Figure 1e-h) and replication (Figure 2) in mouse ESCs. Additionally, we observed that depletion of REC8 or STAG3 resulted in ESC proliferation defects and promoted apoptosis, which was likely due to defects in the cell cycle, DNA replication, and/or DNA repair. These results indicate that meiotic cohesin is required for the self-renewal and pluripotency of ES cells. Given that several previous studies have demonstrated that mitotic cohesin is involved in establishing and maintaining the pluripotency of ESCs via association of pluripotency gene regulation (Ding et al. 2009; Hu et al. 2009; Kagey et al. 2010, Nitzsche et al., 2011), it is reasonable to speculate that meiotic cohesin is also implicated in pluripotency gene regulation. ESCs may require the expression of specific transcription factors accompanied by a particular chromosome organization, which is mediated by cohesin to maintain a balance between pluripotency and the capacity for rapid differentiation. Moreover, it is also possible that miotic and meiotic cohesin regulates different gene targets in ESCs. Further studies could be conducted in the future to dissect the detailed mechanisms underlying meiotic cohesin-mediated gene expression in ESCs.*

b. In the second part of the manuscript the authors argue that loss of cohesin leads to upregulation of pRB, extra loading of condensin on mitotic chromosomes, and hypercondensation of chromosomes. This is also a surprising result as many studies have not documented changes in chromosome condensation upon loss of cohesin. The proposed mechanism is not rigorously tested and should be validated in greater detail (see below).

*We appreciate the reviewer’s insightful comments. We hope that we have significantly improved the clarity of our manuscript. Please see our detailed answers below.*

2. One of the main themes of this manuscript is that loss of all members of the cohesin complex leads to a constellation of phenotypes in ES cells, but it is not clear if all of the phenotypes are direct or indirect effects. Work over the last decade (e.g. 33318687, 21589869, 20720539) has shown that expression of cohesin and its regulators is required to maintain ES cells in an undifferentiated state. It seems possible that many of the chromosome structure changes documented here could be a result of differentiation of ES cells. Yet, the authors never mention this as a possibility and do not experimentally test if this is occurring in their siRNA-treated cells. The authors should experimentally test if siRNA-treated cells differentiate and should discuss this possibility in their manuscript.

*We agree with the fact that ESCs require the expression of specific transcription factors to maintain a balance between pluripotency and the capacity for rapid differentiation. Young and colleagues (2010) reported that depletion of cohesin (SMC1α and SMC3) and mediator complexes reduced the level of the ESC transcription factor Oct4, a master regulator of the pluripotent state, in cells that remained viable during the course of the experiment. In our study, we confirmed that SMC3- and RAD21-depleted cells expressed lower levels of Oct4 compared with control ESCs. To test whether changes in the expression of this pluripotency gene in cohesindepleted cells also occurred in meiotic cohesin subunits, the same experiments were conducted using REC8- and STAG3-depleted ESCs and similar expression levels of the pluripotency marker were detected. Alkaline phosphatase (AP) staining indicated a reduction of AP-positive cells for the depletion of all cohesin factors, thus confirming the loss of pluripotency upon cohesin depletion. Therefore, the expression levels of cohesin could be associated with ESC states as the reviewer pointed out. These new findings have been included in our revised manuscript.*

*Under normal conditions, both ESCs and MEFs exhibit similar changes in chromosome structure throughout the cell cycle process. Further, chromosome structure changes such as chromosome hypercompaction, which appear after cohesin depletion in ESCs, exhibited the same trends as in MEFs. Therefore, a decrease in the expression of the pluripotency marker Oct4 caused by cohesin depletion would not have a direct effect on chromosomal structure change. We have included the following revisions in our manuscript: “Previous studies have shown that the expression of cohesin and its regulators contribute to the maintenance of ESCs in an undifferentiated state [9, 67, 68]. Moreover, our findings indicated that SMC3-, RAD21-, REC8-, and STAG3-depleted cells expressed lower levels of Oct4 (a pluripotency marker) compared with control ESCs (Additional File 1: Fig. s2). Further, alkaline phosphatase (AP) staining showed a reduction in AP stainingpositive cells for cohesin depletion, suggesting the loss of pluripotency upon cohesin knockdown (Additional File 1: Fig. s6). Thus, the chromosome structural changes observed in cohesin-depleted ESCs might have resulted from ESC pluripotency loss. However, under normal conditions, both ESCs and MEFs exhibit similar chromosome structural changes throughout the cell cycle process. Furthermore, chromosome structural changes such as chromosome hypercompaction, which occur after cohesin depletion in ESCs, show the same tendency as in MEF. Therefore, a decrease in the expression of pluripotency marker caused by cohesin depletion would not have a direct effect on chromosomal structure changes.”*

3. Another main theme of this manuscript is that knockdown of cohesin components leads to an increase in pRB which leads to extra loading of the condensin complex onto mitotic chromosomes, yet this model is never tested. If the authors model is correct then RNAi depletion of pRB and any cohesin component at the same time should not lead to extra condensin loading and chromosome hypercondensation.

*We agree. Cohesin depletion induces elevated RB expression and the resulting abundance of RB-condensin complexes actively promotes chromosome hypercompaction. Additionally, we confirmed that chromosome hypercompaction was rescued through double knockdown of RB and cohesin factors, whereas sister chromatids were still separated. These data have now been provided in Figure 4f-h.*

4. There are many experiments in the manuscript that are not properly quantified and presented: a. Many immunofluorescence experiments appear to have been performed once. For these experiments (1j, 2af, 3g-I, 4hi, 5bi, 6efjk) the authors have calculated statistical significance between conditions using a students ttest. These experiments should be performed a minimum of 2 times in different biological replicates. Statistical significance between conditions should be calculated using the mean value from each replicate, not treating individual cells or chromosomes as a separate N. I recommend the authors use the plotting and statistical conventions discussed in this recent article (32346721).

*We appreciate these valuable suggestions. The data in the revised manuscript were visualized using SuperPlots and our statistical approaches were thoroughly described in our manuscript.*

b. In many places the authors make quantitative arguments based on Western blot data but it is not clear how they arrive at the values they present. For example, in Figure S1b they argue that there is a certain amount of knockdown for each siRNA. The authors should explicitly state how these values were obtained and include numbers from replicate experiments. The authors should quantitate pRB protein levels in Figure 4f and provide replicate data. The IP data in Figure 4g arguing that more pRB interacts with SMC4 after Rec8 knockdown needs a pRB loading control. The observed difference could result from different amounts of pRB in each IP. This data should also be provided from replicate experiments.

*We agree with these observations. As the reviewer pointed out, the effects of siRNA-mediated cohesin gene inhibition should be evaluated to determine whether mitotic or meiotic cohesin components are involved in cellular processes of ESCs at different levels. Therefore, the protein levels of cohesin components were quantified from three independent experiment datasets after siRNA treatment. Further, we have now provided the IP data with a pRB loading control in Figure 4e.*

*Minor Points.*

1. There are several points where the authors should include a relevant citation.

a. P6 line 142 should cite 29724914.

*We agree. Moreover, we have included the suggested reference (Wolf et al., 2018).*

b. P13 line 241 should cite 32396063.

*We have included the suggested reference (Golfier et al., 2020).*

2. Figure S1a SMC3 IP data does not include a positive control sample from the testes as shown for Rec8. This blot also needs a loading control for the amount of SMC3 IPed in each sample.

*Loading controls have been included in the revised version of our manuscript.*

3. Figure 4e. Changes in pRB transcript levels should be validated by Q-RT-PCR in independent samples*.*

*Quantification of pRB transcript levels via qRT-PCR analysis has now been included in our study.*

4. Figure 4hi. STAG3 siRNAi is omitted in this experiment. Almost all other experiments in the manuscript include STAG3 siRNA. It seems strange that it is not present here.

*We apologize for the confusion. STAG3 siRNA analysis has now been included.*

5. P8. Line 219. Figure 4jk do not exist.

*Our apologies, this mistake has been corrected in the revised version of our manuscript. 6*

6. The data presented in Figure 6d-k is essentially the same data shown in Figure 4hi. These data should be combined.

*The results in Figure 4h and i (now figs. 4f-h) suggest that depletion of cohesin confers elevated expression of RB and that the abundance of RB proteins actively promotes hypercompaction of chromosomes. Moreover, double knockdown of RB and cohesin factors rescued chromosome hypercompaction, whereas sister chromatids remained separated. As illustrated in Figure 6d-k, to better understand the effects of condensin distribution on cohesion knockdown, the intensity of condensin and its spatial density per chromosome unit area was measured using high-resolution 3D SIM imaging. We appreciate the reviewer’s suggestions. However, we respectfully prefer to keep our data presentation as is, as all of the alternatives we have been able to think of are even more unwieldy.*

7. The formatting for Figure 6 is unusual. Why is the background of this figure black?

*We agree. This correction has been made.*

8. The formatting of Figure 7a is very difficult to understand. At a minimum this plot needs to include a track of the gene annotations as it is essentially impossible to understand as presented.

*Thank you for your suggestions. A gene annotation track and scale bars have been included in the binding profiles.*

9. Figure 7b. Rec8 does not show enrichment at cohesin binding regions by ChIP and the enrichment does not increase when Rad21 is knocked down. This data is inconsistent with data presented in Figure 5*.*

*We agree. The ChIP experiments should be repeated a number of times to make independent comparisons. A new set of ChiP analyses was conducted from the three independent cultures of ESCs and MEFs. Additionally, fold change values have now been provided in Fig. 7c.*

10. There are several places where presentation of information could be improved by rephrasing: a. Abstract line 27. REC8/Rad21 cohesin. Should be Rec8 or Rad21 cohesin to indicate that there are two different complexes and not one complex containing both Rec8 and Rad21.

*We appreciate the comment. We have fixed this in a new manuscript.*

b. P3 line 73 "condinsin"

*We apologize for this mistake. It has been corrected.*

c. P8 line 219. "increased by 3.9% and 1.8%" Are these values correct?

*These values have been corrected in our revised manuscript.*

d. P10 line 270 "condensin distribution on cohesion knockdown"

*This correction has been made in our revised manuscript. Thank you.*

Reviewer #3:

Cohesin is a central factor in interphase and mitotic processes. The presence of meiotic specific subunits has been known for many years and their presence in mitotic cells have been associated with tumorigenesis. In the manuscript entitled "Meiosis-Specific Cohesin Complexes Display Essential and Distinct Roles in Mitotic ESC Chromosomes", Choi et al. study the role of meiosis specific subunits, namely REC8 and STAG3 by comparing their depletion effect in ESC and MEF cells. ESC cells express a significant fraction of meiotic cohesin that is absent in MEF cells. The authors show that depletion of meiotic cohesin in ESC cells interferes with mitotic cohesion and chromosome structure. Cohesion loss results in chromatin hypercondension as a result of condensin overloading. The results are interesting and provide new understanding of the function of meiotic cohesin in mitotic cells. However, several issues need to be resolved in order to support the conclusions of the study.

*We thank the reviewer for his/her insightful comments and suggestions. We believe that we have identified and corrected all of the issues pointed out by the reviewer. However, we would be happy to make further corrections if required.*

Major issues: 1) The authors claim that STAG3 is required for nuclear localization (figure 1k). The data don't support this model and the conclusion is speculative. Furthermore, the stability of REC8 should be tested in the absence of STAG3 in order to disapprove of this possibility.

*We agree that the STAG3-REC8 nuclear localization should be clearly explained. To provide detailed evidence regarding the stability of REC8, western blot analysis of nuclear and cytoplasmic proteins was conducted. These new results are reported in Figure 1j and k. “…..fractionation of cytosol and nuclear proteins from ESCs revealed that REC8 and STAG3 were present in both the nucleus and the cytosol. Upon STAG3 knockdown, the REC8 level in the nucleus was reduced by ~ 27%. However, overexpression of STAG3 resulted in higher REC8 levels in the nucleus, accounting for a more than 1.3-fold increase compared to siCtrl (Figs. 1k and l).”*

2) ESC cells contain a mixture of miotic and mitotic cohesins. In order to understand the results, the relative amount of each type needs to be determined. The lack of this information makes the interpretation of some results challenging. In this context, some results are not well understood. For example, in figure 2, siRAD21 and siREC8 have the same inhibition effect on DNA replication. That would suggest that the two complexes exist in a similar amount. However, siSMC3 depletes both populations but has the same effect as the individual depletions. The same issue is also puzzling in respect to the condensation results in figure 4i.

*We agree with the reviewer’s comments. As the reviewer pointed out, the effects of siRNA-mediated cohesin gene inhibition should be evaluated to determine whether mitotic or meiotic cohesin components are involved in the cellular processes of ESCs at different levels. Protein levels of cohesin components were quantified from three-independent experimental datasets after siRNA treatment. The results of these experiments have now been presented in Supplementary Figure 2.*

3) Another issue is the RAD51 foci experiment in figure 2. It has been suggested that meiotic specific subunits are insensitive to DNA damage, which allows the programmed DSBs during meiosis. Therefore, one can expect less RAD51 foci in siRAD21 cells in which REC8-cohesin is dominant.

*As the reviewer pointed out, our findings indicated that knockdown of REC8 and STAG3 dramatically increased the number of RAD51 and RPA foci in ESCs but not in MEFs. The mitotic cohesin components SMC3 and RAD21 were expressed in both ESCs and MEFs, whereas the meiosis-specific cohesin components REC8 and STAG3 were abundantly expressed in ESCs but not in MEFs (Fig. 1b). Furthermore, REC8/STAG3- containing cohesin is required to ensure faithful chromosome segregation and DNA replication in ESCs. 3D SIM analyses indicated that RAD21 intensity was increased in cells depleted of REC8 compared with normal cells, suggesting that RAD21-cohesin is dominant when REC8 is depleted in ESCs. Thus, we concluded that both RAD21- and REC8-containing cohesin are essential contributors to cohesion-mediated cellular progression in ESCs. The following changes have thus been made to our manuscript: “Based on these data, we further hypothesized that the knockdown of REC8–STAG3 cohesin complexes might cause the accumulation of ssDNA gaps during the deceleration of S-phase progression. We further analyzed the formation of RAD51 and replication protein A (RPA) foci after knocking down the targeted cohesin components in ESCs and MEFs with siSMC3, siRAD21, siREC8, and siSTAG3. Knockdown of REC8 and STAG3 dramatically increased the number of RAD51 and RPA foci in ESCs but not in MEFs (Figs. 2g–l). Moreover, the size of the foci in ESCs was also considerably increased compared to that in MEFs (Figs. 2k and l). Thus, the lack of cohesion established by the REC8–STAG3 cohesin complexes could cause RAD51 and RPA foci accumulation at DNA gaps in ESCs. Further, our findings indicated that REC8 knockdown resulted in a significant increase in the proportion of apoptotic cells among ESCs (Additional File 1: Fig. S4), suggesting that REC8–cohesin and RAD21-cohesin jointly contribute to cohesion-mediated cellular progression.”*

4) REC8 is enriched in centromeres, while RAD21 is on chromosome arms (figure 5). Based on this, one can expect a higher segregation defect after REC8 depletion compared to the RAD21 depletion in ESC. This is not the case (figure 3d). In that sense, exploring the integrity of SCC in the centromere by specific probes may be informative. In addition, a control depletion of SMC3 is needed to determine the relative effect of each type of cohesin compared to complete depletion.

*We agree with the issues pointed out by the reviewer. This comment was extremely helpful, as it made us address certain points of logic that we had failed to make explicit in the previous version of the manuscript. As the reviewer suggested, we have evaluated the effects of siRNA-mediated cohesin gene inhibition to determine whether mitotic or meiotic cohesin components are involved in cellular processes of ESCs at different levels. The protein levels of cohesin components were quantified from three independent experiment datasets (Supplementary Figure 2). Furthermore, fluorescence in situ hybridization (FISH) analysis was conducted to assess the effects of siRNA knockdown on the chromatid cohesion of cells that had undergone DNA replication. In ESCs and MEF, knockdown of RAD21 and SMC3 induced a higher rate of abnormal sister chromatid separation than REC8 knockdown, suggesting that RAD21-cohesin could be more abundant than REC8-cohesin. RAD21 and REC8 were located at adjacent sites but predominantly at non-overlapping sites on chromosome arms. Particularly, our findings indicated that REC8 but not RAD21 strongly accumulated in the centromere region after WAPL knockdown, as determined by various analyses including immunofluorescence microscopy and ChIP analysis. However, we found that these phenomena specifically occurred on the WAPL depletion condition and that REC8- cohesin may be specifically sensitive to cohesin release factor WAPL in ESCs. We predicted that these results are caused by an unknown modification of REC8 or a change in a specific signal molecule when WAPL is depleted. Thus, it would be more appropriate, and even more interesting, to assess the localization and role of REC8 in normal conditions. We have now provided the localization analysis for REC8 and RAD21 on the WAPL+ condition in Figure 5a. We are pleased to have identified this effect, as it provides novel insights into the potential direct functions of REC8 in ESC chromosomes.*

5) Can the authors elaborate on the spatial information that can clarify the transcription misregulation presented in figure 4d. for example, the proximity of the genes to the REC8 enriched centromeres, telomers, TAD boundaries, etc.

*We appreciate this suggestion. We could not determine the proximity of the genes to the REC8 enriched regions because there is no whole genome ChIP-seq data for REC8-cohesin in ESCs. However, we decided to analyze the relationship between gene regulation (data obtained from cohesin knockdown cells in this study) and the SMC3 enriched sites (Kagey et al., 2010) using a gene annotation analysis software (http://asia.ensembl.org/Mus_musculus/Info/Index). This re-analysis is provided below. Interestingly, SMC3 enriched regions are located near the up- and down-regulated genes (gene sets of RNA-seq in Fig. 4a). These data directly implicate cohesin in gene regulation in ESCs. We believe that the analysis of gene expression changes between REC8- and RAD21-enriched regions can be done in a future study via whole-genome ChIP-seq analysis.*

6) In figure 4f the authors show no change in RB levels. However, in figure 4g they show an increase in the RB-SMC4 complex. Can the molecular events that enhance the interaction should be referred to? This experiment should be repeated after si SMC3, siRAD21 and siREC8.

*The expression levels of RB were significantly increased in response to SMC3, RAD21, REC8, and STAG3 knockdown. We further confirmed that high levels of RB expression resulted in a strong increase in RBcondensin interaction after cohesin knockdown. Quantification plots from three independent experiments have been included in the revised manuscript (Figs. 4c-e).*

7) To conclude that hypercondensation depends on RB, the experiment in figure 4h-i should be repeated in ESC after siRB. The expected result is decondensation of the chromatin to control levels.

*We agree with this reviewer’s suggestion. Our results show that depletion of cohesin confers elevated expression of RB and that the abundance of RB-condensin complexes actively promotes chromosome hypercompaction. Additionally, we confirmed by double knockdown of RB and cohesin factors that chromosome hypercompaction was rescued but sister chromatids were still separated. These new data have now been included in Figs. 4f-h.*

8) Can the authors comment on the potential roles of condensin I and II in the hypercondensation?

*Yes. We have addressed this point in the revised version of our manuscript. “Condensins are localized along the central axes of the mitotic chromosomes to allow linkage between adjacent and/or distant DNA loops during the prophase-to-metaphase transition (Fig. 7d). Condensin I and II differentially occupy chromosome regions and cooperate in chromosome assembly from prophase to metaphase [27, 28]. Condensin II associates with chromatin simultaneously with cohesin-release and participates in chromosome condensation in the early prophase. On the other hand, condensin I is involved in chromosome condensation after nuclear envelope break down at the late prophase. Thus, RB-induced condensin II loading on ESC chromosomes might be the key process that mediates faithful chromosome morphogenesis during the early stage of chromosome compaction [32].”*

Minor issues

1) The complex description in figure 1A is not accurate. The kleisin is bound to the coiled-coil of SMC3 and not the head, as shown.

*We agree. This correction has been made in the revised version of our manuscript.*

2) Figure 4a-c is related to figure 3 while the following panels in figure 4 are distinct. This data should be rearranged.

*We completely agree. The figures have now been rearranged in the revised manuscript.*

3) An interesting finding is that STAG3 has a weaker effect on hypercondensation and expression than REC8 (figure 3 d and g). In this respect, STAG3 may be more similar to STAG1 than STAG3. This point can be discussed.

*We agree that STAG3 has a weaker effect on sister chromatid separation, chromosome compaction, and DNA replication than REC8. Previous studies have reported that STAG1 and STAG2, RAD21-interacting SA components, display partially distinct and overlapping roles in gene expression, as well as cohesin roles (Arruda et al., 2020; Viny et al., 2019). Further, STAG1 and STAG2 display nearly identical distributions across the genome, despite not interacting together in a stable complex. Thus, REC8-STAG3 cohesin may function independently of STAG1 or STAG2 containing cohesin in ESC chromosomes. These clarifications have been made in the revised version of our manuscript.*

*“ESCs simultaneously expressed both REC8 and STAG3, and these proteins were copurified from nuclear fractions. REC8-STAG3 containing cohesin plays a critical role in regulating sister chromatid cohesion and chromosome topology in ESCs. Höög and colleagues reported that the α-kleisin REC8 in meiocytes exhibited dosage-dependent STAG3 requirements and that STAG3-REC8 cohesin complexes have an important role in supporting sister chromatid cohesion and the meiotic chromosome axis [42]. Our findings indicated that ectopic expression of STAG3 resulted in higher levels of REC8 in the nucleus, accounting for a more than 1.5-fold increase compared to siCtrl. Therefore, high levels of STAG3 are required for REC8 containing cohesin stabilization and accumulation onto the ESC chromosomes. This new finding suggests that ESCs systematically express STAG3 to regulate the translocation of REC8 into the nucleus. Therefore, REC8/STAG3-containing cohesin complexes in the nucleus could function with mitotic cohesin complexes and specifically mediate ESCspecific cohesin roles. These findings build upon those of a previous study that revealed that ectopically expressed-REC8 in Hek293 cells requires STAG3 to functionally replace RAD21, the mitotic counterpart of REC8 [42].”*

4) I don't understand the model presented in figure 8e. Is there is a difference between mitotic and meiotic cohesins besides the colors?

*>> We apologize for the confusion. We meant to illustrate the distinct localization of mitotic and meiotic cohesins at cohesion sites in ESCs. This figure has been corrected in the revised version of our manuscript.*

5) Sup figure 4- the authors should differentiate between condensin II and I phases. Before and after the NMB

*We agree. This correction has been made in the revised version of our manuscript.*

**Second round of review**

**Reviewer 2**

In this work the authors make the surprising finding that the meiosis-specific cohesin components Rec8 and STAG3 are expressed and functional in ESCs. They go on to show that reduction of cohesin components through RNAi leads to hypercompaction of chromosomes mediated by increased expression of Rb and excess loading of the condensin complex onto chromosomes. In the previous review I raised a number of technical and logical issues that needed to be addressed before this study was convincing. In this revised manuscript the authors have dramatically improved the work through the inclusion of new data, substantially improved data presentation and a revised text. I commend the authors for their thorough and conscientious manuscript revision. I now support publication of the manuscript in Genome Biology. Below I raise two minor points that the authors may wish to address prior to publication.

Minor points.

P9 line 48. The authors state that they used RNA-seq after depletion of cohesin components to identify differentially expressed genes. They eventually state that they identified Rb as a factor that is upregulated after cohesin depletion, but do not directly state that Rb was identified by RNA-seq. Is Rb upregulated in RNA-seq after cohesin depletion? If so, then the authors should state this plainly and include the data in the main figure.

P12. Cohesin depletion does not affect Smc4 localization during interphase, but only in mitosis by immunofluorescence. In the ChIP experiment cohesin knockdown has a strong effect on Smc4 chromatin binding (Figure 47BC). Are these experiments conducted in mitosis? If not, why do you observe increased Smc4 chromatin binding by ChIP but not by IF after cohesin knockdown? I believe that the authors should address this issue in the text.

**Reviewer 3**

I have read the revised manuscript. The authors have made an exemplary effort in addressing all my concerns with additional informative experiments. These have greatly improved this manuscript that I can now recommend for publication.

**Authors Response**

**Point-by-point responses to the reviewers’ comments:**

In this work the authors make the surprising finding that the meiosis-specific cohesin components Rec8 and STAG3 are expressed and functional in ESCs. They go on to show that reduction of cohesin components through RNAi leads to hypercompaction of chromosomes mediated by increased expression of Rb and excess loading of the condensin complex onto chromosomes. In the previous review I raised a number of technical and logical issues that needed to be addressed before this study was convincing. In this revised manuscript the authors have dramatically improved the work through the inclusion of new data, substantially improved data presentation and a revised text. I commend the authors for their thorough and conscientious manuscript revision. I now support publication of the manuscript in Genome Biology. Below I raise two minor points that the authors may wish to address prior to publication.

*We thank the reviewer for his/her insightful comments and suggestions. We believe that we have corrected all of the issues pointed out by the reviewer.*

Minor points.

P9 line 48. The authors state that they used RNA-seq after depletion of cohesin components to identify differentially expressed genes. They eventually state that they identified Rb as a factor that is upregulated after cohesin depletion, but do not directly state that Rb was identified by RNA-seq. Is Rb upregulated in RNA-seq after cohesin depletion? If so, then the authors should state this plainly and include the data in the main figure.

*We appreciate the comments. In RNA-seq analysis, we observed that the RB was highly upregulated in response to SMC3, RAD21, REC8, and STAG3 knockdown. We have included RNA seq data and qPCR quantification in Fig. 4b. We have re-written the relevant parts of the result in the revised version of our manuscript.*

*“Among the 50 differentially upregulated genes, the RB, a binding partner of condensin, was significantly upregulated more than 2.7-fold after depletion of cohesin components (Fig. 4b; Additional file 2: Table S1).”*

P12. Cohesin depletion does not affect Smc4 localization during interphase, but only in mitosis by immunofluorescence. In the ChIP experiment cohesin knockdown has a strong effect on Smc4 chromatin binding (Figure 47BC). Are these experiments conducted in mitosis? If not, why do you observe increased Smc4 chromatin binding by ChIP but not by IF after cohesin knockdown? I believe that the authors should address this issue in the text.

*We performed the ChIP assay in mitotic chromosomes after cell synchronization. In immunofluorescence microscopy analysis, we suggested that SMC4 proteins are abundantly loaded at cohesin binding sites after knockdown of cohesin components during the prophase and metaphase. Thus, we performed the ChIP analysis in the same condition with the microscopy analysis. We have fixed the relevant part and figure legend.*

*“Therefore, we speculated that the knockdown of cohesin could cause condensin complex recruitment to cohesin binding sites. To gain insight into the proportion of the genome occupied by cohesin and condensin complexes in ESCs and MEFs, ChIP analysis was performed with REC8, RAD21, STAG3, SMC3, and SMC4 for mitotic chromosomes.”*
